# Targeted and untargeted metabolomics reveals deep analysis of drought stress responses in needles and roots of *Pinus taeda* seedlings

**DOI:** 10.3389/fpls.2022.1031466

**Published:** 2023-01-31

**Authors:** Chu Wu, Yun Wang, Honggang Sun

**Affiliations:** ^1^ College of Horticulture & Gardening, Yangtze University, Jingzhou, Hubei, China; ^2^ College of Life Sciences, Yangtze University, Jingzhou, Hubei, China; ^3^ Institute of Subtropic Forestry, Chinese Academy of Forestry, Fuyang, Zhejiang, China

**Keywords:** drought tolerance, phytohormones, fatty acids, neurotransmitters, sugars, amino acids, organic acids, flavenoids and terpenoids

## Abstract

Drought stress is one of major environmental stresses affecting plant growth and yield. Although *Pinus taeda* trees are planted in rainy southern China, local drought sometime occurs and can last several months, further affecting their growth and resin production. In this study, *P. taeda* seedlings were treated with long-term drought (42 d), and then targeted and untargeted metabolomics analysis were carried out to evaluate drought tolerance of *P. taeda*. Targeted metabolomics analysis showed that levels of some sugars, phytohormones, and amino acids significantly increased in the roots and needles of water-stressed (WS) *P. taeda* seedlings, compared with well-watered (WW) pine seedlings. These metabolites included sucrose in pine roots, the phytohormones abscisic acid and sacylic acid in pine needles, the phytohormone gibberellin (GA4) and the two amino acids, glycine and asparagine, in WS pine roots. Compared with WW pine seedlings, the neurotransmitter acetylcholine significantly increased in needles of WS pine seedlings, but significantly reduced in their roots. The neurotransmitters L-glutamine and hydroxytyramine significantly increased in roots and needles of WS pine seedlings, respectively, compared with WW pine seedlings, but the neurotransmitter noradrenaline significantly reduced in needles of WS pine seedlings. Levels of some unsaturated fatty acids significantly reduced in roots or needles of WS pine seedlings, compared with WW pine seedlings, such as linoleic acid, oleic acid, myristelaidic acid, myristoleic acid in WS pine roots, and palmitelaidic acid, erucic acid, and alpha-linolenic acid in WS pine needles. However, three saturated fatty acids significantly increased in WS pine seedlings, i.e., dodecanoic acid in WS pine needles, tricosanoic acid and heptadecanoic acid in WS pine roots. Untargeted metabolomics analysis showed that levels of some metabolites increased in WS pine seedlings, especially sugars, long-chain lipids, flavonoids, and terpenoids. A few of specific metabolites increased greatly, such as androsin, piceatanol, and panaxatriol in roots and needles of WS pine seedlings. Comparing with WW pine seedlings, it was found that the most enriched pathways in WS pine needles included flavone and flavonol biosynthesis, ABC transporters, diterpenoid biosynthesis, plant hormone signal transduction, and flavonoid biosynthesis; in WS pine roots, the most enriched pathways included tryptophan metabolism, caffeine metabolism, sesquiterpenoid and triterpenoid biosynthesis, plant hormone signal transduction, biosynthesis of phenylalanine, tyrosine, and tryptophan. Under long-term drought stress, *P. taeda* seedlings showed their own metabolomics characteristics, and some new metabolites and biosynthesis pathways were found, providing a guideline for breeding drought-tolerant cultivars of *P. taeda*.

## 1 Introduction

Loblolly pine (*Pinus taeda* L.) has garnered great attention in China since its introduction to China, as is cultivated widely in southern China due to its rapid growth, high resin yield, and adaptability to warm and humid climatic conditions (Lilian et al., 2021; Zhang et al., 2021). In recent years, loblolly pine has been highly valued due to its economic and ecological benefits. China produced around 0.6 million tons of resin each year, accounting for about half of the worldwide turpentine trade (McConnell et al., 2021; Yi et al., 2021), most of which was harvested from loblolly pine. However, these benefits are affected by drought stress. Although plantations of loblolly pine distribute in rainy southern China, under the background of global climate change, drought stress often occurs in this region, affecting growth and development of loblolly pine trees and their resin prodcution. For example, severe drought occurred in autumn, winter, and spring in 2009/2010 and 2011/2012 in southwestern China, especially in Yunnan province (Sun et al., 2014). In March-June of 2018, severe drought occurred in Fujian province, located in southeastern China (He et al., 2022). Therefore, climatic change-induced drought is considered a serious threat to forest health due to reduction in water transport, stomatal conductance and net photosynthesis and, consequently, decrease in plant growth and biomass, as well as fire susceptibility and pathogen and insect attacks (Maggard et al., 2016; Brodribb et al., 2020; Liu et al., 2022).

In order to maintain good ecological safety and forest environment, research on drought tolerance of forest trees has been emphasized. Water availability in terrestrial ecosystems has also been identified as the most important single factor in predicting net primary productivity (Lorenz et al., 2006). During sylvicultural activity, drought stress is a major cause of mortality for both planted and naturally-regenerated pine seedlings, especially long-term severe drought (Burns and Honkala, 1990; Kolb and Robberecht, 1996; Mueller et al., 2005; Lorenz et al., 2006). Differentiation in drought tolerance of crop species has long been recognized to reflect their genetic basis (Thabet et al., 2018; Dossa et al., 2019; Mahmood et al., 2019), thus coniferous species also have their genetic basis for differentiation in drought tolerance. Because of this reason, recently substantial effort has been devoted to understanding genetic basis in differential drought tolerance of coniferous species and other forest trees using molecular and genomic tools (Haas et al., 2021; Xiao et al., 2021; Li M. et al., 2022). The loblolly pine genome has been completely sequenced (Neale et al., 2014; Zimin et al., 2014; Zimin et al., 2017). This advancement provided the prerequisite for better understanding the genetic basis of loblolly pine response to drought stress.

Although modern agriculture has considerably reduced the effects of drought on crop growth and yield due to extensive water administration systems, climatic change scenarios that present the possibility of greatly changed rainfall patterns, combined with a rising human population’s demands on water resources, have emphasized the significance of studying plant responses to drought stress (Seleiman et al., 2021). Drought stress provokes a multitude of dramatic alterations in all plant organs at the morphological, biochemical, physiological, and molecular level (Hura et al., 2022), virtually disrupting the link between source and sink organs of plants (Michaletti et al., 2018). The responses of plants to environmental stresses are highly dynamic and complicated, with the goal of developing a new homeostasis under adverse growing conditions. Particularly, drought-responsive mechanisms contain regulation of gene expression (Janiak et al., 2016; Castroverde, 2019), kinase cascades of signaling (Razi and Muneer, 2021), hormone signaling (Jogawat et al., 2021), osmolyte biosynthesis (Ozturk et al., 2021), morphological and anatomical changes in roots and leaves (Jogawat et al., 2021), cell structure modulation (Jogawat et al., 2021), carbohydrate metabolism and allocation (Aaltonen et al., 2017; Kreuzwieser et al., 2021; Razi and Muneer, 2021), stability and activation of water channels (aquaporins) (Yepes-Molina et al., 2020; Chen Q et al., 2021; Patel and Mishra, 2021), fatty acid metabolism (Asakura et al., 2021; Chen et al., 2021), nitrogen assimilation (Das et al., 2017; Miranda-Apodaca et al., 2020), as well as amino acid metabolism (Batista-Silva et al., 2019; Diniz et al., 2020). However, little research was carried out about comprehensive analysis on molecular responses of these alternations to drought stress. Therefore, multiple -omics provide a whole access to understand the comprehensiveness.

Although proteins play important roles in plant responses to drought stress, some small secondary metabolites also shown important functions to enhance drought tolerance, such as the phytohormone ABA (Li et al., 2021; Mathan et al., 2021) and the neurotransmitter melatonin (Sharma and Zheng, 2019; Tiwari et al., 2021; Zeng et al., 2022). Therefore, accurate and simultaneous studies of transcriptomics, proteomics and metabolomics are needed to identify the biochemical and physiological responses of plants to drought stress. It is generally believed that high-performance “omics” approaches enable researchers to examine the biochemical, physiological, and molecular responses of plants under environmental stresses in a more comprehensive way. More specifically, the explanation of the composite regulatory system triggered by plants under environmental stresses is enabled by the integration of such comprehensive techniques (Kosová et al., 2015; Jorge et al., 2016; Michaletti et al., 2018).

In current studies, transcriptomic, proteomic, and metabolomic approaches are mostly used to identify the response of model plants to drought stress (Chmielewska et al., 2016; Michaletti et al., 2018; Lima et al., 2019; Du et al., 2020; Guo et al., 2020; Zhou et al., 2022), but there is a lack of integrated research on metabolites involved in drought tolerance of forest trees. Therefore, the aim of this study was to measure changes in metabolite levels in the roots and needles of loblolly pine seedlings under long-term drought stress, and to identify major metabolic pathways in loblolly pine seedlings related to drought stress. This work lays the foundation for understanding the metabolic mechanisms of drought tolerance in loblolly pine trees, and provides a framework for metabolomics studies in coniferous species.

## 2 Materials and methods

### 2.1 Experimental materials

Seeds of loblolly pine (*Pinus taeda*) came from the Forest Farm of Maple Moutain in Jingdezhen, Jiangxi province, China. These seeds were sterilized using 75% ethanol for 10 min and rinsing 3 times in sterilized water, and then sown in sterilized sand in big plastic pots. After these seeds germinated, the pots were transferred to a growth chamber (16 h light/8 h dark, 350 μmol·m^-2^·s^-1^ PPFD, 80% RH, 25°C). When pine seedlings grew with 4 true needles, they were transplanted into plastic pots (20 cm in diameter and 30 cm in height) containing sterilized cultivation substance (peat:vermiculite = 50:50, pH~7.0), three pine seedlings per pot. These pots were then transferred into a greenhouse with natural sunlight and temperature. Water was provided according to the moisture of the cultivation substance in the pots. When these pine seedlings grew for 7 months, drought treatment was carried out: 30 pots were supplied with 100 ml of water every week, i.e., water-stressed (WS) pine seedlings, and 30 pots were supplied with enough water as control, i.e. well-watered (WW) pine seedlings. The drought treatment continued for 42 d. The pine seedlings were dug out and their roots and needles were rinsed with sterilized water and treated with liquid nitrogen and stored under -80°C for further analysis.

### 2.2 Methods of targeted metabolomic analysis

#### 2.2.1 Amino acids analysis

Metabolite extraction was carried out according to methods introduced by Fuertig et al. (2016) and Virág et al. (2020). Samples were weighed accurately and encased in 2 ml Eppendorf tubes. 600 μL 10% formic acid/methanol-H_2_O (1:1, V/V) and 2 steel beads were added to every tube. The tubes were mixed by well vortex for 30 s. Then samples were ground 90 s under 60 Hz, and then centrifuged for 5 min (12000 rpm under 4°C. 100 μL of the supernatant was added 900 μL of 10% formic acid/methanol-H_2_O (1:1, V/V), and then mixed for 30 s. 100 μL of the diluent was taken out and added 100 ppb isotope internal standard solution. After mixing, the solution was treated with 0.22 μm filter membrane. The filtrate was added into detection bottle.

The LC/MS analysis methods were according to introduction by Liyanaarachchi et al. (2018) and Thiele et al. (2019). A liquid chromatograph (EXion LC, SCIEX) and ZORBAX Eclipse XDB-C18 chromatographic column (4.6 × 150 mm, Agilent company, USA) were used for amino acid analysis. 50 μL of sample solution was added, column temperature: 40°C, moving phase A-10% methanol with 0.1% formic acid; moving phase B-50% methanol with 0.1% formic acid. Gradient elution condition was listed below: 0~6.5 min, 10%~30% B; 6.5~7 min, 30~100% B; 7~18 min, 100% B; 18~18.5 min, 100~10% B; 18.5~21 min, 10% B, 0~8 min, flow speed: 0.3 mL/min; 8.5~21 min. Flow rate: 0.4 mL/min. A mass spectrometer (SCIEX 6500+) was used for the amino acid analysis. Analysis condition was listed below: electrospray ion source: negative and positive ion mode; temperature of ion source: 500°C; voltage of ion source: 5500 V; collision gas: 6 psi; curtain gas: 30 psi; atomization gas and auxiliary gas: 50 psi. Multiple reaction monitoring was used to scan metabolites.

#### 2.2.2 Phytohormone analysis

About 0.1 mg sample was weighed and added 1 ml cool 50% ACN solution. The sample was treated using ultrasonic wave for 3 min under 4°C, and then extracted for 30 min under 4°C. The sample was centrifuged for 10 min (12000 rpm, 4°C). The supernatant was treated using RP-SPE column: 1 mL 100% MeOH and 1 mL de-ionized water was added. 50% ACN solution was used for balance. After sample was loaded, 1 mL 30% ACN was used to rinse column, the components were collected. The collected components were treated to dryness under nitrogen gas flow. 200 μL 30%ACN was added to the dry sample and make it dissolved. The sample was transferred to a sample bottle with insert. HPLC chromatograph (Vanquish, UPLC, Thermo, USA) was used. Column: Waters HSS T3 (50 × 2.1 mm, 1.8 μm). Moving phase **(A)** ultrapure water with 0.1% acetic acid; moving phase **(B)** acetonitrile with 0.1% acetic acid; flow rate: 0.3 ml/min; column temperature: 40°C; sample size: 2 μL; elution gradient: 0 min water/acetonitrile (90:10, V/V), 1 min water/acetonitrile (90:10, V/V), 7 min water/acetonitrile (90:10, V/V), 7.1 min water/acetonitrile (10:90, V/V), 9 min water/acetonitrile (10:90, V/V). A mass spectrometer (Q Exactive, Thermo, USA) was used. Spectrometer parameters were listed below: electrospray ionization source, sheath gas: 40 arb; auxiliary gas: 10 arb;electrospray voltage: -2800 V; temperature: 350°C; temperature of ion transmission pipe: 320°C; scanning mode: single ion detection; scanning way: negative ion.

#### 2.2.3 Neurotransmitter analysis

About 50 mg samples were weighed and mixed with 30 μL 10% formic acid/methanol solution twice under 30 Hz 90 s and then were treated 30 min using ultrasonic wave. The samples were centrifuged for 10 min (13000 rpm, 4°C). The supernatant was taken out and then the centrifugation was repeated once. The supernatant was used for neurotransmitters with lower concentrations in samples. For determination of few neurotransmitters (such as GABA, Gln, Glu, Tyr, Trp), the supernatant was diluted 200 times. AB 4000 triple stage quadrupole mass spectrometry and Waters UPLC liquid chromatograph were used for neurotransmitter analysis. Analysis condition of liquid chromatograph was listed below: ACQUITY UPLC^®^ BEH C18 chromatographic column (2.1×100 mm, 1.7 μm, Waters company, USA); sample size: 5 μL; column temperature: 40°C; moving phase **(A)** 10% methanol with 0.1% formic acid; moving phase **(B)** 50% methanol with 0.1% formic acid. Gradient elution condition: 0~1 min, 20~100% B; 1~7 min, 100% B; 7~7.5 min, 100~20% B; 7.5~11 min, 20% B. Flux rate 0.4 mL/min. Analysis condition of mass spectrometer was listed below: electrospray ion source: negative and positive ion mode; temperature of ion source: 500°C; voltage of ion source: 5000 V; collision gas: 6 psi; curtain gas: 30 psi; atomization gas and auxiliary gas: 50 psi. Multiple reaction monitoring was used to scan metabolites.

#### 2.2.4 Sugar analysis

About 100 mg sample was weighed and loaded in 2.0 mL tube. 700 μl of 80% ethanol was added and mixed for 2 h under 50°C. The sample was diluted with 700 μl H_2_O and was centrifuged for 3 min (10000 rpm). The supernatant was transferred a new centrifuge tube. The supernatant was detected using Thermo ICS5000 ion chromatograph (Dionex, Thermo Scientific, Waltham, USA). An electrochemical detector was used for sugar determination. Ion chromatograph condition was listed below: liquid chromatograph column: CarboPac™ PA1(50 × 4.0mm); moving phase **(A)** H_2_O; moving phase **(B)** 100 mM NaOH; sample size: 5 uL, flow rate: 0.5 ml/min, column temperature: 30°C; gradient elution: 0 min A phase/B phase (95:5, V/V), 9 min A phase/B phase (95:5, V/V), 20 min A phase/B phase (0: 100, V/V), 30 min A phase/B phase (0: 100, V/V), 30.1 min A phase/B phase (95:5, V/V), 40 min A phase/B phase (95:5, V/V), 60 min A phase/B phase (95:5, V/V).

#### 2.2.5 Organic acid analysis

Metabolite extract was carried out according to the method introduced by Selli et al. (2018). Sample was weighed accurately and loaded in a 2 mL EP tube with a steel bean. 500 μL 30% methanol with 0.1% formic acid was added. The sample was ground using a high throughput tissue grinder (60 Hz) for 120 s and then centrifuged for 10 min (12000 rpm, 4°C). 20 μL of supernatant was added to 980 μL 30% methanol with 0.1% formic acid and then mixed for 30 s using a well vortex. The supernatant wad poured into the detective bottle.

Waters UPLC liquid chromatograph and AB4000 mass spectrometer (SCIEX) were used for analysis (Fiori et al., 2018; Pawlak et al., 2019). Chromatograph condition was listed below: chromatograph column: ACQUITY UPLC^®^ BEH C18 column(2.1 × 100 mm, 1.7 μm, Waters company, USA); sample size: 5 μL; column temperature: 40°C; moving phase **(A)** water with 0.1% formic acid; moving phase **(B)** methanol with 0.1% formic acid; gradient elution condition: 0~3 min, 30% B; 3~5 min, 30~50% B; 5~7 min, 50~90% B; 7~9 min, 90% B; 9~13 min, 30% B. Flow rate: 0.4 mL/min. Analysis condition of mass spectrometer was listed below: electrospray ion source: negative ion mode, ion source temperature: 500°C; ion source voltage: -4500 V; collision gas: 6 psi; curtain gas: 30 psi; atomization gas and auxiliary gas: 50 psi. Multiple reaction monitoring was used to scan metabolites.

#### 2.2.6 Fatty acid analysis

The samples (100 mg) were resuspended with liquid nitrogen, then were taken respectively and homogenized with 300 μL of isopropanol/acetonitrile (1:1) which contained mixed internal standards and centrifuged at 12,000 rpm for 10 min. The supernatant was diluted 20 times by Isopropanol/acetonitrile (1:1) which contained mixed internal standards and centrifuged at 12,000 rpm for 10 min. The supernatant was injected into the LC-MS/MS system for analysis. An ultra-high performance liquid chromatography coupled to tandem mass spectrometry (UHPLC-MS/MS) system (ExionLC™ AD UHPLC-QTRAP 6500+, AB SCIEX Corp., Boston, MA, USA) was used to quantify fatty acids in Novogene Co., Ltd. (Beijing, China). Separation was performed on a Waters ACQUITY UPLC BEH C18 column (2.1×100 mm, 1.7 μm) which was maintained at 50°C. The moving phase, consisting of 0.05% formic acid in water (solvent A) and isopropanol/acetonitrile (1:1) (solvent B), was delivered at a flow rate of 0.30 mL/min. The solvent gradient was set as follows: initial 30% B, 1 min; 30-65% B, 2 min; 65-100% B, 11 min; 100% B, 13.5 min; 100-30% B, 14 min; 30% B, 15 min. The mass spectrometer was operated in negative multiple reaction mode (MRM) mode. Parameters were as follows: ionspray voltage (-4500 V), curtain gas (35 psi), ion source temperature (550°C), ion source gas of 1 and 2 (60 psi). Standard curve and limit of quantification (LOQ): LC-MS was used to detect the concentration series of standard solution. The ratio of concentration of standard to internal standard as abscissa, and the ratio of peak area of standard to internal standard as ordinate to investigate the linearity of standard solution. The correlation coefficient (r) > 0.99 of each metabolite was the necessary condition. LOQ were determined by the method of signal-to-noise ratio (S/N), which is comparing the signal measured by the standard solution concentration with the blank matrix. Generally, when the S/N = 10:1, the corresponding concentration is the LOQ (Chinese Pharmacopoeia Commission, 2015; Chevolleau et al., 2018).

### 2.3 Methods of untargeted metabolomic analysis

#### 2.3.1 Metabolite extraction

Tissues (100 mg) were individually grounded with liquid nitrogen and the homogenate was resuspended with prechilled 80% methanol by well vortex. The samples were incubated on ice for 5 min and then were centrifuged at 15,000 *g*, 4°C for 20 min. Some of supernatant was diluted to final concentration containing 53% methanol by LC-MS grade water. The samples were subsequently transferred to a fresh Eppendorf tube and then were centrifuged at 15000 g, 4°C for 20 min. Finally, the supernatant was injected into the LC-MS/MS system analysis (Want et al., 2013).

#### 2.3.2 UHPLC-MS/MS analysis

UHPLC-MS/MS analyses were performed using a Vanquish UHPLC system (ThermoFisher, Germany) coupled with an Orbitrap Q ExactiveTM HF mass spectrometer (Thermo Fisher, Germany) in Novogene Co., Ltd. (Beijing, China). Samples were injected into a Hypesil Gold column (100×2.1 mm, 1.9 μm) using a 17-min linear gradient at a flow rate of 0.2 mL/min. The eluents for the positive polarity mode were eluent A (0.1% FA in water) and eluent B (methanol). The eluents for the negative polarity mode were eluent A (5 mM ammonium acetate, pH 9.0) and eluent B (methanol). The solvent gradient was set as follows: 2% B, 1.5 min; 2-85% B, 3 min; 100% B, 10 min; 100-2% B, 10.1 min; 2% B, 12 min. Q ExactiveTM HF mass spectrometer was operated in positive/negative polarity mode with spray voltage of 3.5 kV, capillary temperature of 320°C, sheath gas flow rate of 35 psi and aux gas flow rate of 10 L/min, S-lens RF level of 60, aux gas heater temperature of 350°C.

#### 2.3.3 Data processing and metabolite identification

The raw data files generated by UHPLC-MS/MS were processed using the Compound Discoverer 3.1 (CD3.1, ThermoFisher) to perform peak alignment, peak picking, and quantitation for each metabolite (Schymanski et al., 2014). The main parameters were set as follows: retention time tolerance, 0.2 minutes; actual mass tolerance, 5 ppm; signal intensity tolerance, 30%; signal/noise ratio, 3; and minimum intensity, *etc*. After that, peak intensities were normalized to the total spectral intensity. The normalized data was used to predict the molecular formula based on additive ions, molecular ion peaks and fragment ions. Then, peaks were matched with the mzCloud (https://www.mzcloud.org/), mzVault and MassList database to obtain the accurate qualitative and relative quantitative results. Statistical analyses were performed using the statistical software R (R version R-3.4.3), Python (Python 2.7.6 version) and CentOS (CentOS release 6.6), When data were not normally distributed, normal transformations were attempted using of area normalization method.

#### 2.3.4 Data analysis

These metabolites were annotated using the KEGG database (https://www.genome.jp/kegg/pathway.html), HMDB database (https://hmdb.ca/metabolites) and LIPIDMaps database (http://www.lipidmaps.org/). Principal components analysis (PCA) and Partial Least Squares-Discriminant Analysis (PLS-DA) were performed at metaX (a flexible and comprehensive software for processing metabolomics data) (Wen et al., 2017). We applied univariate analysis (t-test) to calculate the statistical significance (*p*-value).The metabolites with VIP > 1 and *p*-value < 0.05 and fold change ≥ 2 or FC ≤ 0.5 were considered to be differential metabolites. Volcano plots were used to filter metabolites of interest which based on log2 (FoldChange) and -log10 (*p*-value) of metabolites by ggplot2 in R language. For clustering heat maps, the data were normalized using z-scores of the intensity areas of differential metabolites and were plotted by Pheatmap package in R language. The correlation between differential metabolites were analyzed by cor () in R language (method=pearson). Statistically significant of correlation between differential metabolites were calculated by cor.mtest() in R language. *p*-value < 0.05 was considered as statistical significance and correlation plots were plotted by corrplot package in R language. The functions of these metabolites and metabolic pathways were studied using the KEGG database. The metabolic pathways enrichment of differential metabolites was performed, when ratio were satisfied by x/n > y/N, metabolic pathway were considered as enrichment, when *p*-value of metabolic pathway < 0.05, metabolic pathway were considered as statistically significant enrichment. Data of targeted metabolomics were analyzed using SPSS software (17 v.), and the test level was set to *p* = 0.05. Multiple comparisons were carried out among the four sample groups, i.e., roots and needles of WW and WS pine seedlings.

## 3 Results

### 3.1 Targeted metabolics analysis

#### 3.1.1 Changes in concentrations of sugars in *Pinus taeda* seedlings under drought stress

**Figure 1:**
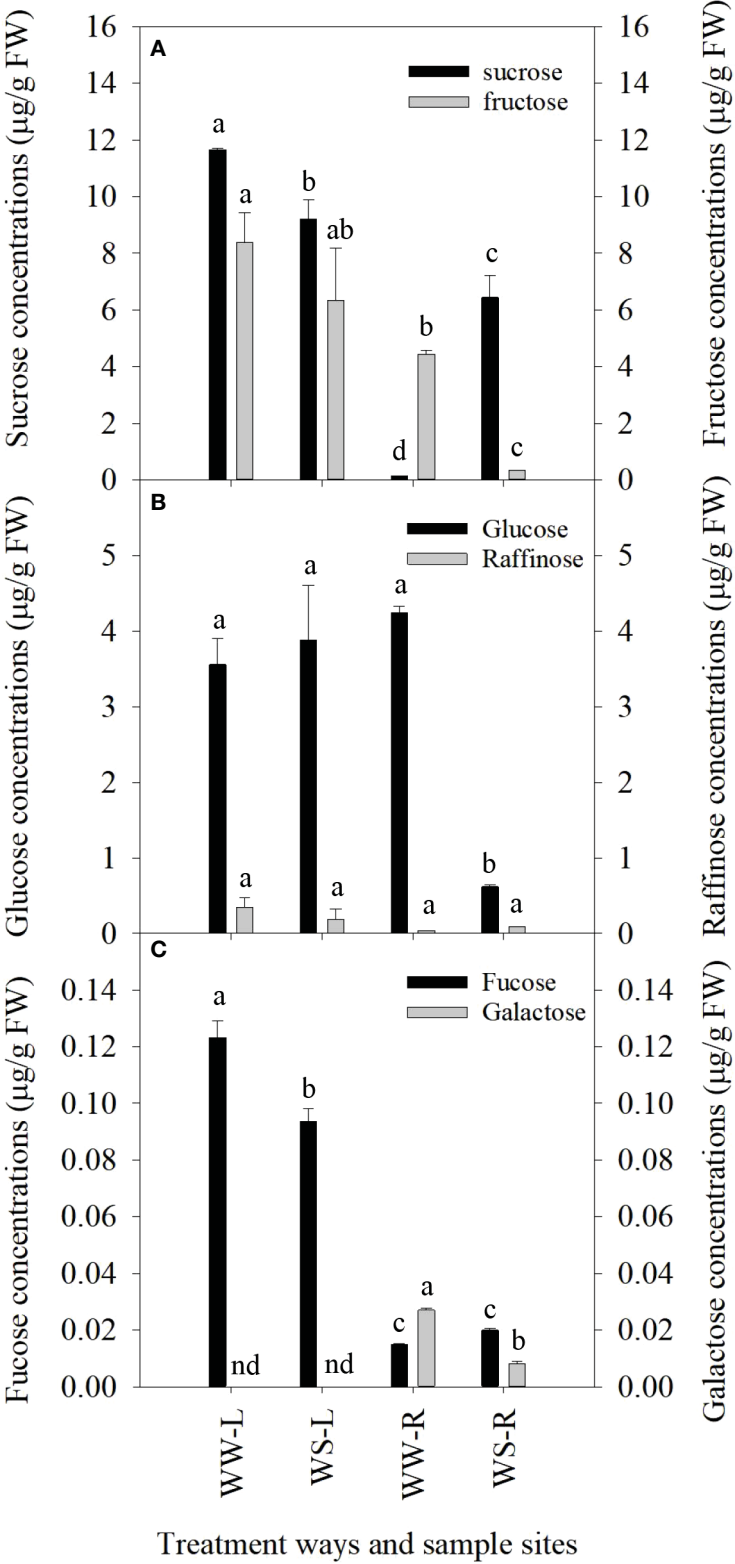
Concentrations of sugars in leaves (L) and roots (R) of *P. taeda* seedlings under well-watered (WW) and water-stressed (WS) conditions. **(A)** concentrations of sucrose and fructose; **(B)** concentrations of glucose and raffinose; **(C)** concentrations of fucose and galactose. nd=not determined in sample. For the same sugar, the different low case letters above the bars stand for the significant differences between them (*p* < 0.05).

Under drought stress, great changes occurred in concentrations of sugars in needles and roots of *P. taeda* seedlings. Sucrose levels in needles of WS pine seedlings were significantly lower than those in needles of WW pine seedlings (*p* < 0.05, **Figure 1A**), but the status of sucrose levels in roots were just reversed, i.e., sucrose levels in roots of WS pine seedlings were significantly higher than those in roots of WW pine seedlings (*p* < 0.05, **Figure 1A**). Fructose concentrations in needles did not show significant changes between WW and WS pine seedlings (*p* > 0.05, **Figure 1A**), but fructose concentrations in roots of WS pine seedlings were significantly lower than those in roots of WW pine seedlings (*p* < 0.05, **Figure 1A**). Glucose concentrations in needles did not show significant difference between WS and WW pine seedlings (*p* > 0.05, **Figure 1B**), but glucose concentrations in roots of WS pine seedlings were significantly lower than those in roots of WW pine seedlings (*p* < 0.05, **Figure 1B**). Raffinose concentrations showed no differences among needles and roots of WW and WS pine seedlings (*p* > 0.05, **Figure 1B**). Fucose concentrations in needles of WS pine seedlings were significantly lower than those in needles of WW pine seedlings (*p* < 0.05, **Figure 1C**), but no difference between their roots (**Figure 1C**). Galactose was not detected in needles of WW and WS pine seedlings, and galactose levels in roots of WS pine seedlings were significantly lower than those in roots of WW pine seedlings (*p* < 0.05, **Figure 1C**).

#### 3.1.2 Changes in concentrations of amino acids in *Pinus taeada* seedlings under drought stress

**Figure 2:**
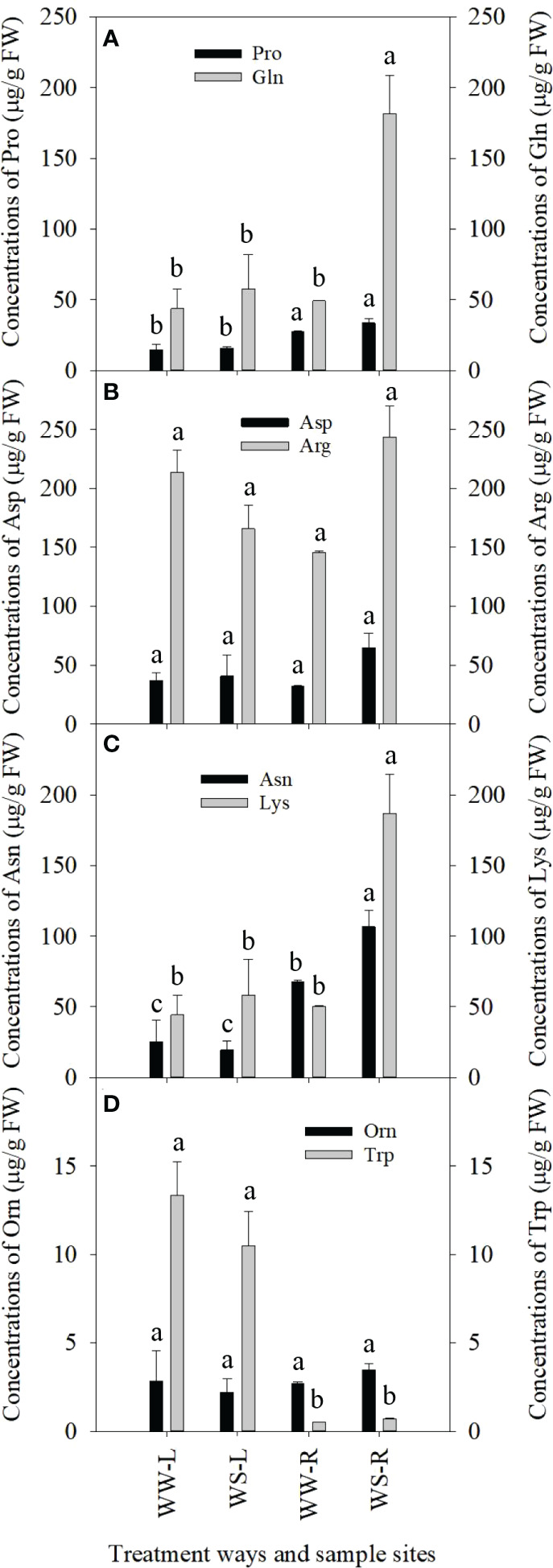
Concentrations of amino acids in needles (L) and roots (R) of *P. taeada* seedlings under well-watered (WW) and water-stressed (WS) conditions. **(A)** concentrations of the two amino acids, Pro and Gln, in needles and roots; **(B)** concentrations of the two amino acids, Asp and Arg, in needles and roots; **(C)** concentrations of the two amino acids, Asn and Lys, in needles and roots; **(D)** concentrations of the two amino acids, Orn and Trp, in needles and roots. For the same amino acid, the different low case letters above the bars stand for the significant differences between them (*p* < 0.05).

Great changes in concentrations of some amino acids occurred in needles and roots of *P. taeda* seedlings under drought stress. Pro levels in needles of WW and WS pine seedlings showed no statistical difference (*p* > 0.05, **Figure 2A**), and Pro levels were significantly higher in roots than those in needles of WS pine seedlings (*p* < 0.05, **Figure 2A**), but showed no statistical difference, compared with those in roots of WW pine seedlings (*p* > 0.05, **Figure 2A**). Gln levels in roots of WS pine seedlings were significantly higher than those in roots of WW pine seedlings (*p* < 0.05, **Figure 2A**). Concentrations of Asp and Arg showed no statistical differences among the needles and roots of WW and WS pine seedlings (*p* > 0.05, **Figure 2B**). Levels of Asn and Lys in needles of WW and WS pine seedlings showed no statistical difference, but levels of Asn and Lys in WS pine roots were significantly higher than those in WW pine roots (*p* < 0.05, **Figure 2C**). Orn and Trp levels in needles and roots of WW and WS pine seedlings showed no statistical differences (*p* > 0.05, **Figure 2D**).

#### 3.1.3 Changes in concentrations of organic acids in *Pinus taeda* seedlings under drought stress

**Figure 3:**
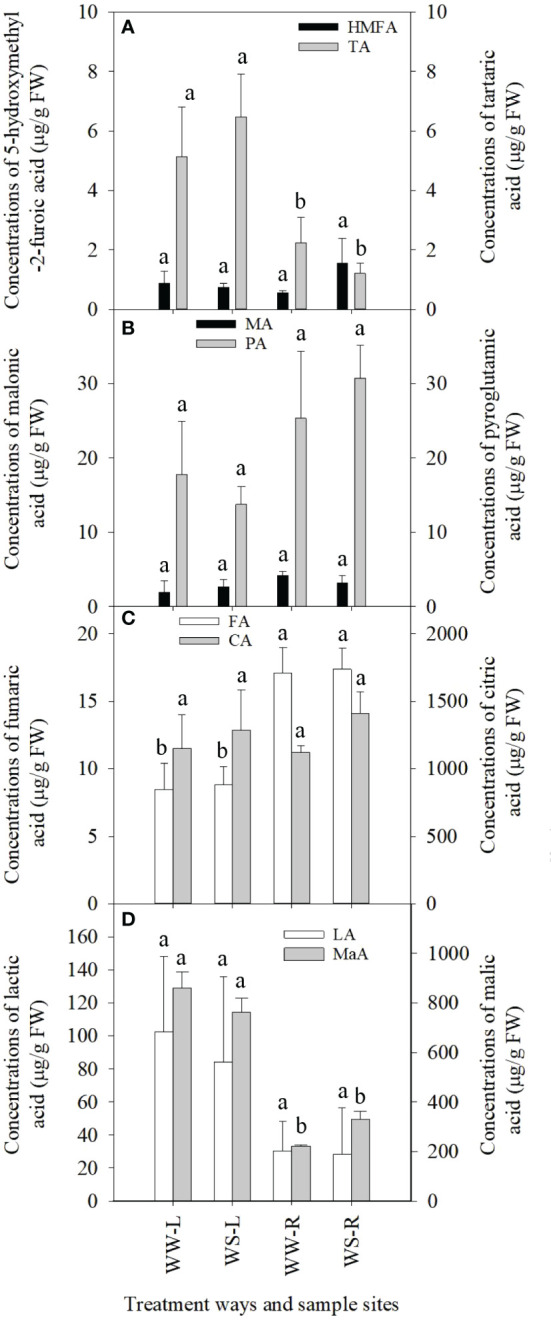
Concentrations of organic acids in needles (L) and roots (R) of *P. taeda* seedlings under well-watered (WW) and water-stressed (WS) conditions. **(A)** 5-hydroxymethyl-2-furoic acid (HMFA) and tartaric acid (TA); **(B)** malonic acid (MA) and pyroglutamic acid (PA); **(C)** fumaric acid (FA) and citric acid (CA); **(D)** lactic acid (LA) and malic acid (MaA). For the same organic acid, the different low case letters above the bars stand for the significant differences between them (*p* < 0.05).

Concentrations of 5-hydroxymethyl-2-furoic acid (HMFA) showed no statistical differences between needles and roots of WW and WS pine seedlings (*p* > 0.05, **Figure 3A**), but HMFA concentrations in roots of WS pine seedlings were significantly lower than those in their needles (*p* < 0.05, **Figure 3A**). Levels of tartaric acid showed no statistical differences among the needles and roots of WW and WS pine seedlings (*p* > 0.05, **Figure 3A**). The similar situation occurred in concentrations of malonic acid (MA) and pyroglutamic acid (PA) (**Figure 3B**). Concentrations of fumaric acid (FA) in needles or roots showed no statistical difference between WW and WS pine seedlings (*p* > 0.05, **Figure 3C**), but FA concentrations in roots of WW and WS pine seedlings were significantly higher than those in their needles, respectively (*p* < 0.05, **Figure 3C**). Concentrations of citric acid (CA) and lactic acid (LA) showed no statistical differences among the four samples (**Figures 3C, D**). Concentrations of malic acid (MaA) in roots of WW and WS pine seedlings were significantly lower than those in their needles, respectively (*p* < 0.05, **Figure 3D**), but no statistical differences in the two organic acids occurred in needles or roots between WW and WS pine seedlings (**Figure 3D**). In addition, concentrations of glucuronic acid and pyridoxine in needles and roots of pine seedlings under drought stress significantly reduced (*p* < 0.05, unshown data), compared with WW condition, respectively.

#### 3.1.4 Changes in concentrations of phytohormones in *Pinus taeda* seedlings under drought stress

**Figure 4:**
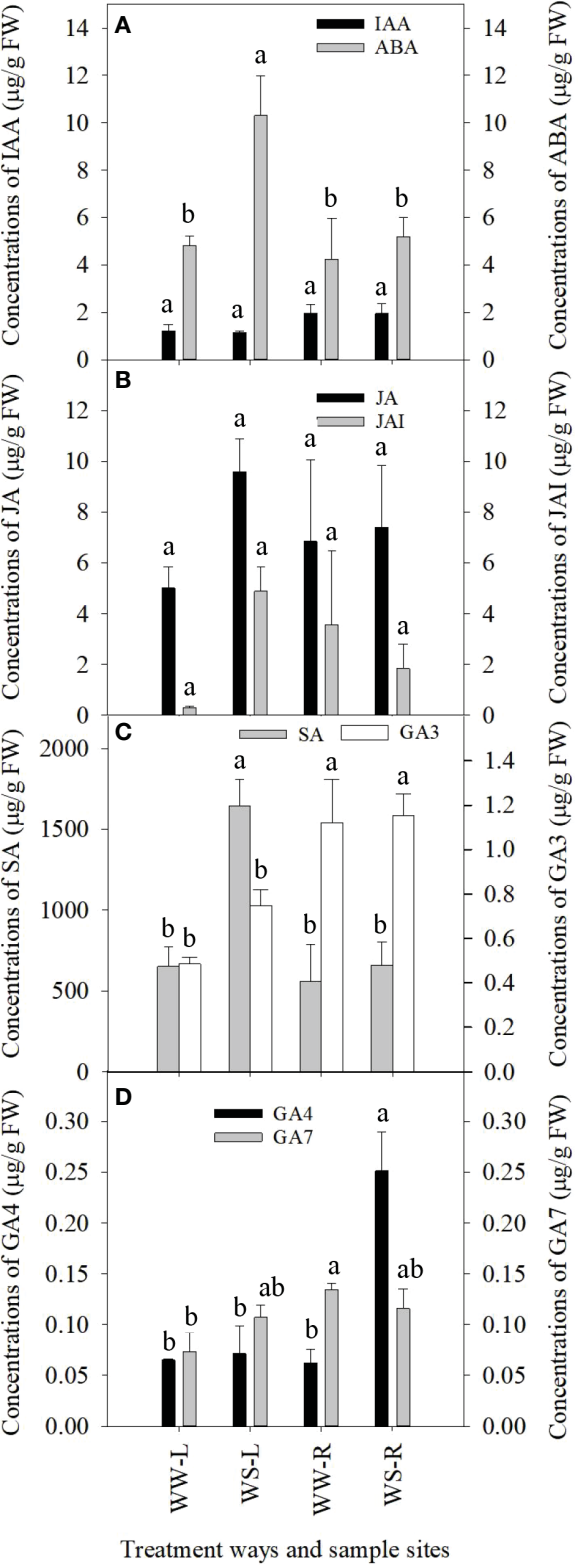
Concentrations of phytohormones in needles (L) and roots (R) of *P. taeda* seedlings under well-watered (WW) and water-stressed (WS) condition. **(A)** concentrations of IAA and ABA; **(B)** concentrations of JA and JAI; **(C)** concentrations of SA and GA3; **(D)** concentrations of GA4 and GA7. For the same phytohormone, the different low case letters above the bars stand for the significant differences between them (*p* < 0.05).

Phytohormones play important roles in plant response to drought stress, thus concentrations of some phytohormones were determined in needles and roots of WW and WS pine seedlings (**Figure 4**). IAA concentrations showed no statistical differences among all the four samples (**Figure 4A**). ABA concentrations in WS pine needles were significantly higher than those in WW pine needles (*p* < 0.05, **Figure 4A**), but ABA levels in roots showed no statistical difference between WW and WS pine seedlings (**Figure 4A**). Concentrations of jasmonic acid (JA) and jasmonic acid-isoleucine (JAI) showed no statistical differences among needles and roots of WW and WS pine seedlings (**Figure 4B**). Salicylic acid (SA) concentrations in needles of WS pine seedlings were significantly higher than those in needles of WW pine seedlings (*p* < 0.05, **Figure 4C**), but no statistical difference in SA concentrations occurred in roots of WW and WS pine seedlings (**Figure 4C**). GA3 concentrations in roots were significantly higher than those in needles of WW and WS pine seedlings (*p* < 0.05, **Figure 4C**), but no statistical differences occurred in needles or roots between WW and WS pine seedlings (**Figure 4C**). No statistical differences occurred in GA4 concentrations in needles of WW and WS pine seedlings (**Figure 4D**), but GA4 concentrations in roots of WS pine seedlings were significantly higher than those in roots of WW pine seedlings (*p* < 0.05, **Figure 4D**). No statistical differences in GA7 concentrations occurred in needles or roots of WS pine seedlings, compared with those in WW pine seedlings, respectively (**Figure 4D**).

#### 3.1.5 Changes in concentrations of neurotransmitters in *Pinus taeda* seedlings under drought stress

Plant neurotransmitters also play important roles in plant growth and development and responses to environmental stresses, especially drought stress. Concentrations of 4-aminobutyric acid in WS pine needles were lower than those in WW pine needles, but no statistical difference occurred (*p* > 0.05, **Table 1**), and the similar situation also occurred in roots of WW and WS pine seedlings (**Table 1**). No statistical differences in concentrations of histamine and tyramine occurred in needles and roots of WW and WS pine seedlings (**Table 1**). Concentrations of acetylcholine in WS pine needles were significantly higher than those in WW pine needles (*p* < 0.05, **Table 1**), but acetylcholine concentrations in roots of WS pine seedlings were significantly lower than those in WW pine seedlings (*p* < 0.05, **Table 1**). Concentrations of L-glutamine in needles of WW and WS pine seedlings showed no statistical difference (**Table 1**), but concentrations of L-glutamine in WS pine roots were significantly higher than those in WW pine roots (*p* < 0.05, **Table 1**). Levels of L-glutamic acid showed no statistical difference in needles or roots of WS pine seedlings, compared with those in needles or roots of WW pine seedlings, respectively (**Table 1**), but levels of L-glutamic acid in WS pine roots were significantly higher than those in their needles (*p* < 0.05, **Table 1**). Hydroxytyramine concentrations in WS pine needles were significantly higher than those in WW pine needles (*p* < 0.05, **Table 1**), but no statistical difference occurred in roots of WW and WS pine seedlings (*p* > 0.05, **Table 1**). No differences in L-histidine concentrations occurred in needles and roots of WW and WS pine seedlings (**Table 1**). Noradrenaline concentrations in WS pine needles were significantly lower than those in WW pine needles (*p* < 0.05, **Table 1**), but no statistical difference in noradrenaline concentrations occurred in roots of WW and WS pine seedlings (**Table 1**). Serotonin, adrenaline, xanthurenic acid, DL-kynurenine, and vanillymandelic acid were not determined in roots of WW and WS pine seedlings, and no statistical differences in their concentrations occurred between needles of WW and WS pine seedlings (**Table 1**). L-tyrosine concentrations show no statistical differences among needles and roots of WW and WS pine seedlings (**Table 1**). Concentration of kynurenic acid in needles or roots showed no statistical differences between WW and WS pine seedlings, respectively (**Table 1**). Concentrations of levodopa in roots were significantly higher than those in needles of WW and WS pine seedlings, respectively (*p* < 0.05, **Table 1**), but levodopa concentrations showed no statistical difference in needles or roots of WW and WS pine seedlings, respectively (**Table 1**). The situation about L-tryptophan concentrations was just opposite to that of levodopa (**Table 1**). Melatonin was not determined in needles of WW and WS pine seedlings, and melatonin concentrations showed no statistical difference between roots of WW and WS pine seedlings (**Table 1**).

**Table 1:** Concentrations of neurotransmitters in leaves (L) and roots (R) of *P. taeda* seedlings under well-watered (WW) and water-stressed (WS) conditions.

Neurotransmitters determined	Treatment ways and sample sites (μg/g FW)			
	WW-L	WS-L	WW-R	WS-R
4-aminobutyric acid	37.4955 ± 7.3055ab	17.8785 ± 3.2485b	55.1240 ± 5.104941a	42.9950 ± 4.1090ab
histamine	0.0240 ± 0.0072a	0.0243 ± 0.0012a	0.02433 ± 0.0007a	0.0297 ± 0.0009a
tyramine	0.0087 ± 0.0063a	0.0547 ± 0.0403a	0.0600 ± 0.0006a	0.0600 ± 0.0084a
acetylcholine	0.0850 ± 0.0620c	0.2460 ± 0.02500b	0.4797 ± 0.0412a	0.3043 ± 0.0291b
L-glutamine	142.4405 ± 7.8965b	125.9545 ± 3.0665b	29.93833 ± 2.71357c	241.5407 ± 3.38523a
L-glutamic acid	137.6510 ± 4.6980b	173.6890 ± 3.8909b	283.4957 ± 5.1912a	346.5337 ± 9.5964a
hydroxytyramine	0.1400 ± 0.0433b	0.3477 ± 0.1092a	0.0777 ± 0.0009b	0.1227 ± 0.0150b
L-histidine	2.9977 ± 0.9932a	3.1627 ± 0.3392a	1.3850 ± 0.0276a	1.8730 ± 0.0653a
noradrenaline	22.6815 ± 0.2675a	12.0277 ± 3.3743b	4.8177 ± 0.1711c	4.6537 ± 0.3468c
serotonin	0.0150 ± 0.0093a	0.0197 ± 0.0030a	nd	nd
L-tyrosine	7.9373 ± 4.0388a	3.4217 ± 0.7135a	3.8870 ± 0.3950a	3.900 ± 0.3295a
Adrenaline	0.0434 ± 0.0099a	0.0380 ± 0.0041a	nd	nd
Kynurenic acid	0.0093 ± 0.0047a	0.0047 ± 0.0018ab	0.0003 ± 0.0000b	0.0007 ± 0.0003b
levodopa	0.0540 ± 0.0093b	0.0307 ± 0.0070b	0.4427 ± 0.0070a	0.4427 ± 0.0665a
L-tryptophan	43.9020 ± 12.1440a	47.0855 ± 18.5515a	3.66230.3897b	4.9403 ± 1.5273b
xanthurenic acid	70.2590 ± 6.3627a	75.07433 ± 9.2933a	nd	nd
DL-kynurenine	0.2263 ± 0.1204a	0.3233 ± 0.060a	nd	nd
vanillymandelic acid	1.7335 ± 0.4045a	2.5145 ± 0.7415a	nd	nd
melatonin	nd	nd	0.0137 ± 0.0003a	0.0140 ± 0.0010a

nd, not determined. Different lowercases for the same physiological parameters indicate significant differences.

#### 3.1.6 Changes in concentrations of fatty acids in *Pinus taeda* seedlings under drought stress

Changes in compositions of fatty acids often occur due to environmental stresses. Concentrations of fatty acids in needles and roots of WW and WS pine seedlings were determined (**Table 2**). Few saturated and unsaturated fatty acids did not show significant level changes in needles or roots between WW and WS pine seedlings (*p* > 0.05, **Table 2**), such as hendecanoic acid (C11:0), tridecanoic acid (C13:0), myristelaidic acid (C14:1n-5), tetracosanoic acid (C24:0), linoelaidic acid (C18:2n-6,9). Levels of some unsaturated fatty acids significantly reduced in roots or needles of WS pine seedlings, compared with those in WW pine seedlings (*p* < 0.05, **Table 2**), such as elaidic acid (C18:1n-10), gamma-linolenic acid (C20:3n-6,9,12), *cis*-11-eicosenoic acid (C20:1n-9), alpha-linolenic acid (C18:3n-3,6,9), *cis*-11,14-eicosadienoic acid (C20:2n-6,9), erucic acid (C22:1n-9), *cis*-13,16-docosadienoic acid (C22:2n-6,9), *tran*s-vaccenic acid (C18:1n-7), linoleic acid (C18:2n-6,9), nervonic acid (C24:1n-9), *cis*-vaccenic acid (C18:1n-7), oleic acid (C18:1n-9), *trans*-10-heptadecenoic acid (C17:1n-7), *cis*-10-heptadecenoic acid (C17:1n-7), palmitelaidic acid (C16:1n-7), palmitoleic acid (C16:1n-7), *trans*-10-pentadecenoic acid (C15:1n-5), *cis*-10-pentadecenoic acid (C15:1n-5), myristoleic acid (C14:1n-5) in roots; palmitelaidic acid (C16:1n-7), erucic acid (C22:1n-9), alpha-linolenic acid (C18:3n-3,6,9) in needles. Gamma-linolenic acid was not determined in WW pine needles, but in WS pine needles; and its levels in WS pine roots were significantly lower than those in WW pine roots (**Table 2**). Astonishingly, compared with WW pine seedlings, three saturated fatty acids significantly increased in WS pine seedlings, i.e., dodecanoic acid (C12:0) in needles, tricosanoic acid (C23:0) and heptadecanoic acid (C17:0) in roots (*p* < 0.05, **Table 2**).

**Table 2:** Changes in fatty acids in roots and needles of WW and WS pine seedlings.

Fatty acids detected	Treatment ways and sample sites (μg/g FW)			
	WW-L	WS-L	WW-R	WS-R
Hendecanoic acid	314.66 ± 120.49ab	487.81 ± 69.56a	89.21 ± 6.34b	27.89 ± 17.71b
Dodecanoic acid	2862.35 ± 183.88b	3837.71 ± 111.75a	3960.13 ± 97.47a	3859.43 ± 60.24a
Tridecanoic acid	524.52 ± 29.19a	551.34 ± 44.82a	318.28 ± 5.91b	338.59 ± 14.05b
Tetradecanoic acid	1962.58 ± 48.21a	1803.38 ± 31.30327b	1699.46 ± 59.54b	767.27 ± 30.57c
Myristoleic acid	27.86 ± 2.51ab	39.26 ± 14.42ab	45.87 ± 1.37a	10.29 ± 0.12b
Myristelaidic acid	100.24 ± 3.50a	123.84 ± 37.01a	154.60 ± 17.02a	98.75 ± 7.07a
Pentadecanoic acid	938.97 ± 37.37c	612.06 ± 5.15c	15424.23 ± 107.96a	11545.74 ± 361.47b
*cis*-10-Pentadecenoic acid	27.17 ± 2.04c	13.37 ± 1.62c	1173.61 ± 72.26a	887.98 ± 120.69b
*trans*-10-Pentadecenoic acid	15.12 ± 5.30c	21.36 ± 3.15c	175.14 ± 10.86a	64.00 ± 2.79b
Hexadecanoic acid	14205.14 ± 529.26c	18327.18 ± 306.24bc	29726.44 ± 2649.84a	21797.17 ± 1117.47b
Palmitoleic acid	431.00 ± 120.08c	428.45 ± 127.36c	15521.51 ± 459.59a	8191.67 ± 148.08b
Palmitelaidic acid	928.00 ± 18.47c	744.84 ± 54.80d	6070.39 ± 56.35a	3131.41 ± 66.63b
Heptadecanoic acid	866.68 ± 18.56b	652.10 ± 15.14c	792.21 ± 37.72b	998.02 ± 15.70a
*cis*-10-Heptadecenoic acid	1929.29 ± 77.51c	1272.01 ± 116.98c	11795.22 ± 498.14a	8556.85 ± 510.76b
*trans*-10-Heptadecenoic acid	346.01 ± 28.10c	122.04 ± 12.47c	11491.97 ± 510.88a	4306.78 ± 192.95b
Oleic acid	3550.43 ± 237.20c	4048.19 ± 107.72c	15939.42 ± 688.10a	13234.24 ± 172.57b
*cis*-Vaccenic acid	3111.10 ± 185.87c	3746.72 ± 125.58c	15153.28 ± 592.00a	11852.87 ± 235.33b
Elaidic acid	849.18 ± 32.06b	799.59 ± 59.60b	1772.57 ± 74.33a	785.60 ± 34.18b
Arachidic acid	1902.72 ± 95.66c	1787.19 ± 385.70c	12437.19 ± 1151.27a	9150.42 ± 197.86b
gamma-Linolenic acid	N.D.	431.46 ± 54.16c	2410.87 ± 110.79a	925.88 ± 50.61b
*cis*-11-Eicosenoic acid	1917.13 ± 20.71b	1646.72 ± 140.67b	2379.12 ± 137.52a	1195.31 ± 24.74c
alpha-Linolenic acid	38117.23 ± 948.47a	28789.38 ± 321.82b	7491.62 ± 354.52c	5149.44 ± 66.69d
Heneicosanoic acid	5022.86 ± 131.02bc	4347.61 ± 468.23c	7137.18 ± 109.31a	5928.13 ± 301.74b
*cis*-11,14-Eicosadienoic acid	3957.46 ± 101.05a	4392.56 ± 429.13a	1946.12 ± 53.10b	667.06 ± 27.65c
Docosanoic acid	4995.50 ± 396.54b	3608.82 ± 373.27c	14060.54 ± 271.14a	13229.26 ± 333.13b
Erucic acid	341.53 ± 46.53ab	131.92 ± 19.73c	444.36 ± 33.08a	313.39 ± 20.36b
Tricosanoic acid	1401.62 ± 43.30c	1623.82 ± 147.39c	2593.76 ± 190.17b	3172.68 ± 73.24a
*cis*-13,16-Docosadienoic acid	174.16 ± 0.70ab	123.89 ± 19.72bc	223.26 ± 36.79a	92.0345 ± 15.22c
trans-Vaccenic acid	802.89 ± 10.87b	734.71 ± 67.64b	1724.33 ± 76.83a	718.57 ± 43.27b
Linoleic acid	28668.41 ± 509.75c	29830.99 ± 882.18c	66436.76 ± 1231.70a	44188.78 ± 2038.21b
Linoelaidic acid	1124.83 ± 138.47b	1121.83 ± 343.89b	3080.31 ± 254.61a	2586.22 ± 99.378a
Tetracosanoic acid	10668.56 ± 439.94b	10589.24 ± 885.52b	44924.82 ± 442.74a	41705.67 ± 2744.47a
Nervonic acid	66.70 ± 2.03c	59.72 ± 6.20c	258.01 ± 28.39a	189.83 ± 4.38b

The results are shown as mean ± SD (n=3) (ng/g FW). For every fatty acid, data with the same lowercases are not significantly different (*p* > 0.05, LSD). In this table, N.D. represents not determined. WW-L and WW-R represent needles and roots of well-watered pine seedlings; WS-L and WS-R represent needles and roots of water-stress pine seedlings.

### 3.2 Untargeted metabolome analysis

#### 3.2.1 Quality control, screening and identification of differential compounds in different samples

**Figure 5:**
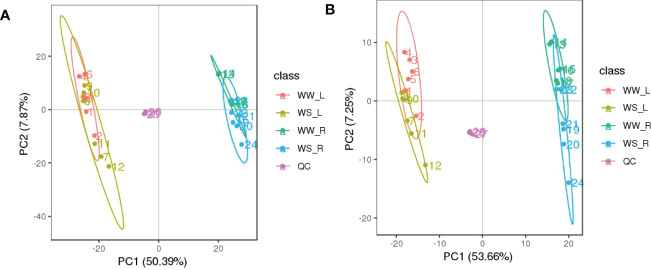
PCA score plots based on the UHPLC-Q-TOF/MS data of needles (L) and roots (R) of *P. taeda* seedlings under well-watered (WW) and water-stressed (WS) condition, based on positive **(A)** and negative **(B)** ion mode.

As described in M & M, metabolites in all the four treatment groups were identified and characterized as many as possible, and then their relatively quantitative levels were analyzed (**Table S1**). Quality control (QC) of untargeted metabolomics analysis was evaluated. Before the subsequent analyses, all the data of differential metabolites were subjected to a data integrity check, and no missing values were detected. The distribution of metabolic profiles for the tested samples and QC samples in PCA is shown in **Figure 5**. All the tested samples and QC samples clustered together, suggesting that the method had good reproducibility overall.

**Figure 6:**
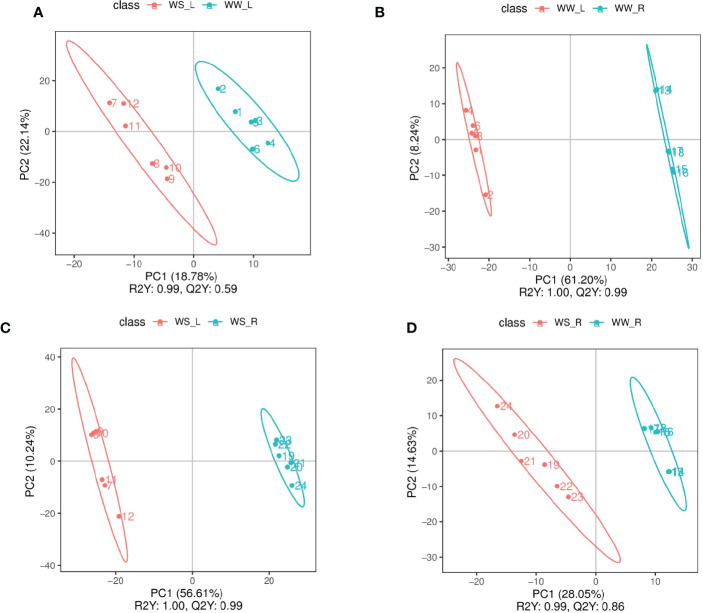
PLS-DA scores of experimental groups under positive ion mode. **(A)** needles (red) vs. roots (blue) of WW seedlings; **(B)** needles of WS (red) vs.WW (blue) pine seedlings; **(C)** needles (red) vs. roots (blue) of WS pine seedlings; **(D)** roots of WS (red) vs. WW (blue) pine seedlings. WW, well-watered; WS, water-stressed.

PLS-DA and PCA were used to screen and identify differential metabolites (**Figures 6**, **S1**, **S2**). Results from PLS-DA and PCA showed that metabolite differentiation and variations between the four sample groups were satisfactory and identified metabolites were suitable. According to results from PLS-DA under positive mode, 281 and 255 metabolites were up- and down-regulated, respectively, comparing between needles and roots of WW pine seedlings (i.e., WW_L.vs.WW_R_pos); 38 and 63 metabolites were up- and down-regulated, respectively, comparing between the needles of WS and WW pine seedlings (i.e., WS_L.vs.WW_L_pos); 240 and 290 metabolites were up- and down-regulated, respectively, comparing needles and roots of WS pine seedlings (i.e., WS_L.vs.WS_R_pos); 103 and 74 metabolites were up- and down-regulated, respectively, comparing roots of WS and WW pine seedlings (i.e., WS_R.vs.WW_R_pos) (**Table 3**). Under negative ion mode, 27 and 17 metabolites were up- and down-regulated, respectively, compared with the needles of WS and WW pine seedlings (i.e., WS_L.vs.WW_L_neg, **Table 3**); 49 and 28 metabolites were up- and down-regulated, respectively, comparing with the roots of WS and WW pine seedlings (i.e., WS_R.vs.WW_R_neg, **Table 3**). Thus, all together, 65 and 80 metabolites were up- and down-regulated in WS pine needles, respectively, comparing with those in the needles of WW pine seedlings; 152 and 102 metabolites were up- and down-regulated in WS pine roots, compared with those in the roots of WW pine seedlings. The differential metabolites were annotated and some metabolites showed great fold changes (**Tables S2**, **S3**).

**Table 3:** Summary of selective results of differential metabolites.

Compared Samples^1^	Num. of Total Ident.^2^	Num. of Total Sig.^3^	Num. of Sig. UP^4^	Num. of Sig. Down^5^
WW_L. vs. WW_R_pos	1059	536	281	255
WS_L. vs. WW_L_pos	1059	101	38	63
WS_L vs. WS_R_pos	1059	530	240	290
WS_R. vs. WW _R_pos	1059	177	103	74
WW_L. vs. WW_R_neg	526	282	117	165
WS_L. vs. WW_L_neg	526	44	27	17
WS_L. vs. WS_R_neg	526	286	115	171
WS_R. vs.WW_R_neg	526	77	49	28

(1) WW-L and WW-R represent needles and roots of well-watered pine seedlings; WS-L and WS-R represent needles and roots of water-stress pine seedlings. (2) Total number of identified metabolites; (3) Total number of metabolites with significant differences; (4) Total number of metabolites significantly up-regulated; (5) Total number of metabolites significantly down-regulated.

#### 3.2.2 Venn diagram analysis of differential metabolites

**Figure 7:**
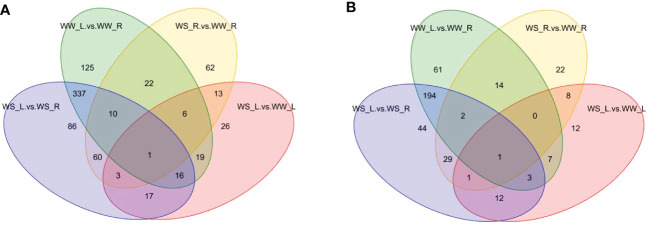
Venn diagram of differential metabolites under positive **(A)** and negative **(B)** ion modes. WW-L and WW-R represent needles and roots of well-watered pine seedlings, respectively; WS-L and WS-R represent needles and roots of water-stress pine seedlings, respectively.

Venn diagrams showed that 61 and 125 unique differential metabolites occurred between needles and roots of WW pine seedlings under positive and negative ion mode, respectively; 44 and 86 unique differential metabolites occurred between needles and roots of WS pine seedlings under positive and negative ion mode, respectively. 22 and 62 unique differential metabolites occurred between roots of WS and WW pine seedlings under positive and negative ion mode, respectively; 12 and 26 unique differential metabolites occurred between needles of WS and WW pine seedlings under positive and negative ion mode, respectively (**Figure 7**; **Table S4**). The intersections of all the four sample groups were 6-shogaol and lupulin A under positive and negative ion mode, respectively (**Table S4**).

Considering totals of specific differential metabolites under positive and negative ion mode, Venn analysis showed that the greatest changes in the number of differential metabolites occurred between needles and roots of WW pine seedlings, in total,186 differential metabolites 125 and 61 unique differential metabolites under positive (**Figure 7A**) and negative (**Figure 7B**) ion mode, respectively. **Figure 7** also showed that the least changes in the number of unique differential metabolites occurred between needles of WS and WW pine seedlings, in total, 38 unique differential metabolites, 22 and 16 metabolites under positive (**Figure 7A**) and negative (**Figure 7B**) ion mode. In addition, 130 unique differential metabolites occurred needles and roots of WS pine seedlings (86 and 44 metabolites under positive and negative ion mode, respectively) and 84 unique differential metabolites occurred between roots of WS and WW pine seedlings (62 and 22 metabolites under positive and negative ion mode, respectively) (**Figure 7**). Interestingly, 337 and 194 differential metabolites were overlapped between needles and roots of WW and WS pine seedlings under positive and negative ion mode, respectively (**Figures 7A, B**). The numbers of the overlapped differential metabolites were the greatest among all the sample groups under positive and negative ion mode, suggesting many metabolites did not show changes under drought stress. All the results suggest that many of the same differential metabolites take part in response to drought stress in needles and roots of *P. taeda* seedlings.

#### 3.2.3 Hierarchical clustering analysis of total differential metabolites

**Figure 8:**
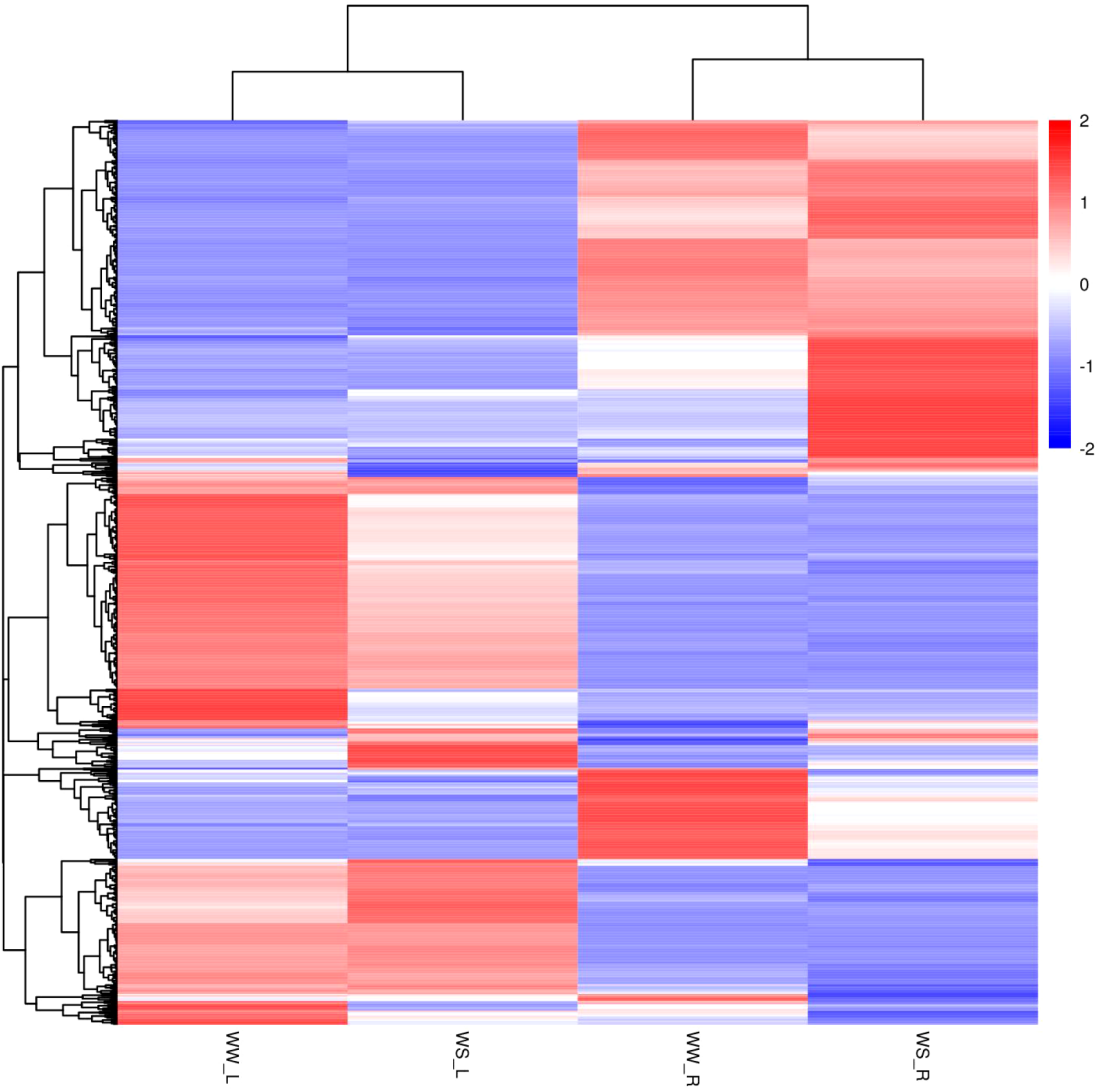
Hierarchical clustering analysis of all differential metabolites from the four groups of samples (positive ion mode). WW-L and WW-R represent needles and roots of well-watered pine seedlings, respectively; WS-L and WS-R represent needles and roots of water-stress pine seedlings, respectively.

In this study, many fatty acids and lipids were identified. Their nomenclatures and classification were carried out according to the LIPID MAPS Lipid Classification System (https://www.lipidmaps.org/). Hierarchical clustering analysis was carried out on all differential metabolites of the individual sample groups under positive ion mode (**Figure 8**) and negative ion mode (**Figure S3**). Under positive or negative ion mode, metabolites in needles and roots of WS pine seedlings showed great differences from those in needles and roots of WW pine seedlings, respectively (**Figures 8**, **S3**).

In detail, under positive ion mode, phosphatidylcholine (PC) (18:5e/2:0), PC (16:1e/2:0), lyso phosphoethanolamine (LPE) 15:1, LPE 16:1, LPE 17:1, monoacylglycerol (MAG) (18:1), LPE 18:2, MAG (18:2), digalactosylmonoacylglycerol (DGMG) (18:2), oleanic acid, oleanolic acid, 4-coumaric acid, botulin, quaillaic acid, betaine, and methyl jasmonate showed lower levels in needles of WW pine seedlings, but salicylic acid, PC (14:0e/2:0), PC (18:3e/2:0), histamine, muramic acid, and N-acetylglucosamine showed higher levels, compared to those in roots of WW pine seedlings (**Figure S4A**). Under negative ion mode, raffinose, sucrose, N-acetylvaline, lyso phosphatidylcholine (LPC) 18:2, phosphatidylethanol (PEtOH) (17:2/20:5), PEtOH (16:2/20:5), benzoic acid, jasmonic acid, quercetin, ɑ,ɑ-trehalose showed higher levels in needles of WW pine seedlings, but LPC 20:3, LPC 16:0, fumaric acid, and D-(-)-quinic acid show lower levels, compared to those in roots of WW pine seedlings (**Figure S4B**).

Under positive ion mode, compared with those in needles of WW pine seedlings, 6-shogaol, lyso phosphatidic acid (LPA) 16:0, phylloquinone, anisic aldehyde showed higher levels in needles of WS pine seedlings (**Figure S4C**), and under negative ion mode, abscisic acid, hirsuteine, asperulosid, topotecan showed higher levels in WS pine needles (**Figure S4D**). Under positive ion mode, LPE 17:1, LPE 18:2, betaine, melatonin, D-(+)-proline, L-glutamine, LPE 15:0, LPE15:1, LPE 16:1, LPE 18: 1, MAG (18:1), MAG (18:2), monogalactosylmonoacylglycerol (MGMG) (18:2), DGMG (18:2), PC (16:1e/2:0), PC (18:5e/2:0), LPC (1-acyl 16:0), oleanolic acid, oleanic acid, and indole-3-acetic acid show lower levels in the needles of WS pine seedlings, but muramic acid, DL-tryptophan, ferulic acid, histidine, coumalic acid, vitamin C, salicylic acid show higher levels in the needles of WS pine seedlings, compared to those in roots of WW pine seedlings (**Figure S4E**). Under negative ion mode, D-proline, PEtOH (18:2/20:5), N-acetylvaline, LPE 14:0, jasmonic acid, sucrose, PEtOH (16:2/20:5), PEtOH (17:2/20:5), fatty acyl esters of hydroxy tatty acid (FAHFA) (20:5/20:4), LPC 18:2, allantoin, L-phenylalanine, quercetin showed lower levels in needles of WS pine seedlings, but ascorbic acid, L-histidine, gluconic acid, MAG (18:3), sorbic acid, lupulin A, abscisic acid, DL-malic acid, and fumaric acid showed higher levels their needles, compared to those in their roots (**Figure S4F**).

Roots of WS pine seedlings showed different patterns of differential metabolites, compared with WW pine roots (**Figures S4G, H**). Under positive ion mode, compared to those in roots of WW pine seedlings, DL-tryptophan, melatonin, cinnamic acid, LPE 14:0, mevalonic acid, and indole-3-acrylic acid showed lower levels in roots of WS pine seedlings, but LPE 18:2, D-(+)-proline, gamma-glutamylglutamine, nicotinic acid, LPE 18:1, histamine, and palmitoleic acid showed higher levels in their roots (**Figure S4G**). Under negative ion mode, D-proline, D-(-)-fructose, LPC16:0, L-citrulline, resveratrol showed higher levels in WS pine roots, compared with those in WW pine roots (**Figure S4H**).

Under positive ion mode, compared with metabolites in needles of WW pine seedlings, some metabolites were significantly up- or down-regulated in needles of WS pine seedlings. The first five up-related metabolites were ingenol-3-angelate, α-aspartylphenylalanine, 3-methoxy prostaglandin F1α, 16,16-dimethyl prostaglandin A2, 7-(2-hydroxypropan-2-yl)-1,4a-dimethyl-decahydronaphthalen-1-ol (WS_L.vs.WW_L_pos_diff.anno in **Table S2**). The first five down-regulated metabolites were cepharanthine, oxymorphone, 4-methyl-5-oxo-2-pentyl-2,5-dihydrofuran-3-carboxylic acid, p-nitroaniline, glycyrrhizin (WS_L.vs.WW_L_pos_diff.anno in **Table S2**).

Under positive ion mode, compared with metabolites in roots of WW pine seedlings, some metabolites were significantly up- or down-regulated in roots of WS pine seedlings. The first five up-regulated metabolites were perillartine, 2-(2-oxo-2-{[2-(2-oxo-1-imidazolidinyl)ethyl]amino}ethoxy)acetic acid, 1-(3,4-dimethoxyphenyl)ethan-1-one oxime, tacrolimus, N-feruloylagmatine (WS_R.vs.WW_R_pos_diff.anno in **Table S2**). Astonishingly, perillartine was up-regulated up to 352 times in roots of WS pine seedlings, compared with that in roots of WW pine seedlings (WS_R.vs.WW_R_pos_diff.anno in **Table S2**). The first five down-regulated metabolites were dehydrotumulosic acid, tetrahydroxyxanthone, phylloquinone, solasonine, (20R)ginsenoside Rh2 (WS_R.vs.WW_R_pos_diff.anno in **Table S2**).

#### 3.2.4 Correlation analysis of differential metabolites

**Figure 9:**
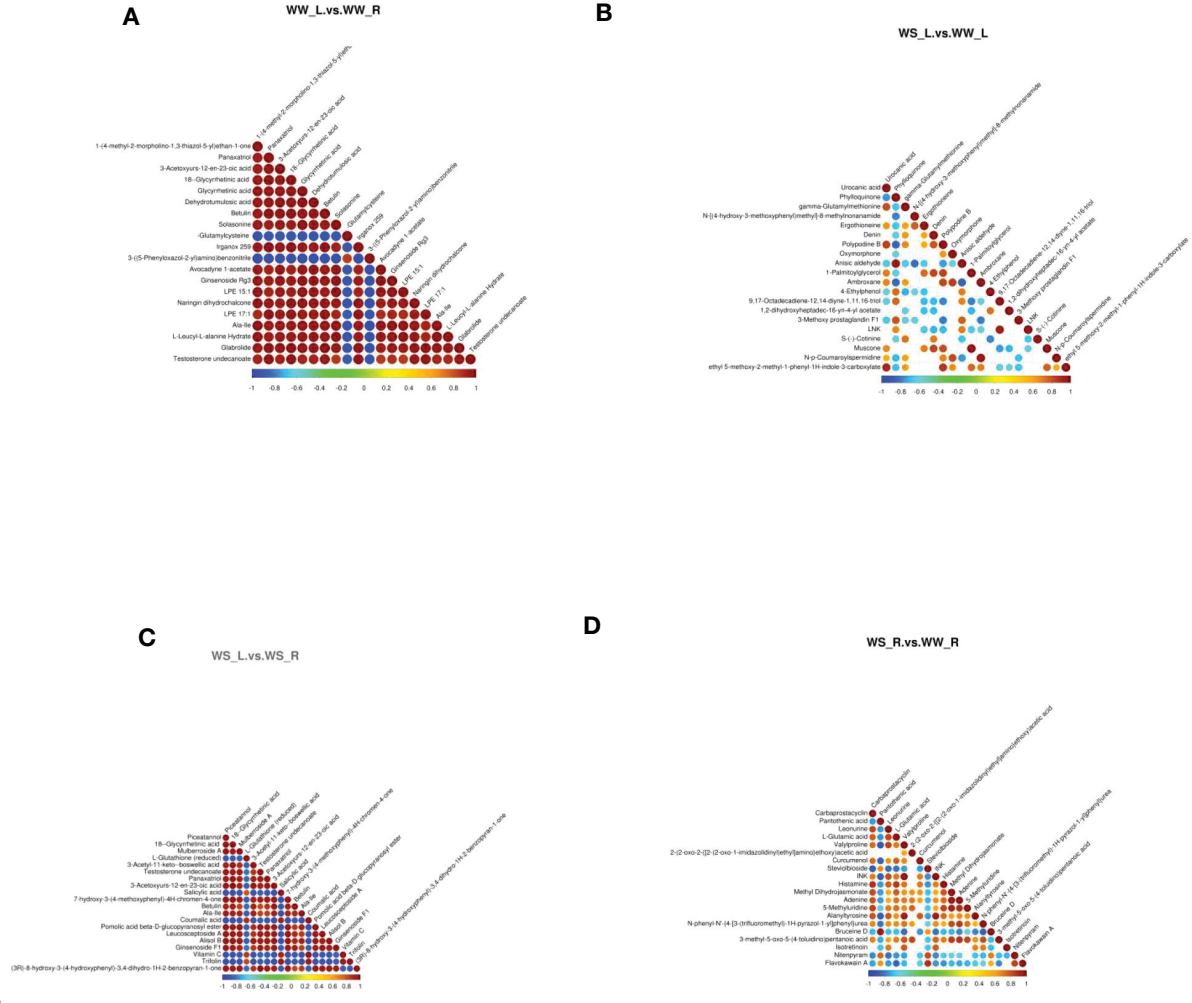
Correlation analysis of metabolites (positive ion mode) with *p* values of top 20 (beginning from the minimal *p* value). The highest correlation with correlation coefficient of 1 was represented in red; the lowest correlation with correlation coefficient of -1 was represented in blue. Colorless parts indicated *p* value > 0.05.  **(A)** leaves vs. roots of WW seedlings; **(B)** leaves of WS vs. WW pine seedlings; **(C)** leaves vs. roots of WS pine seedlings; **(D)** roots of WS vs. WW pine seedlings. WW, well-watered; WS, water-stressed.

**Figure 9** and **Table S4** showed the correlations between differential metabolites in different sample groups under positive ion mode. Comparing metabolites in needles and roots of WW pine seedlings (**Figure 9A** and WW_L.vs.WW_R_pos_corr in **Table S5**), it was found that γ-glutamylcysteine was in negative relation to some metabolites, such as glycyrrhetinic acid, betulin, solasonine, panaxatriol, LPE 15:1, LPE 17:1, and glabrolide. 3-((5-phenyloxazol-2-yl)amino)benzonitrile was also in negative relation to some metabolites, such as LPE 15: 1, LPE 17:1, botulin, and glabrolide. Interestingly, γ-glutamylcysteine and 3-((5-phenyloxazol-2-yl)amino)benzonitrile were in positive relation to each other. Drought stress induced great changes in metabolites in needles of WS and WW pine seedlings (**Figure 9B** and WS_L.vs.WW_L_pos_corr in **Table S5**). Urocanic acid was in positive relation to gamma-glutamylmethionine, polypodine B, and ethyl 5-methoxy-2-methyl-1-phenyl-1H-indole-3-carboxylate, but in negative relation to phylloquinone. Oxymorphone was in positive relation to N-p-coumaroylspermidine (**Figure 9B** and WS_L.vs.WW_L_pos_corr in **Table S5**). Comparing metabolites in needles and roots of WS pine seedlings, it was found that L-glutathine (reduced) was in negative relation to botulin, alisol B, and ginsenoside F1, but in positive relation to salicylic acid, coumalic acid, trifolin, and vitamin C (**Figure 9C** and WS_L.vs.WS_R_pos_corr in **Table S5**). Salicylic acid was in positive relation to coumalic acid, vitamin C, and trifolin, but in negative relation to piceatrannol, mulberroside A, panaxatriol, betullin, Ala-Ile, Alisol B (**Figure 9C** and WS_L.vs.WS_R_pos_corr in **Table S5**). Similarly, drought stress resulted in great changes in metabolites in roots of WS and WW pine seedlings (**Figure 9D** and WS_R.vs.WW_R_pos_corr in **Table S5**). Pantothenic acid was in negative relation to valylproline, curcumenol, alanyltyrosine, but in positive relation to bruceine D (**Figure 9D**, and WS_R.vs.WW_R_pos_corr in **Table S5**). Carbaprostacyclin was in positive relation to leonurine, L-glutamic acid, histamine, adenine, 5-methyluridine, alanyltrosine (**Figure 9D**). L-glutamic acid was in positive relation to leonurine, histamine, methyl dihydrojasmonate, but in negative relation to flavokawain A (**Figure 9D** and WS_R.vs.WW_R_pos_corr in **Table S5**).

Under negative ion mode, great changes occurred in metabolites in needles of WS and WW pine seedlings, (±)-abscisic acid was in positive relation to ganoderic acid C6, asperulosid, 11-deoxyl prostaglandin F2β, MAG (18:3), and topotecan, but in negative relation to neohesperidin (**Figure S5B** and WS_L.vs.WW_L_neg_corr in **Table S6**). Tracheloside was in positive relation to erythronolactone, lupulin A, and saikosaponin B2, but in negative relation to prostaglandin H2 (**Figure S5B** and WS_L.vs.WW_L_neg_corr in **Table S6**).

Under negative mode, comparing changes in metabolites in needles and roots of WS pine seedlings, it was found that ascorbic acid was in positive relation to sorbic acid, D-(-)-quinic acid, sinapaldehyde glucoside, tenuifoliside A, skimming, and gluconolactone, but in negative relation to 20(R)-ginsenoside Rh1, astringin, aloesin, perillic acid, sweroside, bufalin, jasmonic acid, and epimedin B (**Figure S5C** and WS_L.vs.WS_R_neg_corr in **Table S6**).

Comparing changes in metabolites in roots of WS and WW pine seedlings, it was found that D-(+)-maltose was in positive relation to cyaniding-3-*O*-glucoside, prostaglandin D3, and uridine 5’-dephosphogalactose, but in negative relation to sinapoyl *O*-hexoside (**Figure S5D** and WS_R.vs.WW_R_neg_corr in **Table S6**). β-muricholic acid was in positive relation to schizandrol A, 16 ɑ-hydroxyestrone (**Figure S5D** and WS_R.vs.WW_R_neg_corr in **Table S6**).

#### 3.2.5 Z-score analysis

**Figure 10:**
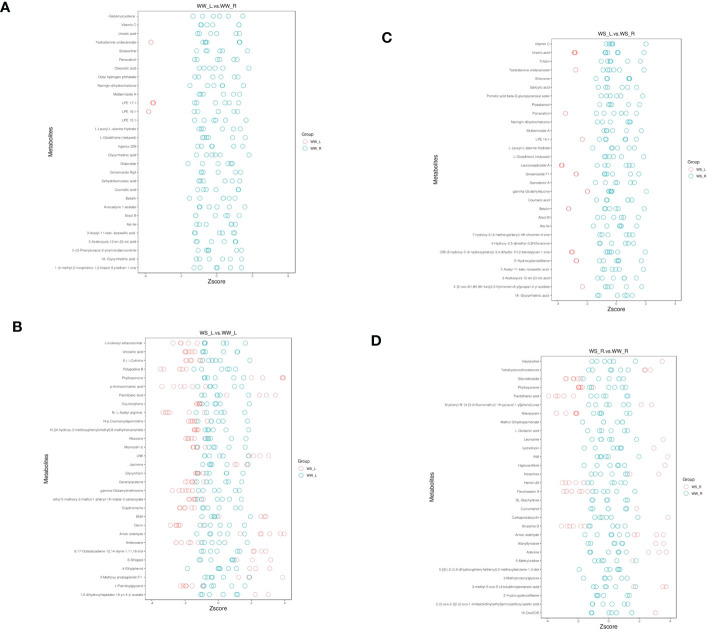
Z score analysis of the four sample groups under positive ion mode. **(A)** leaves vs. roots of WW seedlings; **(B)** leaves of WS vs. WW pine seedlings; **(C)** leaves vs. roots of WS pine seedlings; **(D)** roots of WS vs. WW pine seedlings. WW, well-watered; WS, water-stressed.

Under positive ion mode, Z-score analysis was carried out (**Figure 10** and **Table S7**). Under WW condition, WW pine needles had higher levels of γ-glutamylcysteine, 3-((5-phenyloxazol-2-yl)amino)benzonitrile, octyl hydrogen phthalate, octyl hydrogen phthalate, coumalic acid, and vitamin C (**Figure 10A** and WW_L.vs.WW_R_pos_zscore in **Table S7**). Comparing metabolites in needles of WS and WW pine seedlings, it was found that some metabolites showed higher concentrations in needles of WS pine seedlings, such as phylloquinone, anisic aldehyde, 1,2-dihydroxyheptadec-16-yn-4-yl acetate (**Figure 10B** and WS_L.vs.WW_L_pos_zscore in **Table S7**). Few metabolites showed much higher levels in needles of WS pine seedlings than those in WS pine roots, such as L-glutathione (reduced), salicylic acid, coumalic acid, and vitamin C (**Figure 10C** and WS_L.vs.WS_R_pos_zscore in **Table S7**). Comparing metabolites in roots of WS and WW pine seedlings, it was found that L-glutamic acid, 2-(2-oxo-2-{[2-(2-oxo-1-imidazolidinyl)ethyl]amino}ethoxy)acetic acid, curcumenol showed higher levels in WS pine roots (**Figure 10D** and WW_R.vs.WW_R_pos_zscore in **Table S7**).

Z-scores under negative ion mode were shown in **Figure S6** and **Table S7**. Few metabolites show much higher levels in needles of WW pine seedlings than those in in their roots, such as 2-hydroxymyristic acid, 3,4,5-trihydroxycyclohex-1-ene-1-carboxylic acid, kynurenic acid *O*-hexside, sorbic acid (**Figure S6A** and WW_L.vs.WW_R_neg_zscore in **Table S7**). Under negative ion mode, ganoderic acid C6, asperulosid, 11-deoxy prostaglandin F2β, MAG (18:3), hirsuteine, and (±)-abscisic acid showed higher concentrations in needles of WS pine seedlings than those in needles of WW pine seedlings (**Figure S6B** and WS_L.vs.WW_L_neg_zscore in **Table S7**). Under negative ion mode, 3,4,5-trihydroxycyclohex-1-ene-1-carboxylic acid, sorbic acid, D-(-)-quinic acid, tenuifoliside A, and abscorbic acid showed higher levels in WS pine needles, compared with their roots (**Figure S6C** and WS_L.vs.WS_R_neg_zscore in **Table S7**). Comparing metabolites in roots of WS and WW pine seedlings, it was found that ethyl 3-cyano-6-methyl-2-(phenylthio)isonicotinate, 1,3,5-trimethoxybenzene, ganoderic acid C6, L-citrulline, 16α-Hydroxyestrone, and 4-oxoproline showed higher concentrations in roots of WS pine seedlings (**Figure S6D** and WS_R.vs.WW_R_neg_zscore in **Table S7**).

#### 3.2.6 KEGG enrichment analysis

**Figure 11:**
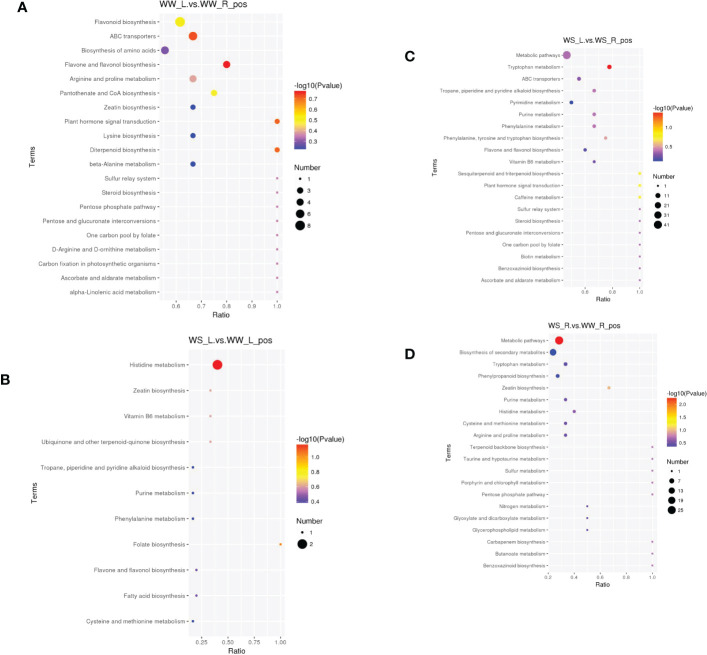
Metabolites enriched in KEGG pathways under positive ion mode (top 20 pathways). In the small figures, horizontal axis represented the ratio of number of differential metabolites to the total numbers of metabolites identified in a pathway. Greater the ratio was, higher the differential metabolites enriched in the pathway. Color of a circle represented *p* value in geometric test. The size of a circle represented the number of differential metabolites in the corresponding pathway. **(A)** needles vs. roots of WW seedlings; **(B)** needles of WS vs. WW pine seedlings; **(C)** needles vs. roots of WS pine seedlings; **(D)** roots of WS vs. WW pine seedlings. WW, well-watered; WS, water-stressed.

The most enriched metabolite pathways under positive ion mode were shown in **Figure 11** and **Table S8**. Comparing needles and roots of WW pine seedlings, the first five enriched pathways included flavone and flavonol biosynthesis, ABC transporters, plant hormone signal transduction, flavonoid biosynthesis, and pantothenate and CoA biosynthesis (**Figure 11A** and WW_L.vs.WW_R_pos_kegg_enrichment in **Table S8**). Comparing needles of WS and WW pine seedlings, the first five enriched pathways included histidine metabolism, folate biosynthesis, ubiquinone and other terpenoid-quinone biosyntehsis, vitamin B6 metabolism, and zeatin biosynthesis (**Figure 11B** and WS_L.vs.WW_L_pos_kegg_enrichment in **Table S8**). Comparing needles and roots of WS pine seedlings, the first five enriched pathways included tryptophan metabolism, caffeine metabolism, sesquiterpenoid and triterpenoid biosynthesis, plant hormone signal transduction, and phenylalanine, tyrosine and tryptophan biosynthesis (**Figure 11C** and WS_L.vs.WS_R_pos_kegg_enrichment in **Table S8**). Comparing roots of WS and WW pine seedlings, the first five enriched pathways included metabolic pathways, zeatin biosynthesis, pentose phosphate pathway, carbapenem biosynthesis, and benzoxazinoid biosynthesis (**Figure 11D** and WS_R.vs.WW_R_pos_kegg_enrichment in **Table S8**).

Under negative ion mode, the most enriched pathways were shown in **Figure S7** and **Table S9**. Comparing needles and roots of WW pine seedlings, the first five enriched pathways included phenylalanine metabolism, butanoate metabolism, starch and sucrose metabolism, anino sugar and nucleotide sugar metabolism, and phyenylpropanoid biosynthesis (**Figure S7A** and WW_L.vs.WW_R_neg_kegg_enrichment in Table **Table S9**). Comparing needles of WS and WW pine seedlings, the first five enriched pathways included biosynthesis of unsaturated fatty acids, arachidonic acid metabolism, plant hormone signal transduction, carotenoid biosynthesis, and porphyrin and chlorophyll metabolism (**Figure S7B** and WS_L.vs.WW_L_neg_kegg_enrichment in **Table S9**). Comparing needles and roots of WS pine seedlings, the first three enriched pathways included phenylalanine metabolism, tyrosine metabolism, butanoate metabolism (**Figure S7B** and WS_L.vs.WS_R_neg_kegg_enrichment in **Table S9**). Comparing roots of WS and WW pine seedlings, the first five enriched pathways included arginine and proline metabolism, lysine degradation, tryptophan metabolism, biosynthesis of unsaturate fatty acids, biosyntehsis of amino acids (**Figure S7B** and WS_R.vs.WW_R_neg_kegg_enrichment in **Table S9**).

## 4 Discussion

### 4.1 Roles of phytohormomes in *P. taeda* under drought stress

Phytohormones regulate plant growth and response to abiotic/biotic stress, especially ABA under drought stress (Yu et al., 2019; Zhang et al., 2020; Wang et al., 2021b; Muhammad Aslam et al., 2022). Under long-term drought, ABA concentration significantly was higher in needles of WS pine seedlings than those in the other samples, but ABA levels did not show differences among roots of WS pine seedlings and needles and roots of WW pine seedlings (**Figure 4A**). Our untargeted metabolomics analysis also showed ABA level increased about 50% in needles of WS pine seedlings, but slightly increased in their roots (**Table S10**, negative ion mode). The results suggest that ABA played an important role in controlling water loss by transpiration. At present, it has been well known about the mechanism that ABA regulates stomatal closure. The activation of a slow anion current in guard cells is a key event resulting in stomatal closure (Schroeder and Hagiwara, 1989; Pandey et al., 2007; Roelfsema et al., 2012; Saito and Uozumi, 2019). Environmental stresses induce increase in ABA biosynthesis and ABA leads to phosphoactivation of slow anion channel 1 (SLAC1), and SLAC1 reduces turgor pressure in guard cells and stomatal closure occurs (Brandt et al., 2012; Hedrich and Geiger, 2017). Recently, Deng et al. (2021) analyzed structure and activity of SLAC1 channels and confirmed that SLAC1 phosphorylaltion fine-tunes equilibrium between basal and activated SLAC1 trimers controlling the degree of stomatal opening. Similarly, salicylic acid (SA) concentration was significantly higher in WS pine needles than that in WW pine needles and showed no difference in roots of WW and WS pine seedlings (**Figure 4C**). Some experimental results showed that SA can induce stomatal closure (Manthe et al., 1992; Khokon et al., 2011; Poór and Tari, 2012). SA-induced stomatal closure is related to increased levels of reactive oxygen species (ROS) and nitric oxide (NO) (Poór and Tari, 2012). Previous research supported our results of targeted metabolomics analysis (Sharma et al., 2017; Khan et al., 2021; Ahmad et al., 2021). Ahmad et al. (2021) reported that exogenous SA induced drought tolerance in wheat (*Triticum aestivum*) grown under hydroponic culture and it was confirmed that plants increase SA accumulation after being exposed to drought stress (Okuma et al., 2014). Therefore, increased SA levels in needles of WS pine seedlings are helpful stomatal closure, further reducing water loss by transpiration under drought stress. During control over stomatal closure, SA interacts with ABA and shares components of ABA signaling pathway (Khokon et al., 2017; Bharath et al., 2021).

In contrast to the situations of ABA and SA mentioned above, GA4 concentration was significantly higher in roots of WS pine seedlings than that in roots of WW pine seedlings (**Figure 4D**). In grafted tomato plants, constitutive gibberellin response modulated root-to-shoot signaling under drought stress (Gaion et al., 2018). The results from Gaion et al. (2018) showed that the GA sensitivity of the rootstock modulated stomatal conductance and water use efficiency under drought stress, indicating that GA and ABA crosstalk in the adjustment of growth and water economy. Transcriptome survey and expression analysis revealed that pear mRNA expression of enzyme genes involved in gibberellin signaling was higher than that in control in response to long-term drought stress (Yang et al., 2021). However, gibberellins occur in many structures in plants, and they did not show consistent roles in plants under long-term drought stress, such as GA3 and GA4 (**Figures 4C, D**). GA3 levels increased in WS pine needles, compared with that in WW pine needles (**Figure 4C**), and untargeted metabolomics analysis showed that GA3 levels increased in WS pine roots and needles (**Table S2** and positive ion mode in **Table S10**). A report showed drought triggered reduction in RWC and chlorophyll concentrations, but these parameters recovered when droughty plants of sugarcane (*Saccharum* spp. hybrids) were exposed to GA3 (Tripathi et al., 2019). Therefore, increased GA3 levels in WS pine roots and needles could improve drought tolerance of *P. taeda* seedlings.

Drought stress provokes JA signaling and JA is involved in root development under drought stress by the antagonistic interaction with cytokinin (Jang et al., 2017; Jang and Choi, 2018; Ali and Baek, 2020). But in our research, JA and JAI did not show significant changes in roots and needles of WW and WS pine seedlings (**Figure 4B**), however, JA increased 91% in WS pine roots, compared with WW pine seedlings (**Figure 4B**). In addition, untargeted metabolomics analysis showed that levels of JA and methyl jasmonate (MJ) increased in roots and needles of WS pine seedlings, compared with WW pine seedlings (**Table S2** and negative ion mode in **Table S10**). Previous research confirmed that water stress increases JA concentrations in leaves and roots of rice (*Oryza sativa*) plants (Kiribuchi et al., 2005). Thus increased JA levels in WS pine seedlings can promote drought tolerance of *P. taeda* seedlings (Wasternack and Song, 2017).

### 4.2 Roles of sugars and amino acids in *Pinus taeda* under drought stress

Sugars maintain energy activity in plant cells and show their roles as osmoregulators and membrane protectors (Sami et al., 2016), thus they often show great responses to environmental changes. In this study, glucose concentrations showed no statistical difference between needles of WW and WS pine seedlings, but glucose concentration significantly reduced in WS pine roots (**Figure 1B**). High glucose accumulation in WS pine needles (**Figure 1B**) is helpful for controlling water loss, because glucose can induces stomatal closure (Osakabe et al., 2013). Sucrose is a main sugar transported from source to sink organs in most plants, and carbon allocation between source and sink organs decides fate of sink organs. Untargeted metabolomics analysis showed that sucrose levels increased in roots and needles of WS pine seedlings, compared with those in roots and needles of WW pine seedlings, respectively (**Table S10**, negative ion mode). Sucrose concentrations in WS pine needles were significantly lower than those in WW pine seedlings, but significantly higher in roots of WS pine seedlings (**Figure 1A**). All the results suggest sucrose role in *P. taeda* seedlings under drought stress. The sucrose accumulation in roots under drought stress is helpful to increase root activity and ability to explore water source.

Compared with WW pine seedlings, raffinose level increased in roots of WS pine seedlings (**Figure 1B**), and untargeted metabolomics analysis showed that raffinose levels in roots and needles of WS pine seedlings also increased (**Table S10**, negative ion mode). Previous research showed that raffionose family oligosaccharides have important roles against abiotic stress (ElSayed et al., 2014; Mukherjee et al., 2019), thus increase in raffinose levels in roots and needles of WS pine seedlings is helpful for enhanced tolerance to drought stress.

Some experimental results showed that trehalose, acting as an osmoprotectant, plays an important role in plants under drought stress (Ilhan et al., 2015; Kosar et al., 2021; Mukarram et al., 2021; Zulfiqar et al., 2021). However, in our targeted metabolomics analysis, trehalose was not detected in roots and needles of WW and WS pine seedlings. This result suggests trehalose levels should be very low, as reported by Ingram & Bartels (1996). Our untargeted metabolomics analysis showed that trehalose levels increased in roots and needles of WS pine seedlings, compared with those in roots and needles of WW pine seedlings, respectively (**Table S10**, negative ion mode). The result is consistent with that reported by Mibei et al. (2018). Their metabolomics analysis showed that trehalose level increased in African eggplants (*Solanum aethiopicum*) under drought stress. Thus it might be speculated that trehalose played a role in pine seedlings under drought stress, just like its role in other plant species.

In roots and needles of WS pine seedlings, the enriched KEGG pathways were involved in biosynthesis and metabolism of secondary metabolites (**Figures 11**, **S7**; **Tables S8**, **S9**), suggesting that amiono acids played important roles in *P. taeda* seedlings under long-term drought stress. Some amino acids were studied well about their roles under drought stress. For example, proline acts as an osmoprotectant against drought stress in plants (Furlan et al., 2020; Adamipour et al., 2020; Mukarram et al., 2021). Proline levels increased in roots of WS pine seedlings, but not significantly, compared with that in roots of WW pine seedlings (**Figure 2A**). Similarly, untargeted metabolomics analysis showed increased proline level in roots of WS pine seedlings, compared with that in roots of WW pine seedlings (**Tables S2**, **S10**, negative ion mode). Results from Díaz et al. (2010) showed that deficiency in plastidic glutamine synthetase alters proline metabolism and transcriptomic response in *Lotus japonicas* under drought stress. Since glutamine synthetase is in charge of conversion of ammonium to glutamine, glutamine is related to proline metabolism, because deficiency in plastidic glutamine synthetase alters proline metabolism in Lotus japonicus under drought stress (Díaz et al., 2010) and glutamine synthetase in the phloem plays a major role in controlling proline production (Brugiere et al., 1999). Our results showed that glutamine levels significantly increased in roots of WS pine seedlings, compared with that in WW pine seedlings (**Figure 2A**), and untargeted metabolomics analysis also showed glutamine levels significantly increased in WS pine roots, compared with those in WW pine roots (**Tables S2**, **S10**, negative ion mode). Therefore, significant increase in glutamine levels in roots of WS pine seedlings is helpful for enhanced drought tolerance of *P. taeda* seedlings by regulating proline metabolism. In addition, proline derivative, DL-stachydrine (proline betaine, a quaternary ammonium derivative of proline that occurs widely in *Medicago* species), was up-regulated in WS pine roots (**Table S2**). Proline betaine accumulated in alfalfa (*Medicago sativa*) plants under salt stress (Trinchant et al., 2004), however, at present, no information has been known about the function of DL-stachydrine in plants under drought stress.

Previous research showed that arginine and aspartate play roles in plants under drought stress (Hasanuzzaman et al., 2018; You et al., 2019; Ali et al., 2021). In roots of WS pine seedlings, levels of arginine and aspartate increased, compared with those in roots of WW pine seedlings, respectively (**Figure 2B**), but untargeted metabolomics analysis showed that levels of L-aspartic acid in roots and needles of WS pine seedlings reduced, compared with those in WW pine seedlings, respectively (**Table S10**, positive ion mode). The inconsistency might stem from sampling differentiation. Based on targeted analysis results, the two amino acids should possess the same roles in *P. taeda* seedlings under drought stress as they function in other plant species.

The saccharopine pathway (SACPATH) involves the conversion of lysine into α-aminoadipate by three enzymatic reactions catalyzed by the bifunctional enzyme lysine-ketoglutarate reductase/saccharopine dehydrogenase (LKR/SDH) and the enzyme α-aminoadipate semialdehyde dehydrogenase (AASADH) (Arruda and Barreto, 2020). Level of free lysine significantly increased in drought-tolerant and drought susceptible sesame (*Sesamum indicum*) under drought stress (You et al., 2019), suggesting role of lysine in plants under drought stress. Our results showed that lysine level significantly increased in roots of WS pine seedlings, compared with that in roots of WW pine seedlings (**Figure 2C**). The significant increase in lysine level in roots of WS pine seedlings resulted in significant reduction in L-saccharopine and significant increase in pipecolic acid (**Tables S2**, **S10**). Both saccharopine and pipecolic acid stem from lysine, but pipecolic acid is synthesized from saccharopine (Arruda and Barreto, 2020), thus it is reasonable to explain significant reduction in saccharopine level and increase in pipecolic acid level. Furthermore, our result is consistent with previous research which showed that saccharopine levels significantly reduced in drought-tolerant wheat (*Triticum aestivum*) genotypes HX10 under drought stress (Guo et al., 2020).

Asparagine is synthesized from glutamine by the reaction of aspargine synthetase (AS). The up-regulation of AS gene may contribute the higher leaf nitrogen remobilization in maize (*Zea mays* L.) when exposed to drought stress (Li et al., 2016). Our results showed that aspargine level significantly increased in WS pine roots, compared with that in WW pine roots (**Figure 2C**). The result is consistent with the previous one.

Targeted metabolomics analysis showed that levels of L-tyrosine reduced in WS pine roots (**Table 1**), and untargeted metabolomics analysis showed that tyrosine reduced in roots and needles of WS pine seedlings (**Table S2** and positive ion mode in **Table S10**). However, previous research showed that tyrosine levels increased and suggested that tyrosine accumulation might play a positive role in response to drought stress (Liu et al., 2012; Khan et al., 2019; Tong et al., 2020). Thus, the function of tyrosine in plants under drought stress should be investigated in detail.

### 4.3 Roles of fatty acids and lipids in *Pinus taeda* under drought stress

Some lipids contain fatty acids. Fatty acids possess three main functions, i.e., membrane components, energy metabolism and store, and signaling. Some lipids with fatty acids are important components of biological membranes in plants. For example, phosphatidylcholine (PC) and phosphoethanolamine (PE) are the major glycerolipids of the cell membranes, and mitochondria membrane and endoplasmic reticulum membranes, and chloroplast membranes mainly contain monogalactosyldiacylglycerol (MGDG) and digalactosyldiacylglycerol (DGDG) (Zhukov and Shumskaya, 2020). In higher plants, the most common unsaturated fatty acids (UFAs) are three 18-carbon species, namely, oleic (18:1), linoleic (18:2), and a-linolenic (18:3) acids, and these UFAs possess multiple functions in plants, especially under abiotic stress (He and Ding, 2020). Untargeted metabolomics analysis showed that the most enriched KEGG pathway was biosyntehsis of unsaturated fatty acids in needles of WS pine seedlings under negative ion mode (**Figure S7** and WS_L.vs.WW_L_neg_kegg_enrichem in **Table S9**); under positive ion mode, fatty acid biosynthesis was one of the most enriched pathways in WS pine needles (**Figure 11B** and WS_L.vs.WW_L_pos_kegg_enrichment in **Table S8**), suggesting that great changes in fatty acids in WS pine needles. Targeted metabolomics analysis showed that levels of some fatty acids significantly reduced in roots and/or needles of WS pine seedlings, compared with WW pine seedlings (**Table 2**). The significant reduction in fatty acid levels might be based on the reason that plants reduce carbon fixation under drought stress, further affecting biosynthesis of fatty acids. However, three saturated fatty acids significantly increased in WS pine seedlings, i.e., dodecanoic acid in needles, and tricosanoic acid and heptadecanoic acid in roots (**Table 2**). At present, little has been known about their functions in plants under drought stress.

Untargeted metabolics analysis showed that LPA 14:00 and LPA 16:0 showed higher levels in needles of WS pine seedlings (**Figures S4C, D**), and that LPC16:0, LPE 18:2, LPE 18:1, and palmitoleic acid showed higher levels in their roots (**Figures S4G, H**), compared with those in WW pine roots and needles, respectively. Similarly, compared with those in needles of WS pine seedlings, some unsaturated lipids had higher levels in their roots, such as LPC15:1, LPE15:1, LPE16:1, LPE17:1, LPE 18:1, LPE18:2, MAG (18:1), MAG (18:2), MGMG (18:2), DGMG (18:2), PC (16:1e/2:0), PC (18:5e/2:0), PEtOH (18:2/20:5), PEtOH (16:2/20:5), PEtOH (17:2/20:5), FAHFA (20:5/20:4), LPC 18:2 (**Figures S4E, F**). Their increased levels improved membrane flexibility in roots and needles of *P. taeda* seedlings under drought stress. Previous studies supported our results (Gu et al., 2020). As mentioned above, in needles of WS pine seedlings, ABA levels significantly increased (**Figure 4A**) and (±)-ABA was in positive relation to MAG (18:3) (**Figure S5B** and WS_L.vs.WW_L_neg_corr in **Table S6**), suggesting that ABA accumulation might improve unsaturated lipid biosynthesis in WS pine needles and sustain membrane stability and flexibility under drought stress, because ABA improves biosynthesis of some fatty acids in plants (Norlina et al., 2020; Shi et al., 2021).

### 4.4 Roles of neurotransmitters in *Pinus taeda* under drought stress

Neurotransmitters play important roles in plant growth/development and responses to environmental stresses (Erland et al., 2018; Akula and Mukherjee, 2020; Qin et al., 2020; Sun et al., 2021). Among the neurotransmitters in plants, melatonin is up to now the most important one in response to drought stress. Melatonin enhances drought stress tolerance (Ahmad et al., 2019; Tiwari et al., 2021; Imran et al., 2021; Ren et al., 2021), and the regulatory mechanisms are involved in many aspects (Tiwari et al., 2021). In our research, melatonin was not determined in WS pine needles, and melatonin concentrations in roots showed no difference between WW and WS pine seedlings (**Table 1**). But untargeted metabolomics analysis showed that melatonin levels in WS pine roots and needles significantly increased (**Table S2** and positive ion mode in **Table S10**). The results might suggest that melatonin still play an important role in *P. taeda* seedlings under long-term drought stress.

At present, little knowledge was known about function of glutamine, acting as a neurotransmitter, in plants under drought stress. Under drought stress, glutamine synthetase (GS, EC 6.3.1.2) showed different activities in drought-sensitive (cv. IR-64) and drought-tolerant (cv. Khitish) rice (*Oryza sativa*) cultivars, and OsGS2 and OsGS1;1 may contribute to drought tolerance of drought-tolerant cultivar Khitish under drought stress (Singh and Ghosh, 2013). Another report showed GS expression was up-regulated in maize leaves under drought stress (Li et al., 2016). The overexpression of wheat (*Triticum aestivum*) cytosolic and plastic GS in tobacco enhanced drought tolerance of tobacco (*Nicotiana tabacum*) (Yu et al., 2020). All the results indicate that glutamine synthesized through GS activity possesses an important role in plants under drought stress. In the GS2 mutant (*Ljgln2-2*) of *Lotus japonicas* under drought stress, proline accumulation was substantially lower than that in WT plants (Díaz et al., 2010), suggesting GS2 was involved in proline biosynthesis, thus implying that glutamine might be related to proline biosynthesis. Since GS converts ammonium to glutamine, up-regulated GS expression means increase in glutamine biosynthesis. Therefore, glutamine might play a role in response to drought stress *via* proline metabolism. In our research, among the neurotransmitters detected, L-glutamine concentrations showed the greatest changes in roots, i.e., significant increase in roots of WS pine seedlings (**Figure 2A**; **Table 1**), and untargeted metabolomics analysis also showed that L-glutamine significantly increased in roots and needles of WS pine seedlings (**Table S2** and positive ion mode in **Table S10**), compared with WW pine seedlings, suggesting that our result is consistent with the previous results mentioned above. Although proline concentrations in roots of WS pine seedlings did not show significant changes, compared with WW pine seedlings (**Figure 2A**), untargeted metabolomics analysis showed that proline levels significantly increased in WS pine roots, compared with that in WW pine roots, suggesting a close relationship between glutamine and proline accumulation.

Acetylcholine showed different concentration patterns in roots and needles of WW and WS pine seedlings, i.e., acetylcholine concentration was significantly higher in WS pine needles, but significantly lower in WS pine roots, compared with WW pine seedlings, respectively (**Table 1**). In addition, hyoscyamine and atropine, acting as antagonists of acetylcholine receptors, significantly increased in WS pine roots, compared with those in WW pine roots, and was not determined in needles of WW and WS pine seedlings (**Table S2**). A research report showed acetylcholine regulates stomatal movement *via* nicotinic acetylcholine receptors (Wang et al., 1998). Furthermore, acetylcholine acts as a long-distance signaling molecule (Lou, 1998; Jia and Zhang, 2008). Therefore, significant acetylcholine increase in needles and reduction in roots of WS pine seedlings might suggest that acetylcholine was delivered from roots to needles of WS pine seedlings under drought stress, acting as a long-distance signaling molecule, as mentioned above. Significant increase in atropine and hyoscyamine levels in WS pine roots might result in their binding to acetylcholine receptors prior to acetylcholine and make more acetylcholine transport to needles under drought stress. However, results from Wang et al. (1998) showed that acetylcholine induced stomatal opening. Our results showed that acetylcholine concentration increased about 3 times in WS pine needles, compared with that in WW pine needles (**Table 1**), therefore, we speculate that acetylcholine acts as a two-tier regulator, i.e., acetylcholine regulates stomatal opening under WW condition, and under WS condition acetylcholine is transported from roots to needles and accumulates to higher levels as to induce stomatal closure, just like 2,4-dichlorophenoxyacetic acid (2,4-D) acting as an herbicide when its level is higher and an auxin analog when its level is lower (Song, 2014; Agathokleous et al., 2019). Based on the fact that our result about acetylcholine levels is contrary to that of Wang et al. (1998), more research on functions of acetylcholine is needed, especially its regulating stomatal movement under drought stress.

Dopamine (3-hydroxytyramine) is an important neurotransmitter in animals, and show many roles in plant physiology. Exogenous dopamine improved the tolerance against several abiotic stresses, such as drought, salt, and nutrient stress (Liu et al., 2020). Pretreatment with 100 μM dopamine alleviated drought stress in apple (*Malus domestica*) seedlings, inhibited the degradation of photosynthetic pigments, and increased net photosynthetic rate under drought stress (Gao et al., 2020). Their results also showed that dopamine may improve apple drought tolerance by activating Ca^2+^ signaling pathways through increased expression of CNGC and CAM/CML family genes, and analysis of transcription factor expression suggested that dopamine affected drought tolerance mainly through the regulation of WRKY, ERF, and NAC transcription factors (Gao et al., 2020). Thus, our results, i.e., significant increase in hydroxytyramine level in WS pine needles (**Table 1**), suggest its role in *P. taeda* seedlings under long-term drought stress.

Little is known about the role of the neurotransmitter noradrenaline in plants. It was reported that heat-treated noradrenaline induced flowering in short-day *Lemna paucicostata* strains 441 and 6746 and long-day *Lemna gibba* strain G3 (Miyawaki et al., 2013). In our research, noradrenaline concentration significantly reduced in WS pine needles and had no difference in their roots, compared with WW pine seedlings (**Table 1**), suggesting noradrenaline might be related to controlling water loss under drought stress. If so, the relationship between ABA signaling and noradrenaline in plants under drought stress should be investigated.

Increased accumulation of γ-aminobutyric acid (GABA) during abiotic stresses is well documented, especially drought stress (Mekonnen et al., 2016; Li et al., 2018; Li et al., 2020), and exogenous application of GABA can mitigate drought stress (Abd El-Gawad et al., 2021; Abdel Razik et al., 2021), therefore, GABA acts as a key player in drought tolerance in plants (Hasan et al., 2021a). However, in our research, GABA significantly reduced in WS pine needles and reduced in WS pine roots, compared with those in WW pine seedlings, respectively (**Table 1**). The result is consistent with that reported by Khan et al. (2019). Their results showed that GABA levels decreased in both drought-tolerant and -sensitive lines of chickpea (*Cicer arietinum*) under drought stress. Based on our results and previous results, the neurotransmitter should be investigated in detail about its function in plants under drought stress.

### 4.5 Roles of organic acids in *Pinus taeda* under drought stress

Previous research showed that levels of some organic acids increased in plants under drought stress, such as alpha-phocaecholic acid, kynurenic acid, homovanillic acid, gallic acid, picolinic acid in drought-tolerant wheat genotype HX10 under drought stress (Guo et al., 2020). Our results also showed that few organic acids increased in response to long-term drought in *P. taeda* seedlings, such as 5-hydroxymethyl-2-furoic acid in needles, tartaric acid, malonic acid, citric acid, and malic acid in roots (**Figure 3**). Untargeted metabolomics analysis showed that gallic acid reduced in roots and needles of WS pine seedlings (**Table S2** and positive ion mode in **Table S10**). The result is in contrast to that reported by Guo et al. (2020). They reported level of gallic acid increased more than 3 times in drought-tolerant wheat genotype HX10, compared with that in drought-sensitive wheat genotype YN211 when exposed to drought stress. Maybe increased gallic acid level is a rapid response to drought stress and its level reduces when exposed long-term drought stress. Our result also showed that syringic acid increased in WS pine roots (Table 9, positive ion mode). The result is also in contrast to that reported by Guo et al. (2020). They reported that syringic acid reduced in drought-tolerant wheat genotype HX10. Kynurenic acid significantly reduced in roots and needles of WS pine seedlings (**Table S2**). The result about kynurenic acid is also in contrast to that reported by Guo et al. (2020). We cannot explain the two differences. A report showed high level of ferulic acid in drought-resistant winter triticale (Triticosecale Wittmack) genotype Lamberto under drought stress (Hura et al., 2009) and its drought resistance was correlated with high free ferulic acid level in leaf tissues (Hura et al., 2007). Ferulic acid bound with carbohydrates of the cell-wall of Lamberto under drought stress and the markedly better parameters of chlorophyll fluorescence for this genotype under both treatments correlated strongly and positively with the high contents of cell-wall-bound ferulic acid (Hura et al., 2009). However, our untargeted metabolomics analysis showed that ferulic acid significantly reduced in roots and needles of WS pine seedlings (**Table S2** and negative ion mode in **Table S10**). This suggests that ferulic acid had less function in *P. taeda* seedlings under long-term drought stress. In drought-tolerant leafy vegetable amaranth (*Amaranthus tricolor*) genotypes (VA16, VA14, VA11, and VA6), levels of ellagic acid showed significant changes among these genotypes (the highest level, 6.23 µg·g^-1^ FW, in VA16), but no situation was observed about ellagic acid levels in these amaranth genotypes under drought stress (Sarker and Oba, 2020). In this study, ellagic acid reduced in roots and needles of WS pine seedlings (**Table S10**, negative ion mode). Reduction in ellagic acid in roots and needles of WS pine seedlings suggests less importance of ellagic acid in *P. taeda* seedlings under long-term drought stress. Caffeic acid slightly reduced in needles of WS pine seedlings, and showed no change in their roots (**Table S10**, positive ion mode). Since caffeic acid is involved in melatonin biosynthesis and caffei acid *O*-methyltransferase gene confers melatoin-mediated drought tolerance (Chang et al., 2021; Li et al., 2022), function of caffei acid should be related to melatonin in plants under drought stress. *p*-coumaric acid ethyl ester increased in roots and needles of WS pine seedlings (**Table S2** and positive ion mode in **Table S10**). At present, little is known about funtion of *p*-coumaric acid ethyl ester in plants under drought stress. Based on our results of organic acids mentioned above, it was known that these organic acids showed great differences in their levels in response to long-term drought stress in *P. taeda* seedlings, and exhibited response differences of these organic acids from other plant species. In general, little has been known about detailed functions of organic acids in plants under drought stress. Therefore, more research is needed in view of some contradictory results mentioned above.

### 4.6 Roles of flavonoids, terpenoids, and other small molecules in *Pinus taeda* under drought stress

Flavonoids possess many physiological functions in plants, especially under abiotic stress (Treutter, 2005; Falcone Ferreyra et al., 2012). Under drought stress, some genes involved in flavonoids biosynthesis were up-regulated in the leaves of hybrid poplar (*Populus tremula* × *Populus alba*), such as these genes encoding phenylalanine ammonia-lyase, 4-coumarate CoA ligase, chalcone synthase, flavonol synthase, flavanone 3-hydroxylase, dihydroflavonol-4-reductase, and anthocyanidin synthase (Ahmed et al., 2021). Levels of quercetin-3-*O*-rutinoside and dihydroxyflaone luteolin 7-*O*-glucoside increased in leaves of *Ligustrum vulgare* under drought stress, compared with well-watered plants (Tattini et al., 2004). In *Achillea pachycephala*, flavonoid biosynthesis responded to drought stress, including apigenin-7-O-glycoside, luteolin, apigenin and kaempferol (Gharibi et al., 2019). Our untargeted metabolomics identified some unique flavonoids in *P. taeda* seedlings under drought stress (**Table S2**, **S3**).

Rutin significantly reduced in roots and needles of WS pine seedlings, but quercetin significantly increased in their roots and needles, compared with WW pine seedlings, respectively (**Table S10**, negative ion mode). Two other flavonoids, isorhamnetin and myricetin, significantly reduced in roots and needles of WS pine seedlings, compared with WW pine seedlings (**Table S2** and positive ion mode in **Table S10**). Our results about rutin and quercetin are consistent with that reported by Yang et al. (2020) and Su et al. (2020), but our result about isorhamnetin is in contrast to that in *Bupleurum chinense* reported by Yang et al. (2020). The contrary results might be related to plant species.

In addition, compared with WW pine seedlings, epigallocatechin increased in WS pine needles (**Table S2**); naringenin increased in roots and needles of WS pine seedlings (**Table S10**, positive ion mode); catechin levels did not show changes in roots and needles of WS pine seedlings (**Table S10**, negative ion mode); apigenin C-glucoside increased in WS pine roots (**Table S2**). However, drought stress resulted in down-regulation of proteins involved in flavonoids biosynthesis in tea (*Camellia sinensis*) seedlings, such as chalcone synthase, flavanone 3-hydroxylase, flavonol synthase (Gu et al., 2020). The down-regulation might be related to reduction in the biosynthesis of these flavonoids in tea seedlings under drought stress. In general, WS pine seedlings showed different patterns of flavonoids and their spatial distribution and increase or reduction in levels are related to response of *P. taeda* to long-term drought stress.

Previous research showed contradictory results about terpenoids response to drought stress. Some research showed that increased levels of terpenoids in leaves to respond to drought stress (Sangwan et al., 2001; Delfine et al., 2005; Asensio et al., 2012; Yadav et al., 2014; Kleine and Müller, 2014). Similarly, previous research found that terpenoid levels in plant roots responded to drought stress in contradictory ways (Bezemer et al., 2004; Asensio et al., 2012; Kleine and Müller, 2014). Saikosaponins are oleanolic triterpenoid saponins. Yang et al. (2020) reported that drought stress induced biosynthesis of saikosaponins in roots of *Bupleurum chinense*. Our untargeted metabolomics analysis showed that saikosaponin B4 significantly increased in WS pine roots, but significantly reduced in WS pine needles (**Table S2** and negative ion mode in **Table S10**), and that saikosaponin B2 and saikosaponin E significantly reduced in roots and needles of WS pine seedlings (**Table S10**, negative ion mode). These results suggest that saikosaponins showed differential spatial distribution in *P. taeda* and carried out different functions. Quillaic acids (quillaja saponins) are triterpenoid saponins comprising a hydrophobic quillaic acid backbone and hydrophilic sugar moieties. In roots and needles of WS pine seedlings, quillaic acids increased (**Table S2** and positive ion mode in **Table S10**). At present, no report was described about its function in plants under drought stress. Hecogenin is a steroid saponin isolated from *Agave sisalana* and *Agave salmiana*. Hecogenin glycosides (HG 1-2) increased as the increase of PEG concentrations from 0 to 20% in *Agave salmiana* plants under *in vitro* water stress conditions with PEG8000 (Puente-Garza et al., 2017). But our results showed that hecogenin levels reduced in roots and needles of WS pine seedlings (**Table S2** and positive ion mode in **Table S10**).

Reduced glutathione and ascorbate are two important antioxidants in plants and plants accumulate the two compounds under drought stress (Szarka et al., 2012; Liu et al., 2021). However, in our untargeted metabolomics analysis, reduced glutathione and ascorbate significantly reduced in roots and needles of WS pine seedlings (**Table S2**), and oxidized glutathione significantly increased in their roots and needles (**Table S10**, negative ion mode), suggesting that the conversion of oxidized glutathione to its reduced form was affected by long-term drought stress, i.e., activity of glutathione reductase reduced in P. taeda seedlings under long-term drought stress. Under long-term drought stress, their biosynthesis and conversion from their respective oxidized forms might be affected.

Betaine is an important osmoprotectant in plants under drought stress, just like proline (Mukarram et al., 2021). Betaine significantly increased in roots and needles of WS pine seedlings (**Table S2**and positive ion mode in **Table S10**). The significant increase in betaine levels is helpful for *P. taeda* seedlings under drought stress, because it can protect functional proteins, enzymes (e.g., Rubisco) and lipids of the photosynthetic apparatus, and maintain electron flow through the thylakoid membranes, and intracellular accumulation of betaine allows for water retention in the cell and prevents its dehydration (Hasan et al., 2021b; Mukarram et al., 2021). Three polyamines, i.e., diamine putrescine, triamine spermidine, and tetraamine spermine were often investigated in plants under drought stress, acting as osmoprotectants (Mukarram et al., 2021). However, in our untargeted metabolomics analysis, the three polyamines were not detected (**Table S2** and **Table S10**). The result suggests the three polyamines showed less functions in *P. taeda* seedlings under long-term drought stress. Similarly, β-cyclocitral was not detected in roots and needles of WW and WS pine seedlings (**Table S2** and **Table S10**), suggesting that the compound might be not synthesized in *P. taeda*, although the compound and its analogs showed its function in plants under drought stress (D'Alessandro et al., 2019; Havaux, 2020; Deshpande et al., 2021). β-cyclocitral function in plants under drought stress is independent of ABA (Deshpande et al., 2021), lack of β-cyclocitral in *P. taeda* implies that the tree species might not need the compound to protect itself and ABA might be more important than β-cyclocitral in *P. taeda* plants under drought stress.

Allantoin is one of the main compounds used to store and transport the nitrogen fixed in nodules, thereby it is involved in nitrogen use in legumes. Allantoin increased in roots and needles of WS pine seedlings and more allantoin was allocated in their roots, compared with WW pine seedlings (**Table S10**, negative ion mode). The result is consistent with previous research (Casartelli et al., 2019; Khan et al., 2019). Functions of allantoin in plants under drought stress are involved few aspects. (1) defense function: to reduce radical oxygen species accumulation and death of plant cells (Brychkova et al., 2008); (2) signaling function: to trigger stress responses by increasing ABA levels and expression of ABA-related genes (Watanabe et al., 2014; Takagi et al., 2016); (3) nutritional function: to circumvent allantoin degradation to ammonium as to prevent nitrogen losses (Casartelli et al., 2019). Therefore, increased levels of allantoin in WS pine seedlings can promote such three functions, further increasing drought tolerance of *P. taeda* seedlings.

### 4.7 Enriched metabolite pathways in *Pinus taeda* under drought stress

Under positive ion mode, comparing needles of WS and WW pine seedlings, the most enriched pathways included histidine metabolism, folate biosynthesis, ubiquinone and other terpenoid-quinone biosyntehsis, vitamin B6 metabolism, zeatin biosynthesis, fatty acid biosynthesis, flavone and flavonol biosynthesis (**Figure 11B** and WS_L.vs.WW_L_pos_kegg_enrichment in **Table S8**).

In the histidine metabolism pathway, urocanic acid and ergothioneine were enriched (WS_L.vs.WW_L_pos_kegg_enrichment in **Table S8**). Little has known about roles of urocanic acid in plants under environmental stresses. Recently, Xue et al. (2021) showed that urocanic acid was largely enriched in *Pinellia ternata* under shaded environment, which likely contributed to tuber quality and growth. Enrichment of urocanic acid in needles of WS pine seedlings might possess another function in plants under drought stress, i.e., acting as an antioxidant (Boo, 2020). Ergothioneine also act as an antioxidant (Kalaras et al., 2017). In folate biosynthesis pathway, sepiapterin was enriched, which acts as an antioxidant against reactive oxygen species (Ishii et al., 1999). Other enriched KEGG pathways were mainly involved in biosynthesis of fatty acids (palmitoleic acid), their functions were discussed as above. In flavone and flavonol biosynthesis pathways, syringetin was enriched and no information was known about its functions in plants under drought stress.

Comparing needles and roots of WS pine seedlings, the first five enriched pathways included tryptophan metabolism, caffeine metabolism, sesquiterpenoid and triterpenoid biosynthesis, plant hormone signal transduction, phenylalanine, tyrosine and tryptophan biosynthesis (**Figure 11C** and WS_L.vs.WW_R_pos_kegg_enrichment in **Table S8**). In tryptophan metabolism, tryptophol, melatonin, 3-indoleacetonitrile, kynurenic acid, L-kynurenine, indole-3-acetic acid, and indole were enriched. Kynurenic acid is a metabolite of tryptophan and is an endogenous antagonist of the ionotropic glutamate receptors and the α7 nicotinic acetylcholine receptor as well as an agonist of the G-protein-coupled receptor GPR35 in plants (Turski et al., 2011). Just as mentioned above, glutamate and acetylcholine act as neurotransmitters. Enriched kynurenic acid in *P. taeda* seedlings under drought stress could affect the roles of glutamate and acetylcholine, further affecting drought tolerance of *P. taeda* seedlings. Kynurenine and kynurenic acid are neuroactive compounds in plants (Yılmaz and Gökmen, 2020), maybe they function just like melatonin in plants under drought stress. Their enrichment in WS pine needles might improve drought tolerance of *P. taeda*.

Comparing roots of WS and WW pine seedlings, the most enriched pathways included metabolic pathways, zeatin biosynthesis, pentose phosphate pathway, carbapenem biosynthesis, benzoxazinoid biosynthesis, taurine and hypotaurine metabolism, butanoate metabolism (**Figure 11D** and WS_R.vs.WW_R_pos_kegg_enrichment in **Table S8**). In the pathway of taurine and hypotaurine metabolism, carbapenem biosynthesis, butanoate metabolism, porphyrin and chlorophyll metabolism, glyoxylate and dicarboxylate metabolism, and nitrogen metabolism, L-glutamic acid was enriched (WS_R.vs.WW_R_pos_kegg_enrichment in **Table S8**). Thus glutamate showed important roles in *P. taeda* seedlings under drought stress. Furthermore, glutamate acts as a neurotransmitter in plants, its receptor GLR3.7 in Arabidopsis is involved in ABA response (Chen et al., 2021). Therefore, glutamate enrichment improved drought tolerance, performing together with ABA signaling in *P. taeda* under drought stress.

Under negative ion mode, some enriched KEGG pathways were analyzed. Comparing needles of WS and WW pine seedlings, the most enriched pathways included biosynthesis of unsaturated fatty acids, arachidonic acid metabolism, plant hormone signal transduction, carotenoid biosynthesis, porphyrin and chlorophyll metabolism, flavonoid biosynthesis (**Figure S7B** and WS_L.vs.WW_L_neg_kegg_enrichment in **Table S9**). In biosynthesis of unsaturated fatty acids, eicosapentaenoic acid was enriched in needles of WS pine seedlings. Eicosapentaenoic acid is a long-chain fatty acid (20:5), five unsaturated double bonds can better maintain membrane flexibility in plants under drought stress, furthermore maintaining cell function. In the pathways of plant hormone signal transduction and carotenoid biosynthesis, ABA was enriched in WS pine needles (**Figure S7B** and WS_L.vs.WW_L_neg_kegg_enrichment in **Table S9**). In flavonoid biosynthesis, neohesperidin was enriched in WS pine needles. Neohesperidin has free radical scavenging activity (Xu et al., 2012), thus its enrichment in WS pine needles improves scavenging of reactive oxygen species and maintain functions of macromolcules in plant cells under drought stress. In porphyrin and chlorophyll metabolism, bilirubin was enriched in WS pine needles. Bilirubin shows multiple physiological functions, such as antioxidant effects, generation of reactive oxygen species, and cell signaling (Vítek and Ostrow, 2009). Maybe it also showed these functions in plants under drought stress.

Comparing needles and roots of WS pine seedlings, the most enriched pathways included phenylalanine metabolism, tyrosine metabolism, butanoate metabolism (**Figure S7B** and WS_L.vs.WS_R_neg_kegg_enrichment in **Table S9**). In the three pathways, fumaric acid was the most enriched metabolite in needles of WS pine seedlings. But targeted metabolics analysis showed that level of fumaric acid was significantly lower in needles of WS pine seedlings than those in their roots (**Figure 4C**). Fumaric acid is a component of the tricarboxylic acid cycle and can be transformed to yield energy and carbon skeletons for other compounds. Previous research showed that fumaric acid levels increased with plant age and light intensity in Arabidopsis leaves and that higher levels were determined in Arabidopsis phloem, suggesting the possibility that fumaric acid may function in carbon transport (Chia et al., 2000). Under drought stress, the possibility might increase because levels of fumaric acid significantly increased in WS pine roots (**Figure 3C**).

Comparing roots of WS and WW pine seedlings, the most enriched pathways included arginine and proline metabolism, lysine degradation, tryptophan metabolism, biosynthesis of unsaturate fatty acids, biosyntehsis of amino acids (**Figure S7B** and WS_R.vs.WW_R_neg_kegg_enrichment in **Table S9**). In arginine and proline metabolism, D-proline and 4-oxoproline were enriched in WS pine roots. It is very clear about role of proline in plants under drought stress (as mentioned above), but little has been known about 4-oxoproline. In the five pathways, i.e., lysine biosynthesis and degradation, biosynthesis of amino acids, 2-oxocarboxylic acid metabolism, and tryptophan metabolism, 2-oxoadipic acid was enriched in WS pine roots (WS_R.vs.WW_R_neg_kegg_enrichment in **Table S9**). 2-oxoadipic acid is a key metabolite of tryptophan and lysine. Enrichment of 2-oxoadipic acid in WS pine roots suggested that the acid was strengthened in metabolism of tryptophan and lysine and biosynthesis of amino acids in plants under drought stress. In biosynthesis of amino acids, L-citrulline was enriched in WS pine roots. In few plant species, citrulline showed a significant amount in their phloem exudates (Joshi and Fernie, 2017). Previous research showed that the genes involved in the citrulline biosynthesis significantly enriched in plants under drought stress (Gong et al., 2010; Shaik and Ramakrishna, 2012; Liu et al., 2015; Garg et al., 2016; Borah et al., 2017), suggesting a universal role of citrulline as a compatible solute in response to drought stress.

## Data availability statement

The original contributions presented in the study are included in the article/**Supplementary materials**. Further inquiries can be directed to the corresponding author.

## Author contributions

CW planned the all experiments and wrote the rough draft of the article. YW and CW carried out targeted analyses. HS financially supported the research in part and reviewed and revised the rough draft of the article. All authors contributed to the article and approved the submitted version.

## Supplementary material

Supplementary Figure 1 PLS-DA scores of experimental groups under negative ion mode. **(A)** needles (red) vs. roots (blue) of WW seedlings; **(B)** needles of WS (red) vs.WW (blue) pine seedlings; **(C)** needles (red) vs. roots (blue) of WS pine seedlings; **(D)** roots of WS (red) vs. WW (blue) pine seedlings. WW, well-watered; WS, water-stressed.

Supplementary Figure 2 PCA analysis under positive and negative ion mode. **(A–D)** positive ion mode. **(A)** needles (red) vs. roots (blue) of WW seedlings; **(B)** needles of WS (red) vs.WW (blue) pine seedlings; **(C)** needles (red) vs. roots (blue) of WS pine seedlings; **(D)** roots of WS (red) vs. WW (blue) pine seedlings. **(E–H)**: negative ion mode. **(E)** needles (red) vs. roots (blue) of WW seedlings; **(F)** needles of WS (red) vs.WW (blue) pine seedlings; **(G)** needles (red) vs. roots (blue) of WS pine seedlings; **(H)** roots of WS (red) vs. WW (blue) pine seedlings. WW, well-watered; WS, water-stressed.

Supplementary Figure 3 Hierarchical clustering analysis of all differential metabolites from the four groups of samples (negative ion mode). L and R indicated the leaves and roots of *P. taeda* seedlings under WW and WS conndition, respectively. WW, well-watered; WS, water-stressed.

Supplementary Figure 4 Heatmaps of differential metabolites. **(A, B)** needles vs. roots of WW seedlings, positive and negative ion mode, respectively; **(C, D)** needles of WS vs.WW pine seedlings, positive and negative ion mode, respectively; **(E, F)** needles vs. roots of WS pine seedlings, positive and negative ion mode, respectively; **(G, H)** roots of WS vs. WW pine seedlings, positive and negative ion mode, respectively.

Supplementary Figure 5 Correlation analysis of metabolites (negative ion mode) with *p* values of top 20 (beginning from the minimal p value). The highest correlation with correlation coefficient of 1 was represented in red; the lowest correlation with correlation coefficient of -1 was represented in blue. Colorless parts indicated *p* value > 0.05. A: WW_L vs. WW_R; B: WS_L vs. WW_L; B: WS_L vs. WS_R; D: WS_R vs. WW_R. L and R indicated the leaves and roots of *P. taeda* seedlings under WW and WS conndition, respectively. WW, well-watered; WS, water-stressed.

Supplementary Figure 6 Z score analysis of the four sample groups under negative ion mode. A: WW_L vs. WW_R; B: WS_L vs. WW_L; B: WS_L vs. WS_R; B: WS_R vs. WW_R. L and R indicated the leaves and roots of *P. taeda* seedlings under WW and WS conndition, respectively. WW, well-watered; WS, water-stressed.

Supplementary Figure 7 Metabolites enriched in KEGG pathways under positive ion mode (top 20 pathways). In the small figures, horizontal axis represented the ratio of number of differential metabolites to the total numbers of metabolites identified in a pathway. Greater the ratio was, higher the differential metabolites enriched in the pathway. Color of a circle represented *p* value in geometric test. The size of a circle represented the number of differential metabolites in the corresponding pathway. A: WW_L vs. WW_R; B: WS_L vs. WW_L; B: WS_L vs. WS_R; D: WS_R vs. WW_R. L and R indicated the leaves and roots of *P. taeda* seedlings under WW and WS conndition, respectively. WW, well-watered; WS, water-stressed.

Supplementary Table 1 Relatively quantitative levels of identified metabolites in all the samples.

Supplementary Table 2 Annotations of differential metabolites under positive ion mode.

Supplementary Table 3 Annotations of differential metabolites under negative ion mode.

Supplementary Table 4 Differential metabolite analysis–Venn analysis.

Supplementary Table 5 Correlation analysis of differential metabolites under positive ion mode.

Supplementary Table 6 Correlation analysis of differential metabolites under negative ion mode.

Supplementary Table 7 Z-score analysis of differential metabolites.

Supplementary Table 8 KEGG enrichment analysis of differential metabolites under positive ion mode.

Supplementary Table 9 KEGG enrichment analysis of differential metabolites under negative ion mode.

Supplementary Table 10 Heatmaps of differential metabolites.
