# Supplementary figures and images for: Targeted and untargeted metabolomics reveals deep analysis of drought stress responses in needles and roots of Pinus taeda seedlings
Source: Front Plant Sci. 2023 Jan 31;13:1031466. doi: 10.3389/fpls.2022.1031466 (PMC9927248; doi:10.3389/fpls.2022.1031466)

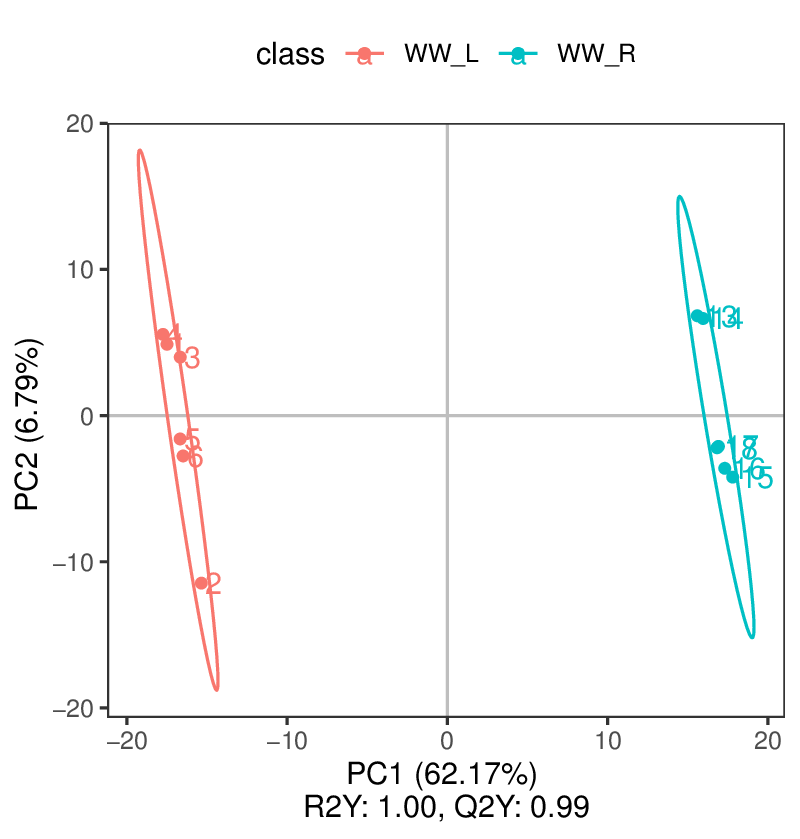

Supplement: Supplementary Figure 1 — PLS-DA scores of experimental groups under negative ion mode. (A) needles (red) vs. roots (blue) of WW seedlings; (B) needles of WS (red) vs.WW (blue) pine seedlings; (C) needles (red) vs. roots (blue) of WS pine seedlings; (D) roots of WS (red) vs. WW (blue) pine seedlings. WW, well-watered; WS, water-stressed. [file DataSheet_1.zip › Supplementary Figures/Fig. S1/Fig. S1A.png]

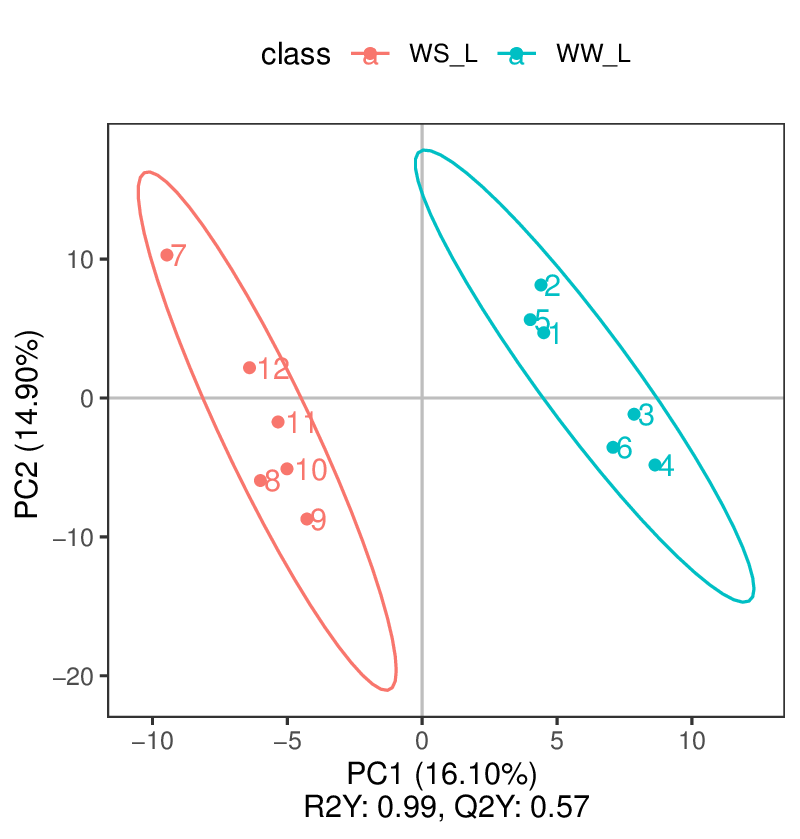

Supplement: Supplementary Figure 1 — PLS-DA scores of experimental groups under negative ion mode. (A) needles (red) vs. roots (blue) of WW seedlings; (B) needles of WS (red) vs.WW (blue) pine seedlings; (C) needles (red) vs. roots (blue) of WS pine seedlings; (D) roots of WS (red) vs. WW (blue) pine seedlings. WW, well-watered; WS, water-stressed. [file DataSheet_1.zip › Supplementary Figures/Fig. S1/Fig. S1B.png]

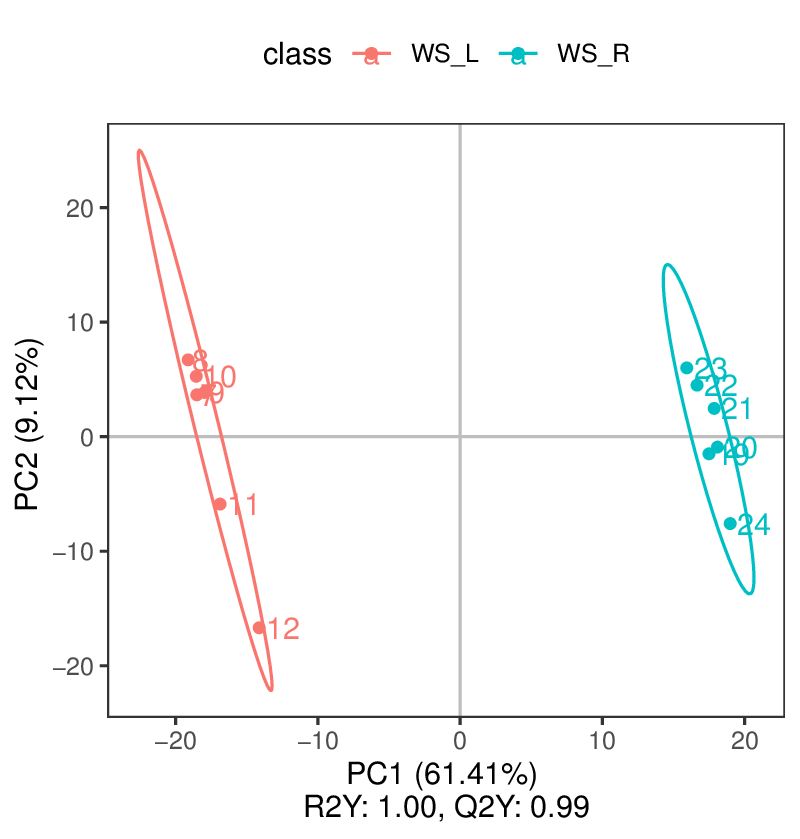

Supplement: Supplementary Figure 1 — PLS-DA scores of experimental groups under negative ion mode. (A) needles (red) vs. roots (blue) of WW seedlings; (B) needles of WS (red) vs.WW (blue) pine seedlings; (C) needles (red) vs. roots (blue) of WS pine seedlings; (D) roots of WS (red) vs. WW (blue) pine seedlings. WW, well-watered; WS, water-stressed. [file DataSheet_1.zip › Supplementary Figures/Fig. S1/Fig. S1C.png]

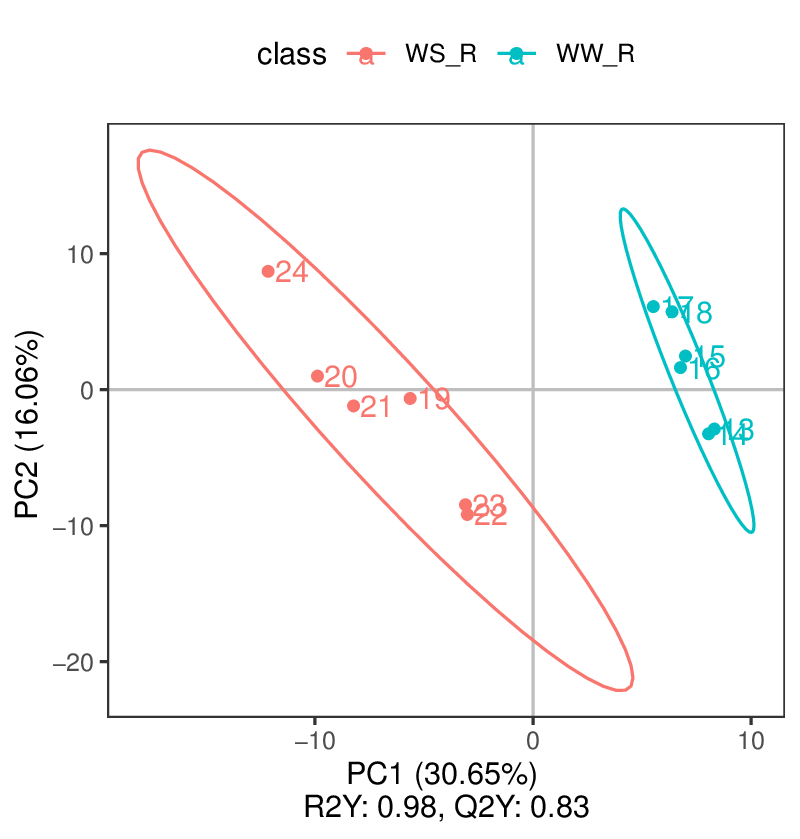

Supplement: Supplementary Figure 1 — PLS-DA scores of experimental groups under negative ion mode. (A) needles (red) vs. roots (blue) of WW seedlings; (B) needles of WS (red) vs.WW (blue) pine seedlings; (C) needles (red) vs. roots (blue) of WS pine seedlings; (D) roots of WS (red) vs. WW (blue) pine seedlings. WW, well-watered; WS, water-stressed. [file DataSheet_1.zip › Supplementary Figures/Fig. S1/Fig. S1D.png]

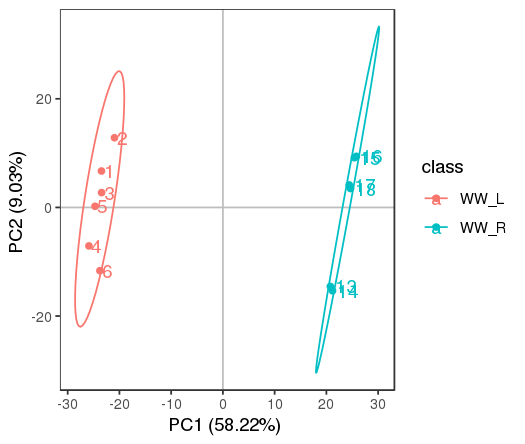

Supplement: Supplementary Figure 1 — PLS-DA scores of experimental groups under negative ion mode. (A) needles (red) vs. roots (blue) of WW seedlings; (B) needles of WS (red) vs.WW (blue) pine seedlings; (C) needles (red) vs. roots (blue) of WS pine seedlings; (D) roots of WS (red) vs. WW (blue) pine seedlings. WW, well-watered; WS, water-stressed. [file DataSheet_1.zip › Supplementary Figures/Fig. S2/Fig. S2A.png]

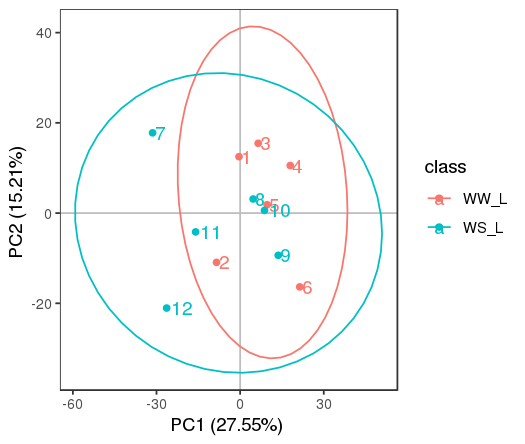

Supplement: Supplementary Figure 1 — PLS-DA scores of experimental groups under negative ion mode. (A) needles (red) vs. roots (blue) of WW seedlings; (B) needles of WS (red) vs.WW (blue) pine seedlings; (C) needles (red) vs. roots (blue) of WS pine seedlings; (D) roots of WS (red) vs. WW (blue) pine seedlings. WW, well-watered; WS, water-stressed. [file DataSheet_1.zip › Supplementary Figures/Fig. S2/Fig. S2B.png]

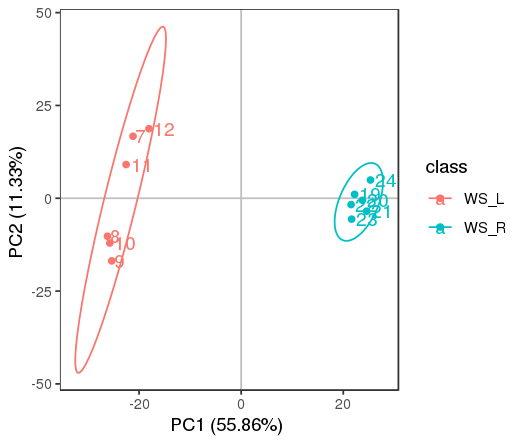

Supplement: Supplementary Figure 1 — PLS-DA scores of experimental groups under negative ion mode. (A) needles (red) vs. roots (blue) of WW seedlings; (B) needles of WS (red) vs.WW (blue) pine seedlings; (C) needles (red) vs. roots (blue) of WS pine seedlings; (D) roots of WS (red) vs. WW (blue) pine seedlings. WW, well-watered; WS, water-stressed. [file DataSheet_1.zip › Supplementary Figures/Fig. S2/Fig. S2C.png]

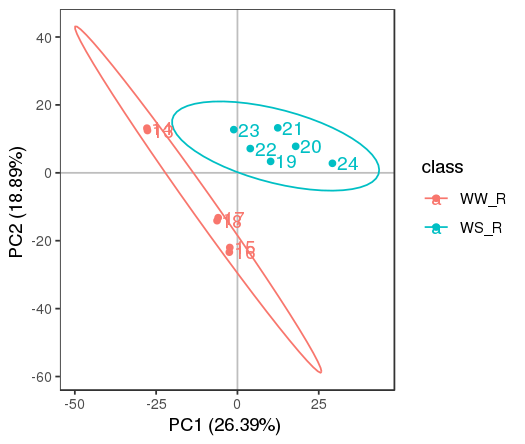

Supplement: Supplementary Figure 1 — PLS-DA scores of experimental groups under negative ion mode. (A) needles (red) vs. roots (blue) of WW seedlings; (B) needles of WS (red) vs.WW (blue) pine seedlings; (C) needles (red) vs. roots (blue) of WS pine seedlings; (D) roots of WS (red) vs. WW (blue) pine seedlings. WW, well-watered; WS, water-stressed. [file DataSheet_1.zip › Supplementary Figures/Fig. S2/Fig. S2D.png]

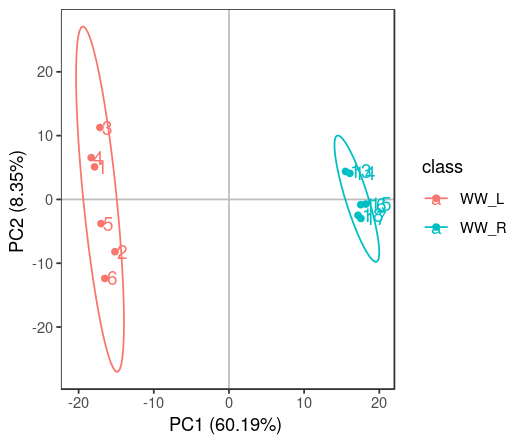

Supplement: Supplementary Figure 1 — PLS-DA scores of experimental groups under negative ion mode. (A) needles (red) vs. roots (blue) of WW seedlings; (B) needles of WS (red) vs.WW (blue) pine seedlings; (C) needles (red) vs. roots (blue) of WS pine seedlings; (D) roots of WS (red) vs. WW (blue) pine seedlings. WW, well-watered; WS, water-stressed. [file DataSheet_1.zip › Supplementary Figures/Fig. S2/Fig. S2E.png]

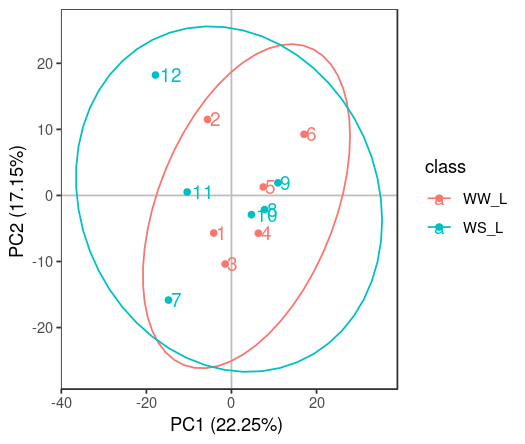

Supplement: Supplementary Figure 1 — PLS-DA scores of experimental groups under negative ion mode. (A) needles (red) vs. roots (blue) of WW seedlings; (B) needles of WS (red) vs.WW (blue) pine seedlings; (C) needles (red) vs. roots (blue) of WS pine seedlings; (D) roots of WS (red) vs. WW (blue) pine seedlings. WW, well-watered; WS, water-stressed. [file DataSheet_1.zip › Supplementary Figures/Fig. S2/Fig. S2F.png]

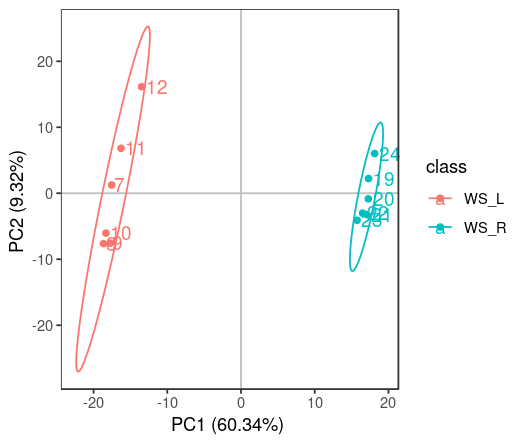

Supplement: Supplementary Figure 1 — PLS-DA scores of experimental groups under negative ion mode. (A) needles (red) vs. roots (blue) of WW seedlings; (B) needles of WS (red) vs.WW (blue) pine seedlings; (C) needles (red) vs. roots (blue) of WS pine seedlings; (D) roots of WS (red) vs. WW (blue) pine seedlings. WW, well-watered; WS, water-stressed. [file DataSheet_1.zip › Supplementary Figures/Fig. S2/Fig. S2G.png]

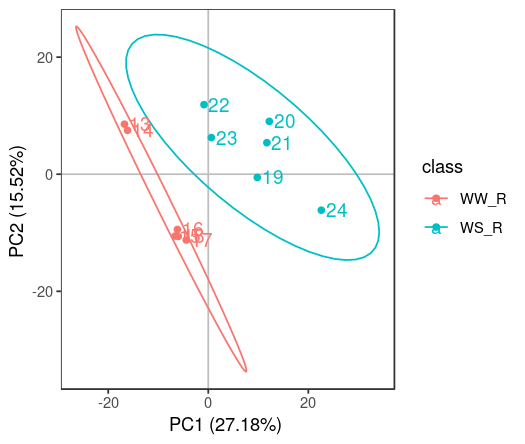

Supplement: Supplementary Figure 1 — PLS-DA scores of experimental groups under negative ion mode. (A) needles (red) vs. roots (blue) of WW seedlings; (B) needles of WS (red) vs.WW (blue) pine seedlings; (C) needles (red) vs. roots (blue) of WS pine seedlings; (D) roots of WS (red) vs. WW (blue) pine seedlings. WW, well-watered; WS, water-stressed. [file DataSheet_1.zip › Supplementary Figures/Fig. S2/Fig. S2H.png]

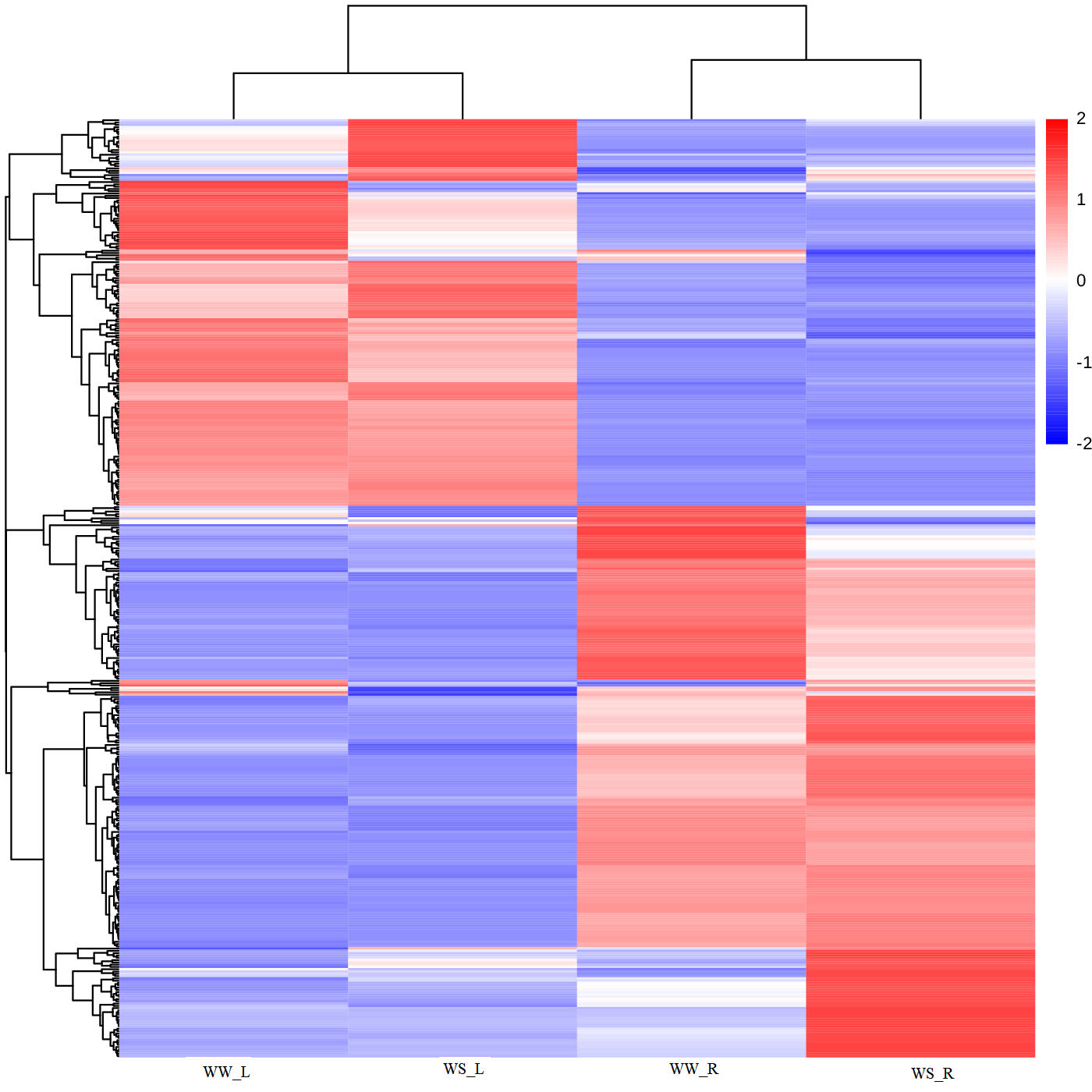

Supplement: Supplementary Figure 1 — PLS-DA scores of experimental groups under negative ion mode. (A) needles (red) vs. roots (blue) of WW seedlings; (B) needles of WS (red) vs.WW (blue) pine seedlings; (C) needles (red) vs. roots (blue) of WS pine seedlings; (D) roots of WS (red) vs. WW (blue) pine seedlings. WW, well-watered; WS, water-stressed. [file DataSheet_1.zip › Supplementary Figures/Fig. S3/Fig. S3--Diff_Heatmap_neg.cluster.png]

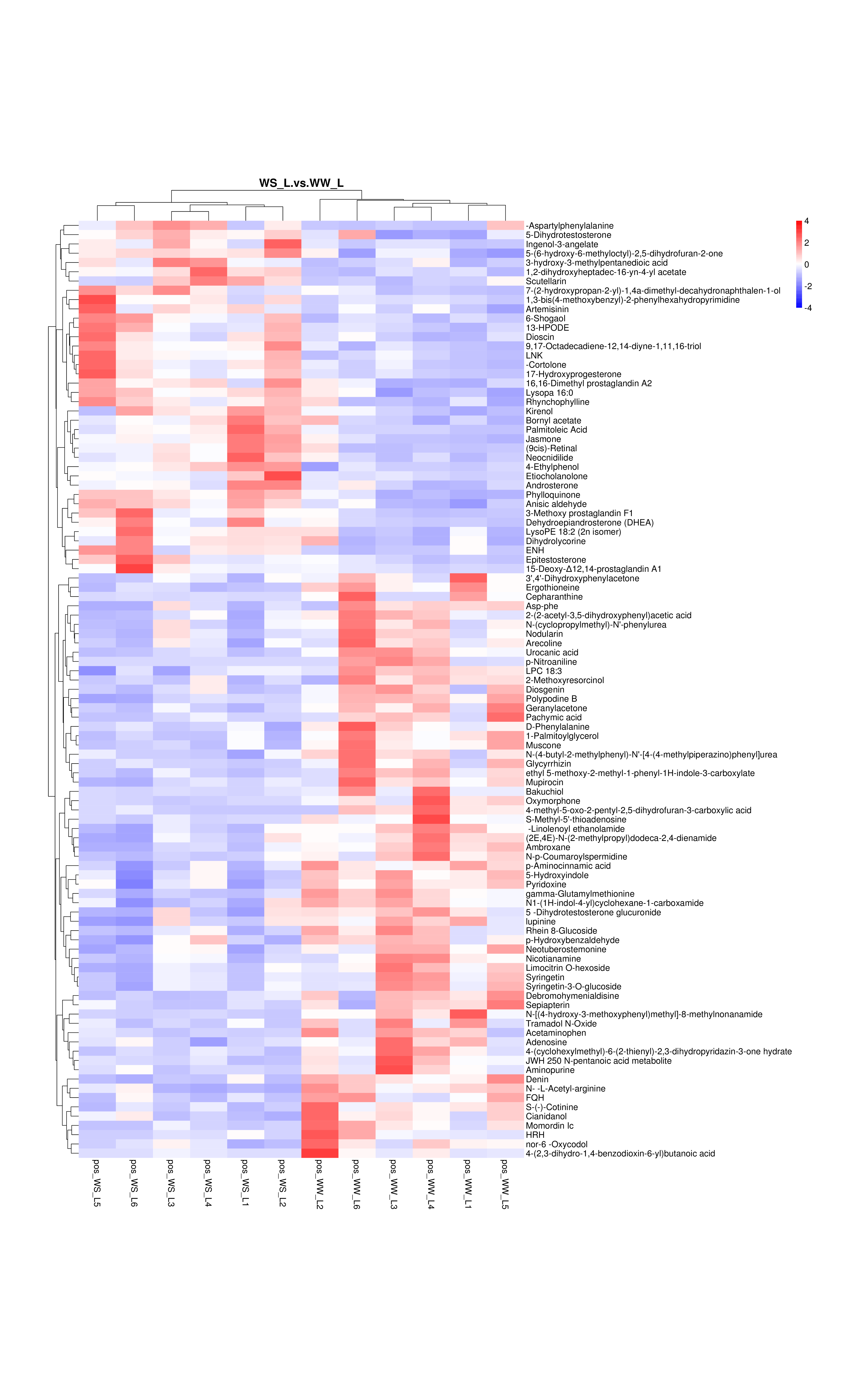

Supplement: Supplementary Figure 1 — PLS-DA scores of experimental groups under negative ion mode. (A) needles (red) vs. roots (blue) of WW seedlings; (B) needles of WS (red) vs.WW (blue) pine seedlings; (C) needles (red) vs. roots (blue) of WS pine seedlings; (D) roots of WS (red) vs. WW (blue) pine seedlings. WW, well-watered; WS, water-stressed. [file DataSheet_1.zip › Supplementary Figures/Fig. S4/Fig. S4C--WS_L.vs.WW_L_pos_cluster_heatmap_detail.png]

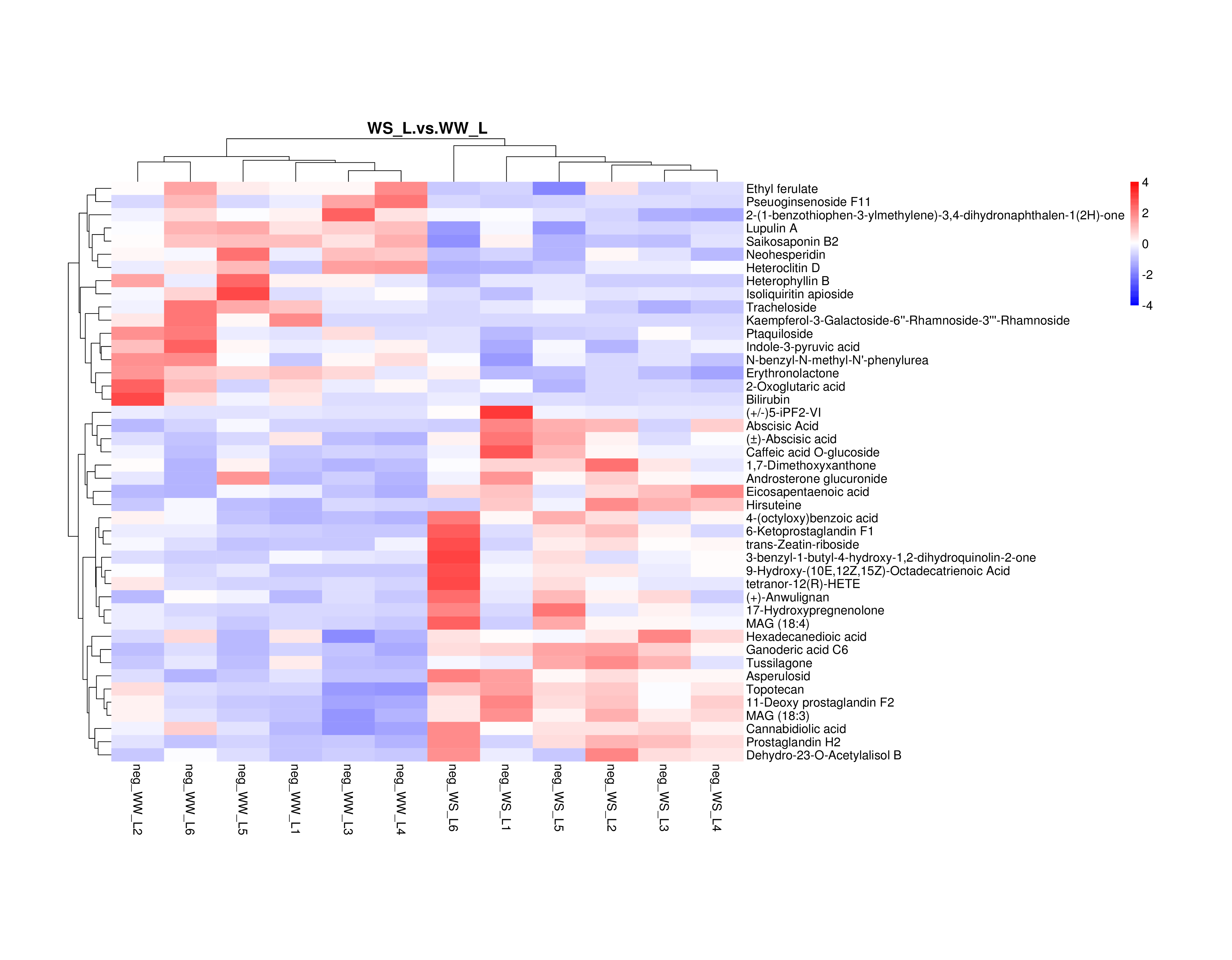

Supplement: Supplementary Figure 1 — PLS-DA scores of experimental groups under negative ion mode. (A) needles (red) vs. roots (blue) of WW seedlings; (B) needles of WS (red) vs.WW (blue) pine seedlings; (C) needles (red) vs. roots (blue) of WS pine seedlings; (D) roots of WS (red) vs. WW (blue) pine seedlings. WW, well-watered; WS, water-stressed. [file DataSheet_1.zip › Supplementary Figures/Fig. S4/Fig. S4D--WS_L.vs.WW_L_neg_cluster_heatmap_detail.png]

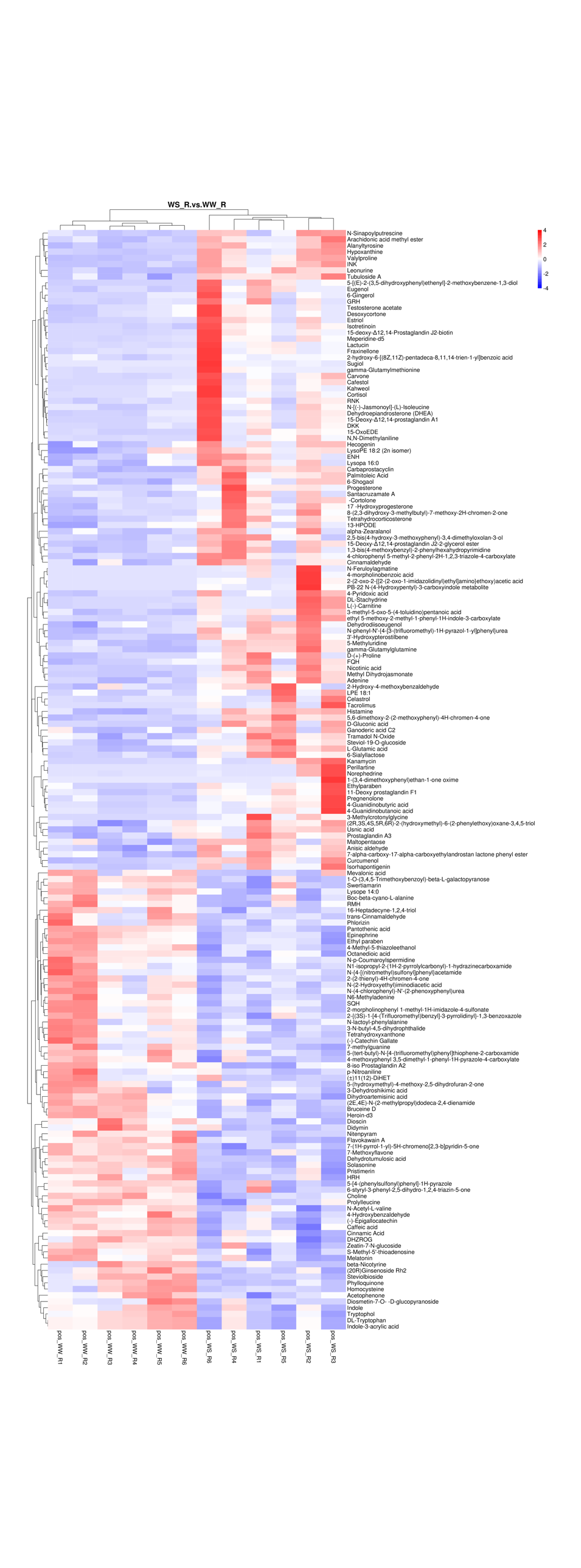

Supplement: Supplementary Figure 1 — PLS-DA scores of experimental groups under negative ion mode. (A) needles (red) vs. roots (blue) of WW seedlings; (B) needles of WS (red) vs.WW (blue) pine seedlings; (C) needles (red) vs. roots (blue) of WS pine seedlings; (D) roots of WS (red) vs. WW (blue) pine seedlings. WW, well-watered; WS, water-stressed. [file DataSheet_1.zip › Supplementary Figures/Fig. S4/Fig. S4G--WS_R.vs.WW_R_pos_cluster_heatmap_detail.png]

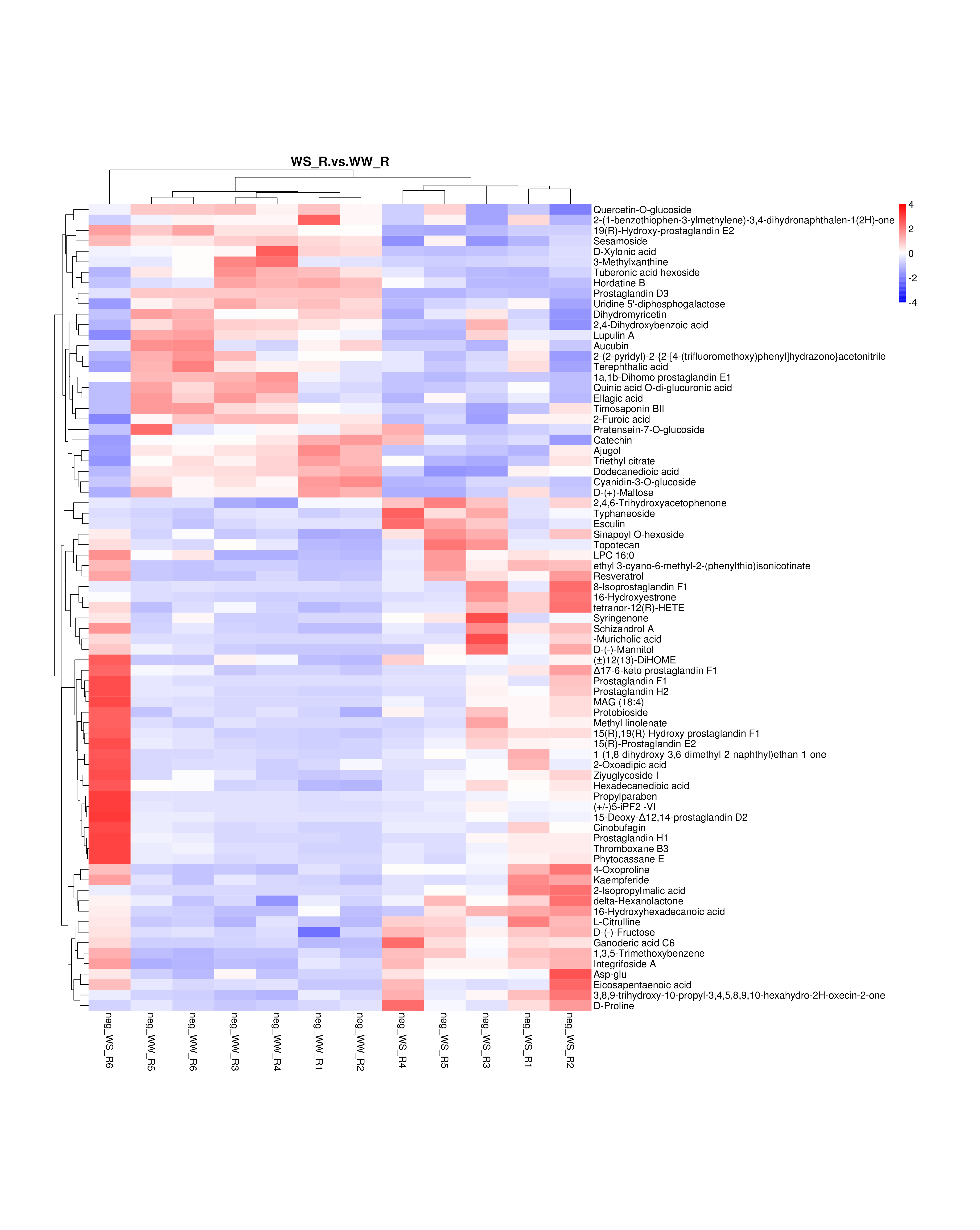

Supplement: Supplementary Figure 1 — PLS-DA scores of experimental groups under negative ion mode. (A) needles (red) vs. roots (blue) of WW seedlings; (B) needles of WS (red) vs.WW (blue) pine seedlings; (C) needles (red) vs. roots (blue) of WS pine seedlings; (D) roots of WS (red) vs. WW (blue) pine seedlings. WW, well-watered; WS, water-stressed. [file DataSheet_1.zip › Supplementary Figures/Fig. S4/Fig. S4H--WS_R.vs.WW_R_neg_cluster_heatmap_detail.png]

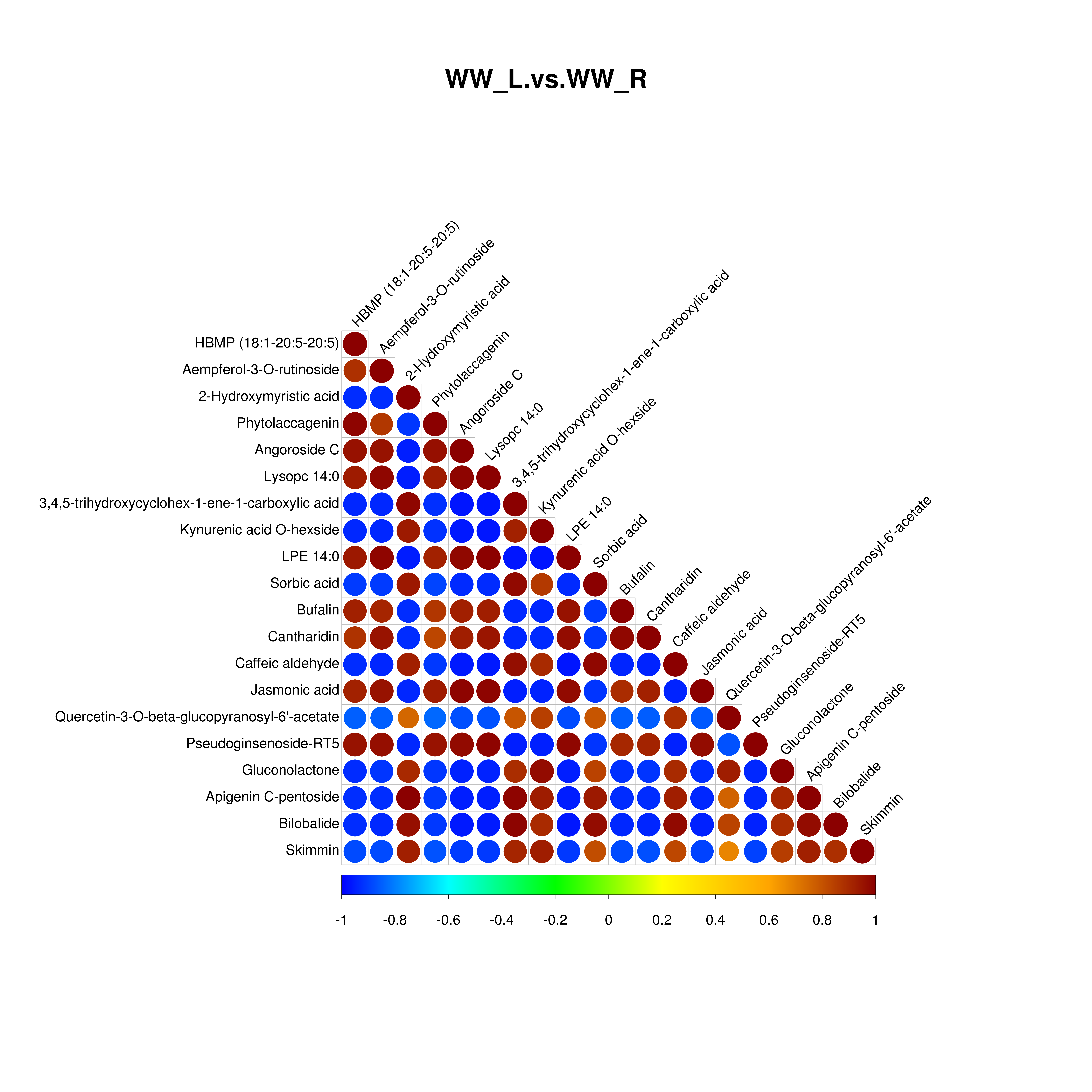

Supplement: Supplementary Figure 1 — PLS-DA scores of experimental groups under negative ion mode. (A) needles (red) vs. roots (blue) of WW seedlings; (B) needles of WS (red) vs.WW (blue) pine seedlings; (C) needles (red) vs. roots (blue) of WS pine seedlings; (D) roots of WS (red) vs. WW (blue) pine seedlings. WW, well-watered; WS, water-stressed. [file DataSheet_1.zip › Supplementary Figures/Fig. S5/Fig. S5A--WW_L.vs.WW_R_neg_corr.png]

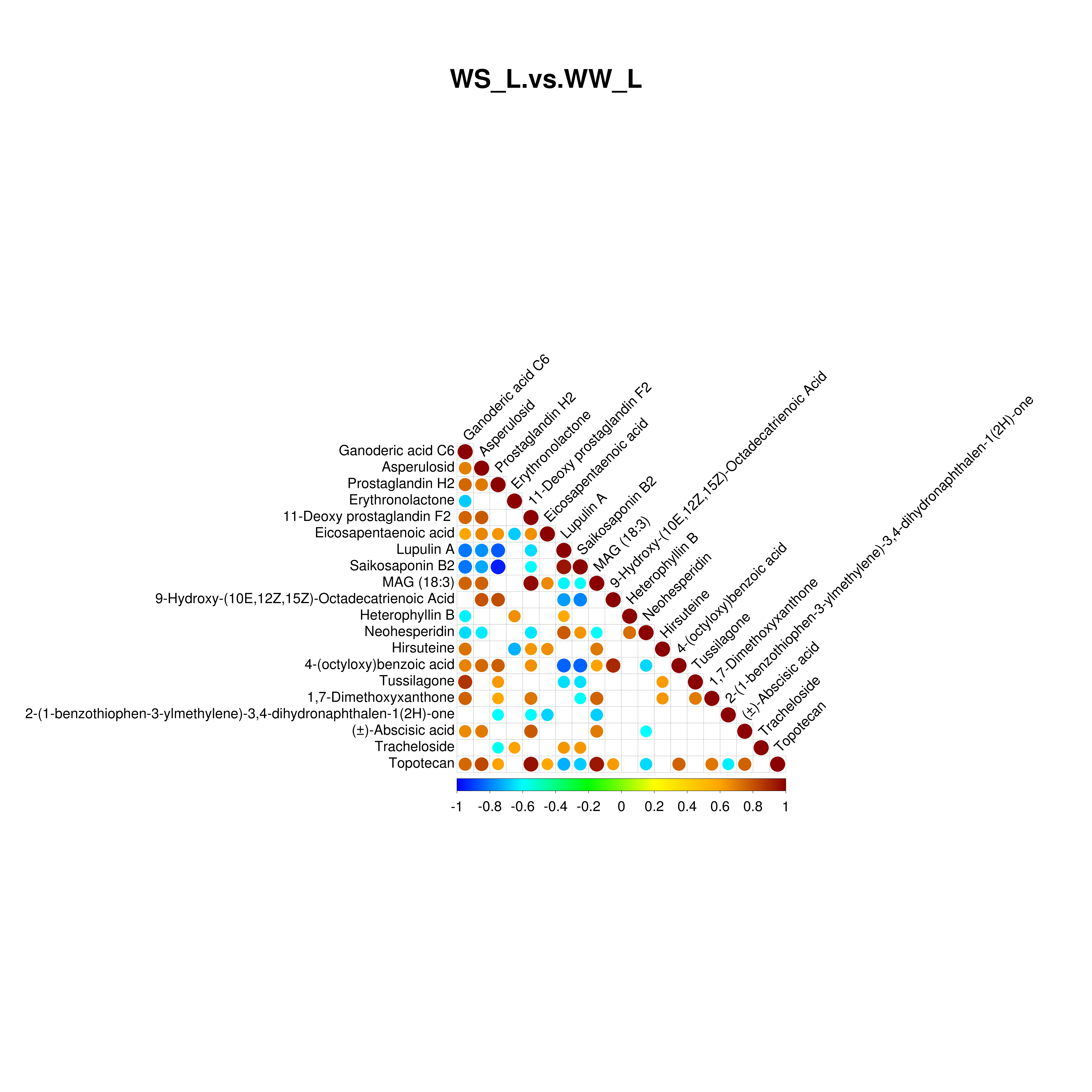

Supplement: Supplementary Figure 1 — PLS-DA scores of experimental groups under negative ion mode. (A) needles (red) vs. roots (blue) of WW seedlings; (B) needles of WS (red) vs.WW (blue) pine seedlings; (C) needles (red) vs. roots (blue) of WS pine seedlings; (D) roots of WS (red) vs. WW (blue) pine seedlings. WW, well-watered; WS, water-stressed. [file DataSheet_1.zip › Supplementary Figures/Fig. S5/Fig. S5B--WS_L.vs.WW_L_neg_corr.png]

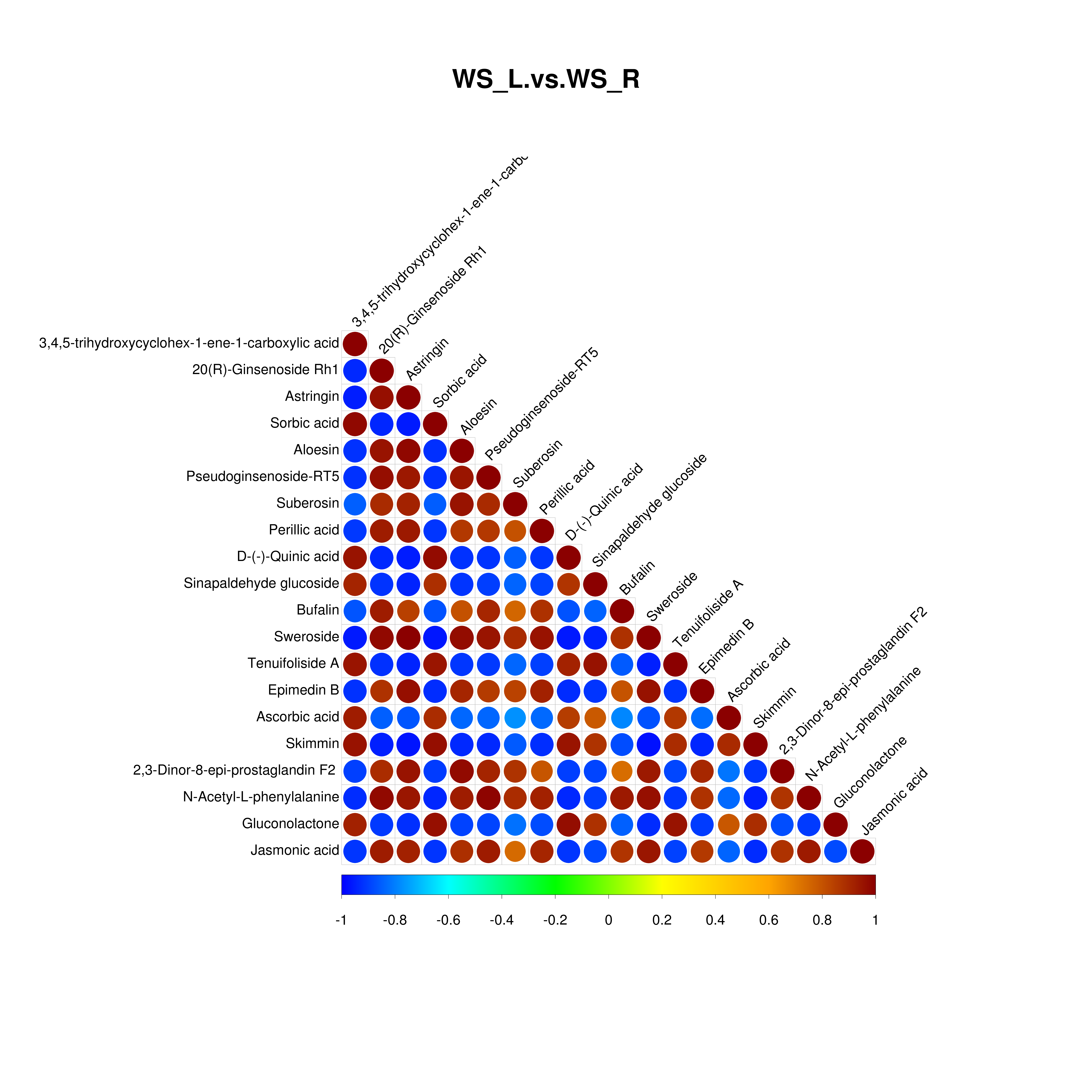

Supplement: Supplementary Figure 1 — PLS-DA scores of experimental groups under negative ion mode. (A) needles (red) vs. roots (blue) of WW seedlings; (B) needles of WS (red) vs.WW (blue) pine seedlings; (C) needles (red) vs. roots (blue) of WS pine seedlings; (D) roots of WS (red) vs. WW (blue) pine seedlings. WW, well-watered; WS, water-stressed. [file DataSheet_1.zip › Supplementary Figures/Fig. S5/Fig. S5C--WS_L.vs.WS_R_neg_corr.png]

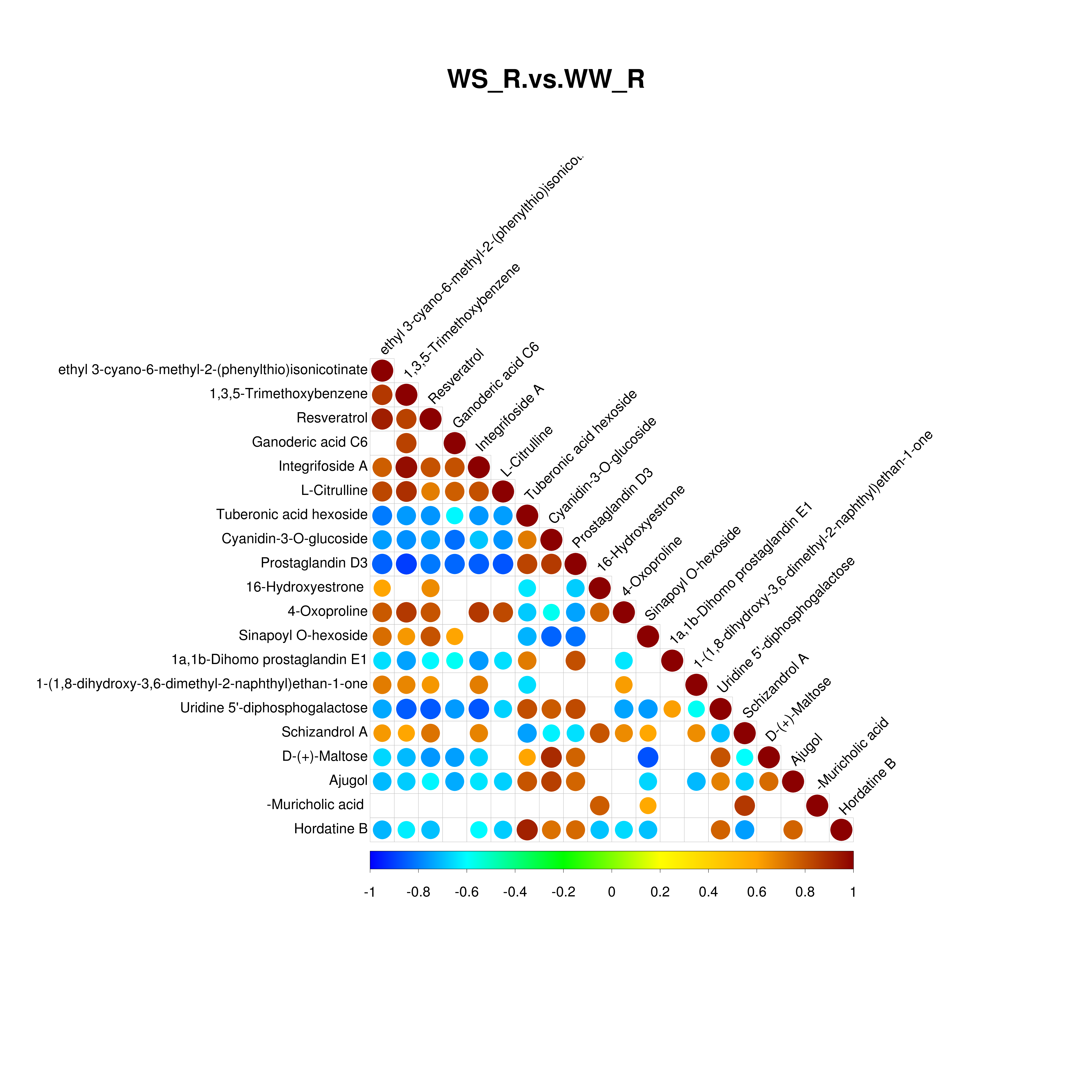

Supplement: Supplementary Figure 1 — PLS-DA scores of experimental groups under negative ion mode. (A) needles (red) vs. roots (blue) of WW seedlings; (B) needles of WS (red) vs.WW (blue) pine seedlings; (C) needles (red) vs. roots (blue) of WS pine seedlings; (D) roots of WS (red) vs. WW (blue) pine seedlings. WW, well-watered; WS, water-stressed. [file DataSheet_1.zip › Supplementary Figures/Fig. S5/Fig. S5-D--WS_R.vs.WW_R_neg_corr.png]

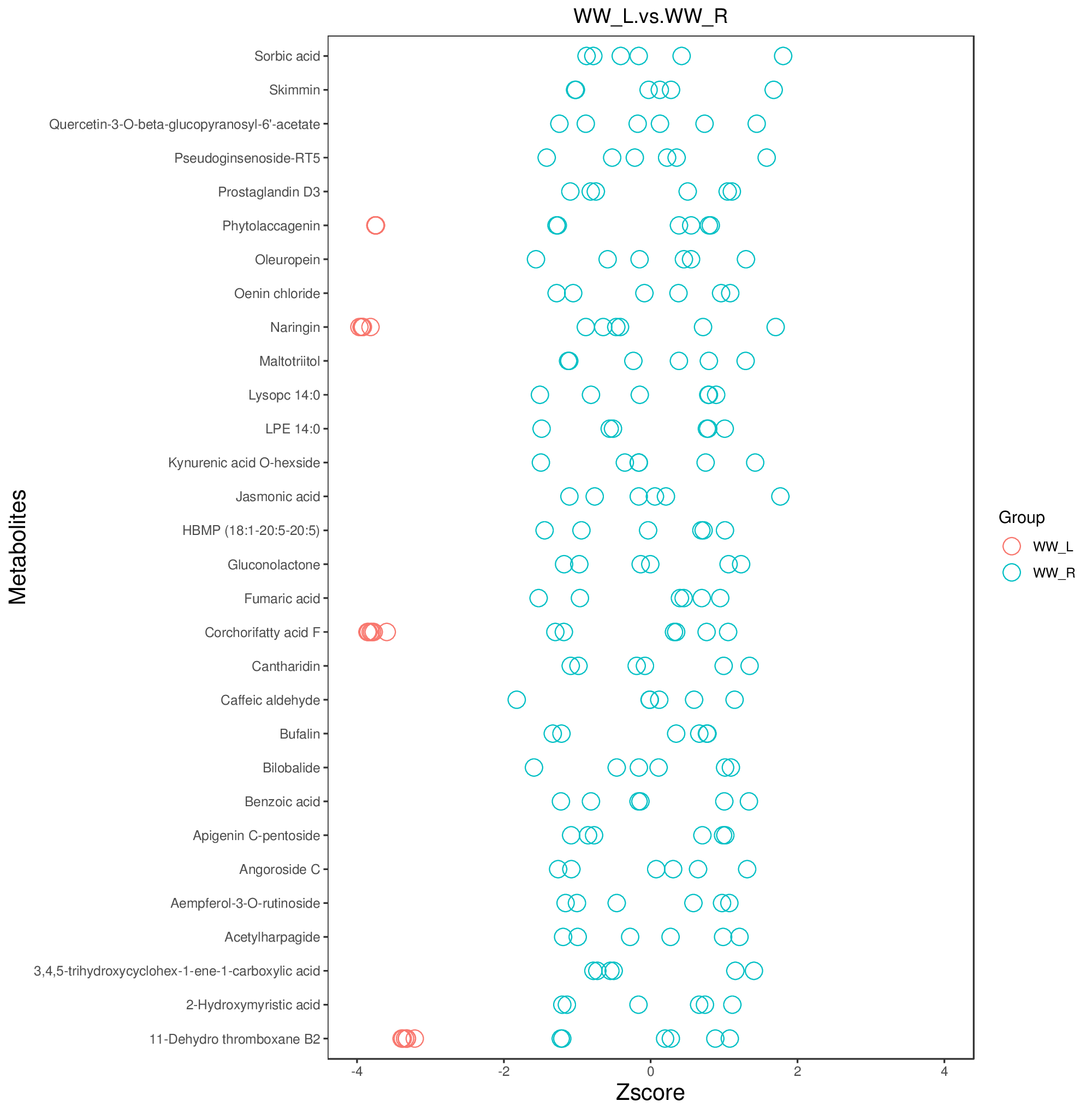

Supplement: Supplementary Figure 1 — PLS-DA scores of experimental groups under negative ion mode. (A) needles (red) vs. roots (blue) of WW seedlings; (B) needles of WS (red) vs.WW (blue) pine seedlings; (C) needles (red) vs. roots (blue) of WS pine seedlings; (D) roots of WS (red) vs. WW (blue) pine seedlings. WW, well-watered; WS, water-stressed. [file DataSheet_1.zip › Supplementary Figures/Fig. S6/Fig. S6A--WW_L.vs.WW_R_neg_zscore.png]

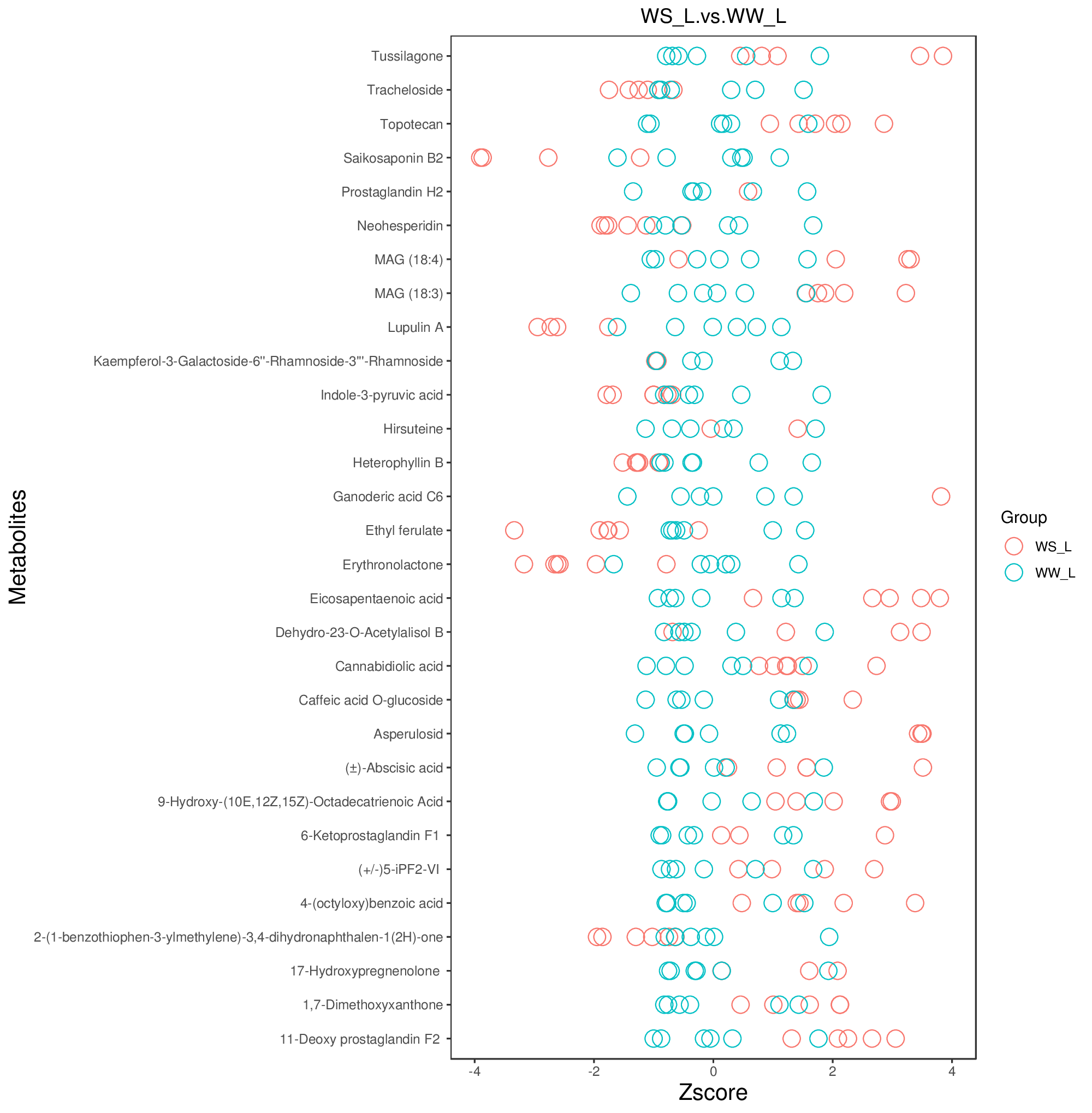

Supplement: Supplementary Figure 1 — PLS-DA scores of experimental groups under negative ion mode. (A) needles (red) vs. roots (blue) of WW seedlings; (B) needles of WS (red) vs.WW (blue) pine seedlings; (C) needles (red) vs. roots (blue) of WS pine seedlings; (D) roots of WS (red) vs. WW (blue) pine seedlings. WW, well-watered; WS, water-stressed. [file DataSheet_1.zip › Supplementary Figures/Fig. S6/Fig. S6B--WS_L.vs.WW_L_neg_zscore.png]

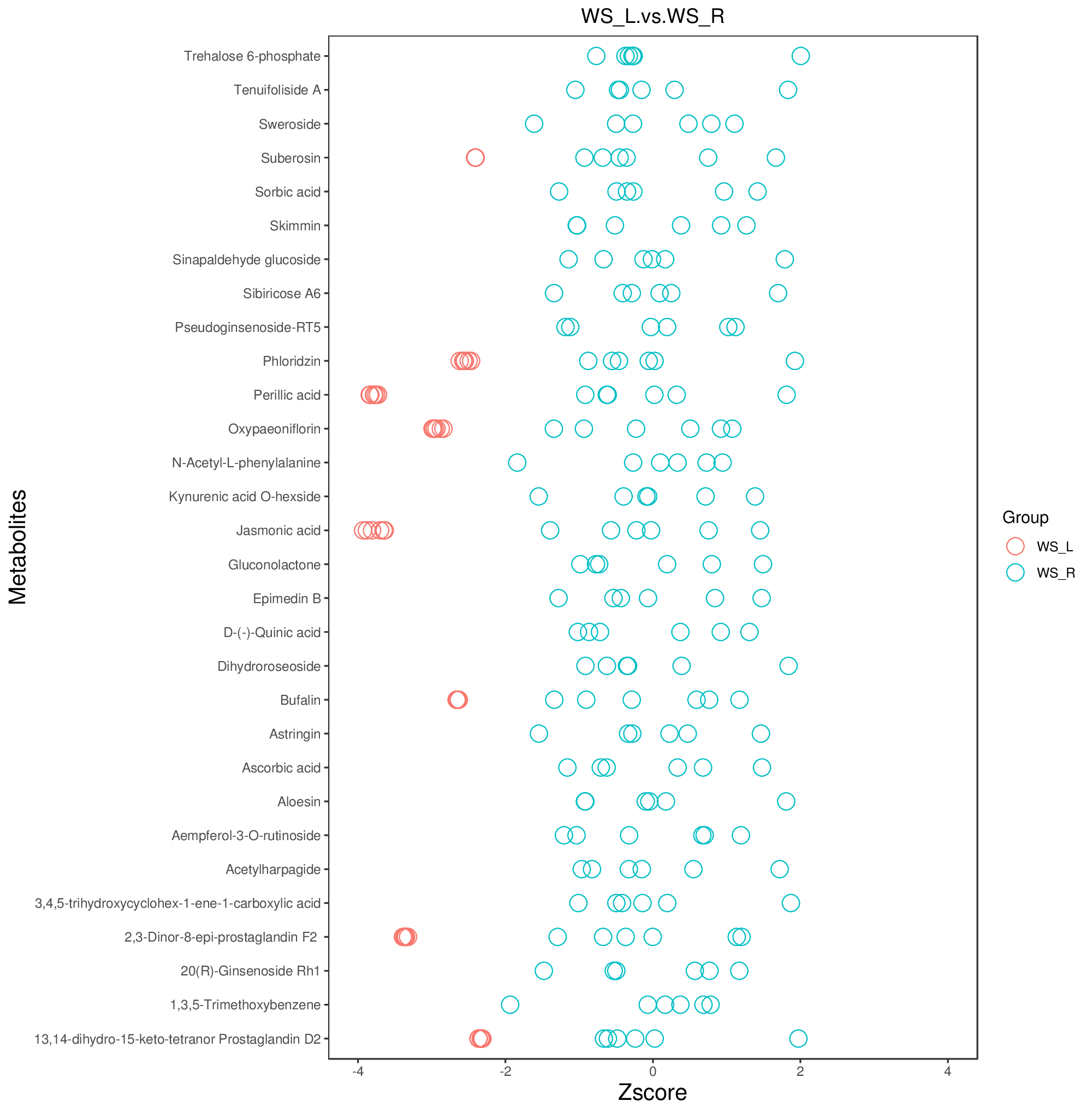

Supplement: Supplementary Figure 1 — PLS-DA scores of experimental groups under negative ion mode. (A) needles (red) vs. roots (blue) of WW seedlings; (B) needles of WS (red) vs.WW (blue) pine seedlings; (C) needles (red) vs. roots (blue) of WS pine seedlings; (D) roots of WS (red) vs. WW (blue) pine seedlings. WW, well-watered; WS, water-stressed. [file DataSheet_1.zip › Supplementary Figures/Fig. S6/Fig. S6C--WS_L.vs.WS_R_neg_zscore.png]

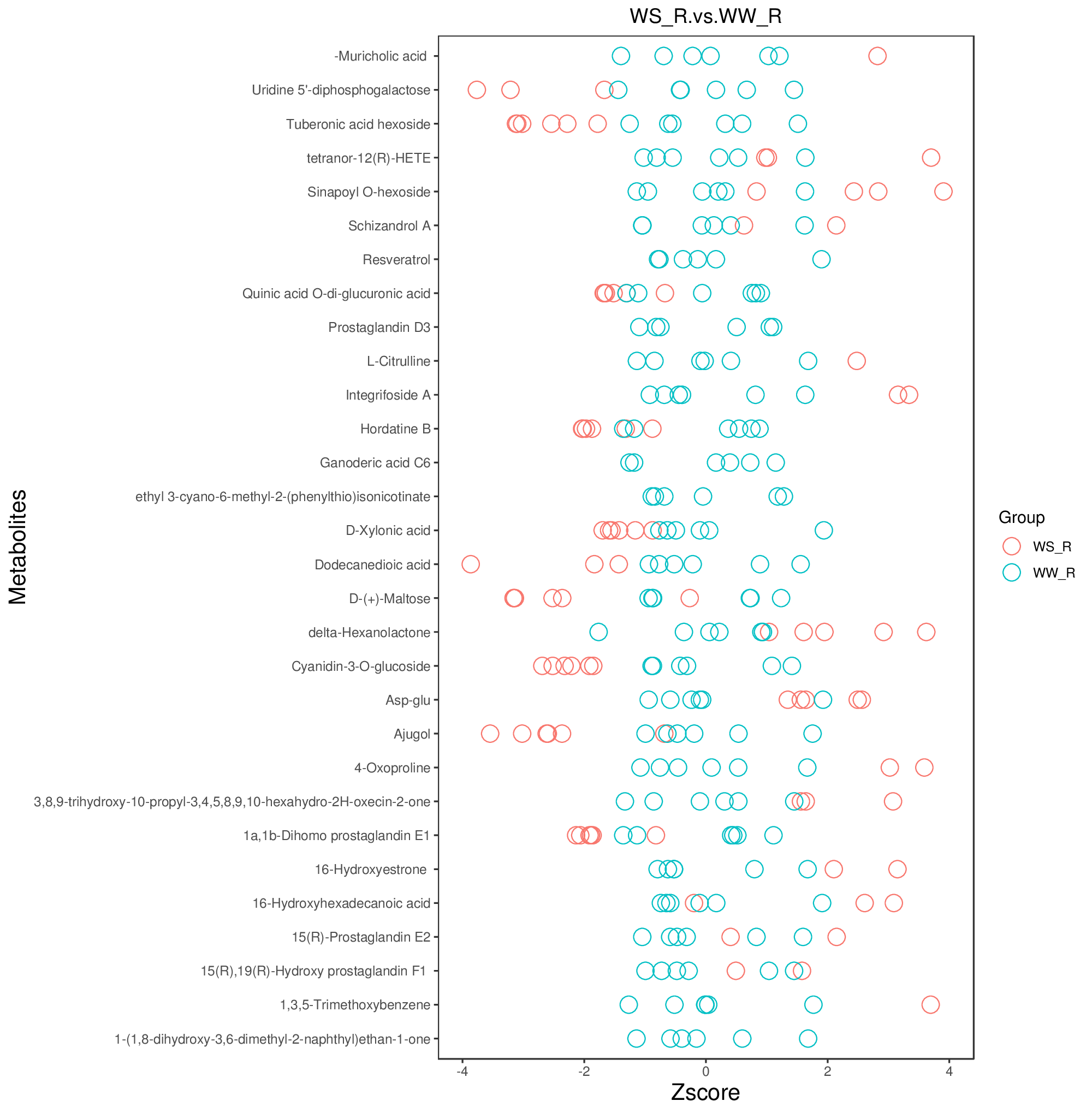

Supplement: Supplementary Figure 1 — PLS-DA scores of experimental groups under negative ion mode. (A) needles (red) vs. roots (blue) of WW seedlings; (B) needles of WS (red) vs.WW (blue) pine seedlings; (C) needles (red) vs. roots (blue) of WS pine seedlings; (D) roots of WS (red) vs. WW (blue) pine seedlings. WW, well-watered; WS, water-stressed. [file DataSheet_1.zip › Supplementary Figures/Fig. S6/Fig. S6D--WS_R.vs.WW_R_neg_zscore.png]

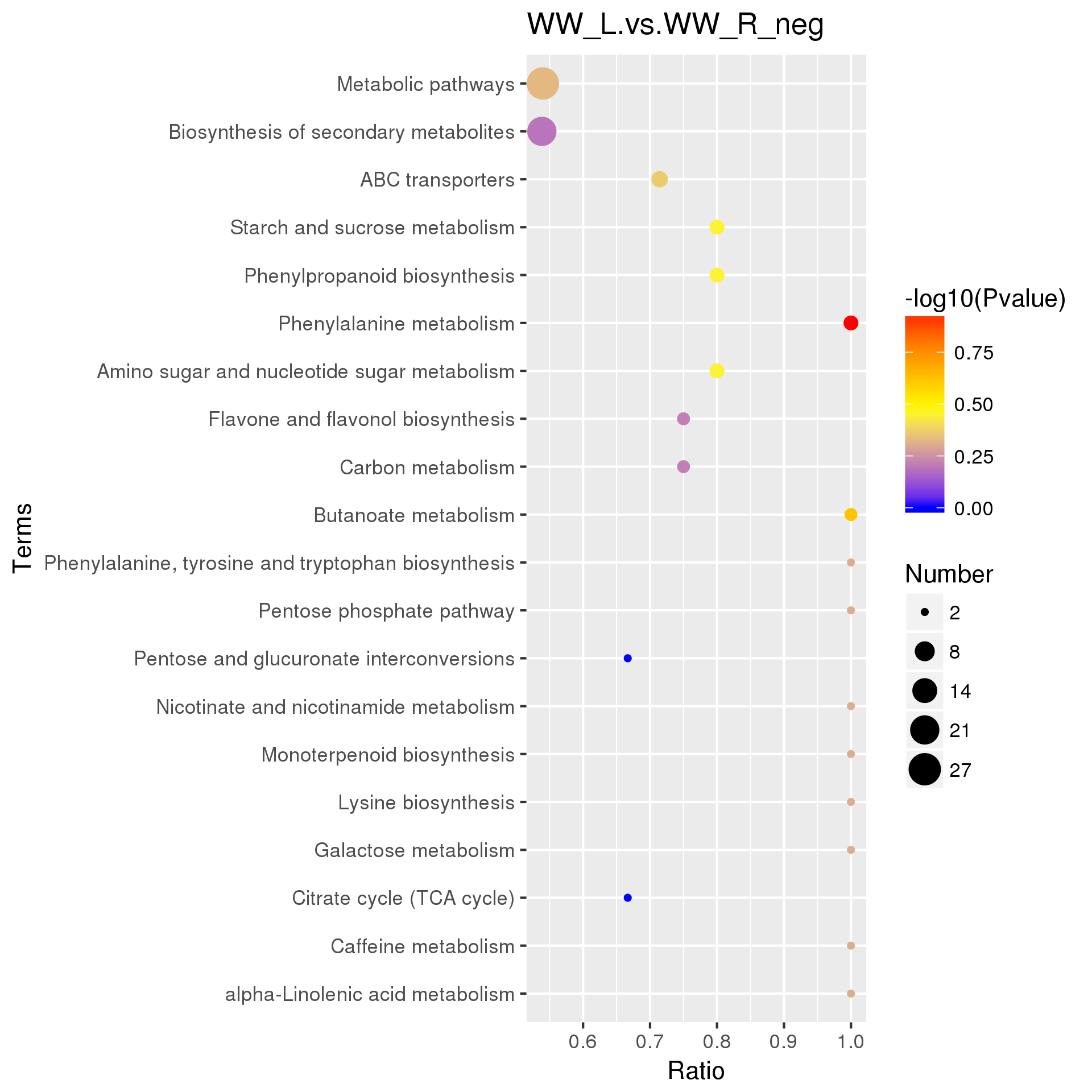

Supplement: Supplementary Figure 1 — PLS-DA scores of experimental groups under negative ion mode. (A) needles (red) vs. roots (blue) of WW seedlings; (B) needles of WS (red) vs.WW (blue) pine seedlings; (C) needles (red) vs. roots (blue) of WS pine seedlings; (D) roots of WS (red) vs. WW (blue) pine seedlings. WW, well-watered; WS, water-stressed. [file DataSheet_1.zip › Supplementary Figures/Fig. S7/Fig. S7A--WW_L.vs.WW_R_neg.KEGG_Enrich.scatterplot.png]

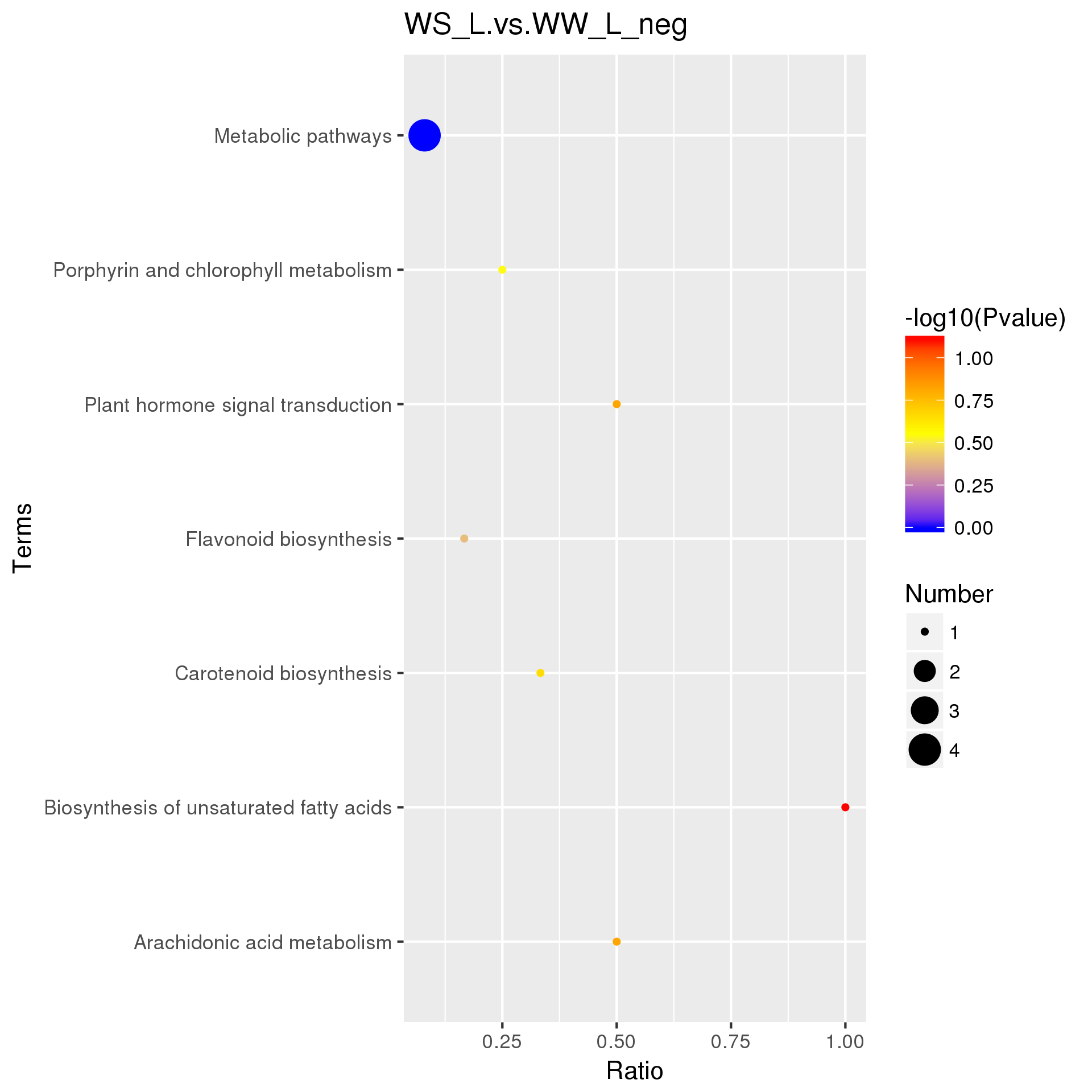

Supplement: Supplementary Figure 1 — PLS-DA scores of experimental groups under negative ion mode. (A) needles (red) vs. roots (blue) of WW seedlings; (B) needles of WS (red) vs.WW (blue) pine seedlings; (C) needles (red) vs. roots (blue) of WS pine seedlings; (D) roots of WS (red) vs. WW (blue) pine seedlings. WW, well-watered; WS, water-stressed. [file DataSheet_1.zip › Supplementary Figures/Fig. S7/Fig. S7B--WS_L.vs.WW_L_neg.KEGG_Enrich.scatterplot.png]

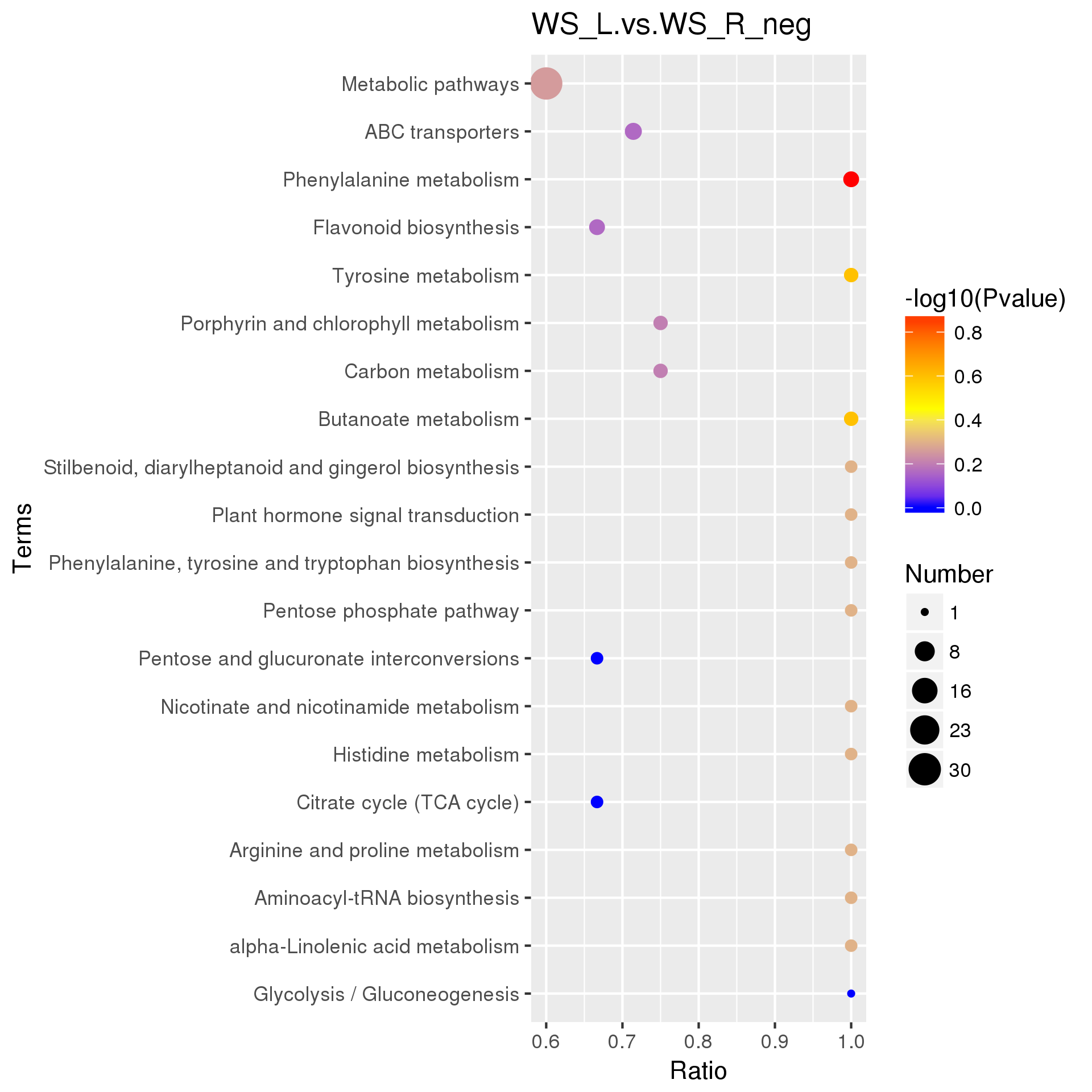

Supplement: Supplementary Figure 1 — PLS-DA scores of experimental groups under negative ion mode. (A) needles (red) vs. roots (blue) of WW seedlings; (B) needles of WS (red) vs.WW (blue) pine seedlings; (C) needles (red) vs. roots (blue) of WS pine seedlings; (D) roots of WS (red) vs. WW (blue) pine seedlings. WW, well-watered; WS, water-stressed. [file DataSheet_1.zip › Supplementary Figures/Fig. S7/Fig. S7C--WS_L.vs.WS_R_neg.KEGG_Enrich.scatterplot.png]

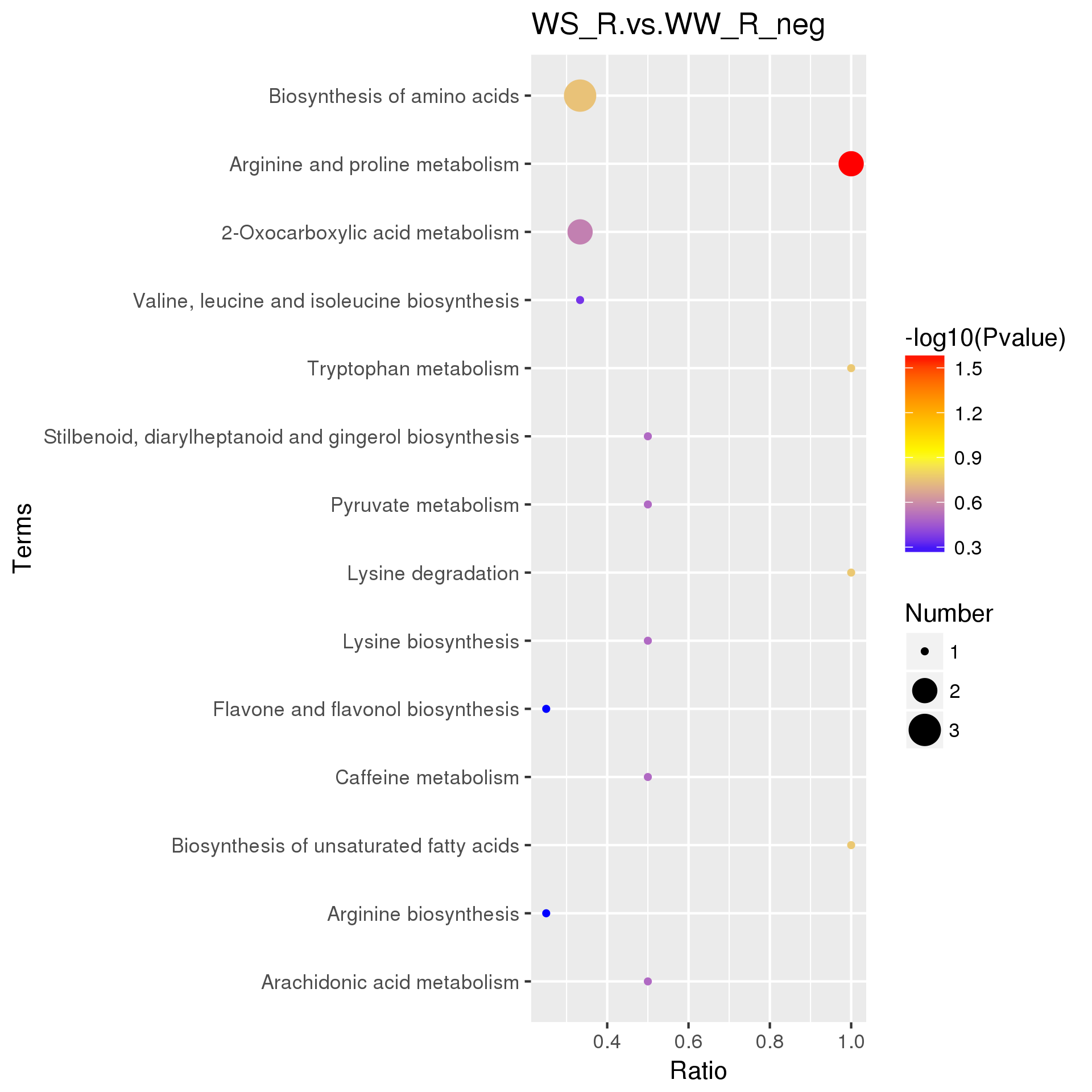

Supplement: Supplementary Figure 1 — PLS-DA scores of experimental groups under negative ion mode. (A) needles (red) vs. roots (blue) of WW seedlings; (B) needles of WS (red) vs.WW (blue) pine seedlings; (C) needles (red) vs. roots (blue) of WS pine seedlings; (D) roots of WS (red) vs. WW (blue) pine seedlings. WW, well-watered; WS, water-stressed. [file DataSheet_1.zip › Supplementary Figures/Fig. S7/Fig. S7D--WS_R.vs.WW_R_neg.KEGG_Enrich.scatterplot.png]
